# *Apiotrichum*‐Centered Fungal Interaction Network Disturbance Correlates With Increased Arterial Stiffness

**DOI:** 10.1111/acel.70650

**Published:** 2026-07-25

**Authors:** Jiarui Chen, Jialin He, Bingqi Ye, Jialu Yang, Ludi Liu, Jingmeng Ju, Sinan Li, Benjie Li, Min Xia, Yan Liu

**Affiliations:** ^1^ Department of Nutrition, School of Public Health Sun Yat‐sen University Guangzhou People's Republic of China; ^2^ Department of Statistics and Epidemiology, School of Public Health Sun Yat‐sen University Guangzhou People's Republic of China

**Keywords:** *Apiotrichum*, arterial stiffness, gut mycobiome, mendelian randomization, multi‐omics integration

## Abstract

The gut mycobiome plays an essential role in human health, yet its influence on the progression of arterial stiffness, a key feature of vascular aging, remains obscure. In this study, we aimed to investigate the involvement of intestinal fungi in the exacerbation of arterial stiffness. In total, 763 participants were invited for the brachial‐ankle pulse wave velocity test to determine their status of arterial stiffness. The gut mycobiome was identified through internal transcribed spacer sequencing, host genetics were determined by genotyping arrays, and plasma metabolites were quantified using untargeted liquid chromatography‐mass spectrometry. A less clustered but more communicative fungal community was found in individuals with elevated stiffness. Five driving fungal genera contributing to the shift of fungal interaction networks from normal to elevated stiffness, including *Apiotrichum*, *Mycothermus*, *Flavocillium*, *Hyaloscypha*, and *Microscypha*, were associated with arterial stiffness. Integrative analysis with metabolomics further demonstrated that organic acids, fatty acyls, and alkaloids mediated the effect of *Apiotrichum*, *Mycothermus*, and *Microscypha* on arterial stiffness. Moreover, alpha‐1A adrenergic receptor, alpha‐2B adrenergic receptor, cytochrome P450 Family 11 subfamily B member 2, angiotensin I converting enzyme, angiotensin II receptor type 1, matrix metallopeptidase 2, tumor necrosis factor, and rho‐associated coiled‐coil containing protein kinase 2 were identified as the key molecular transducers associated with increased *Apiotrichum* in arterial stiffness. Our study uncovers a strong association between disruption in gut fungi and the progression of elevated arterial stiffness, and highlights the potential of targeting the gut mycobiome for improving vascular health.

## Graphical abstract

*Apiotrichum*‐centered gut fungal network disturbance correlates with increased arterial stiffness. Five driver genera including *Apiotrichum*, *Mycothermus*, *Flavocillium*, *Hyaloscypha*, and *Microscypha* regulate the interaction shift. Thirty‐nine metabolites comprising organic acids, fatty acyls, and sucrose target vasoconstriction regulators CYP11B2, ROCK2, MMP2, and alpha‐adrenergic receptors linking gut mycobiome dysbiosis to vascular dysfunction.

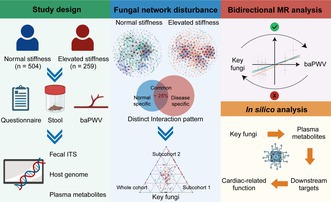

**Abbreviations**
ABI: ankle‐brachial index
ACE: Angiotensin I Converting Enzyme
ADRA1A: Alpha‐1A adrenergic receptor
ADRA2B: alpha‐2B adrenergic receptor
AGTR1: Angiotensin II Receptor Type 1
baPWV: brachial‐ankle pulse wave velocity
BMI: body mass index
CYP11B2: cytochrome P450 family 11 subfamily B member 2
DBP: diastolic blood pressure
eNOS: endothelial nitric oxide synthase
GO: gene ontology
IMT: intima‐media thickness
ITS: Internal Transcribed Spacer
IVW: inverse variance weighted
MAP: mean arterial pressure
MMP2: matrix metalloproteinases 2 protein
MR: Mendelian randomization
NO: nitric oxide
PSM: propensity score matching
ROCK2: rho‐associated coiled‐coil containing protein kinase 2
SBP: systolic blood pressure
SCFA: short‐chain fatty acid
SNPs: single‐nucleotide polymorphism

## 1 Introduction

Elevated arterial stiffness, a hallmark of vascular aging, reflects cumulative damage to the arterial wall and is causally involved in the damage of several target organs, such as brain and kidney (Payne et al. 2010; Van Popele et al. 2001). Pulse wave velocity (PWV), a robust indicator of arterial stiffness and vascular aging, has been identified as an independent risk factor of adverse cardiovascular events (Cunha et al. 2015; Lim et al. 2004; Jae et al. 2021). Of note, established factors, such as age, systolic blood pressure (SBP), and body mass index (BMI), only accounted for a fraction of the variance in PWV (Ghosh et al. 2018), resulting in significant uncontrolled residual risk. Thus, identifying novel modulators and therapeutic strategies for elevated arterial stiffness is essential to alleviate the global disease burden attributable to vascular aging.

Recently, the gut microbiome, a vast collection of microorganisms, including bacteria, viruses, fungi, and archaea, has been identified as an emerging modulator in vascular health (Tang et al. 2017). Disturbance in the taxonomic and functional profiles of the gut microbiome represents a novel source of oxidative stress and inflammation, which in turn triggers the progression of vascular dysfunction (Brandsma et al. 2019). To date, most of the studies exclusively focused on the bacteria (Battson et al. 2019; Menni et al. 2018; Luo et al. 2023), and few projects have examined the role of intestinal fungi, a small but crucial component of the gut microbiome (An et al. 2024), in metabolic health. With an advance in deep sequencing technology, accumulating evidence suggests that fungal species play a pivotal role in the development of various metabolic disorders, such as type 2 diabetes (He et al. 2024) and atherosclerosis (Su et al. 2025). For instance, the complexity of fungal interactions was positively associated with the exacerbation of glycemic control, with *Fusarium* and its related metabolites serving as the key drivers (He et al. 2024). Moreover, the presence of *Ceratocystis* substantially enhanced exercise‐induced amelioration of dyslipidaemia in individuals with prediabetes (Wang et al. 2024). Additionally, *Stereum hirsutum* was reported to delay the progression of atherosclerosis due to its antioxidant and antimicrobial activities (Sevindik et al. 2021). So far, however, no study has yet delineated the exact role of gut fungi in arterial stiffness.

To address this gap, we performed an integrative analysis of the gut mycobiome, host genetics, and plasma metabolites in a cohort of 763 community residents free of antibiotics and dietary supplementation and uncovered the potential molecular mechanisms whereby altered gut mycobiome modulated arterial stiffness.

## 2 Methods

### 2.1 Study Participants

Local residents who had lived in Guangdong province, China, for over 5 years were invited to participate in health screenings conducted at the Community Healthcare Centre of Chashan Town (Dongguan City, Guangdong, China) and Guangzhou (Guangdong, China) through flyers and posters. The detailed inclusion and exclusion criteria were as follows:

*Inclusion criteria*: (i) aged above 18 years; (ii) no severe disabilities, malignant tumors, or acute and chronic inflammatory diseases; (iii) not pregnant; (iv) able to understand the nature and potential consequences of the study; and (v) absence of antibiotics, prebiotics or probiotics within 3 months prior to sample collection.

*Exclusion criteria*: (i) self‐reported or physician diagnosed cardiovascular diseases, such as coronary disease, stroke, and heart failure; (ii) other diseases including biliary obstructive diseases, acute or chronic cholecystitis, acute or chronic viral hepatitis, cirrhosis, diarrhea, hyperthyroidism or hypothyroidism, chronic renal insufficiency, and gastrointestinal diseases.

Finally, a total of 763 participants with internal transcribed spacer 1 (ITS1) sequencing data were included in the analysis, of whom 682 had paired metabolomics data and 752 had host genomics data.

### 2.2 Measurement of Vascular Function Parameters in Study Participants

Multiple vascular function parameters, including brachial‐ankle pulse wave velocity (baPWV), ankle‐brachial index (ABI), and mean arterial pressure (MAP), were simultaneously measured using an automatic waveform analyzer (BP‐203 PRE III, Omron Health Medical, Dalian, China) as previously described (Ato 2018). During the measurement, the room temperature was maintained between 22°C and 25°C. After resting for at least 5 min in a supine position, four cuffs were applied to the upper arms and ankles and connected to a plethysmographic sensor (volume pulse form) and an oscillometric pressure sensor. For MAP, the device automatically calculated values for the right brachial artery (RbaMAP), left brachial artery (LbaMAP), and the average of both sides (baMAP) using an oscillometric method from the brachial cuff pressure waveforms. In the raw data output, these MAP values were recorded in units of 0.1 mmHg and were subsequently converted to mmHg by dividing by 10 for all analyses. The ABI was calculated as the ratio of ankle SBP to brachial SBP. Pressure waveforms were recorded at the brachial and tibial arteries to assess the transmission time between the initial rises of these waves. The baPWV was calculated using the formula (La‐Lb)/Tba. Here, La represents the distance from the heart to the ankle, Lb the distance from the heart to the brachium, and Tba the transmission time between the brachial and posterior tibial artery waveforms. Since baPWV measurements may be biased in patients with severe atherosclerosis in the lower legs, subjects were excluded if bilateral ABI were less than 0.9 or if substantial side differences (greater than 1000 cm/s) were observed. If unilateral ABI was less than 0.9, only the baPWV of the other side was considered as the final reading. Otherwise, the average of the left‐ and right‐side baPWV was used as the final reading. All parameters were measured at least twice per participant. If the difference of baPWV between the 2 measurements was higher than 0.5 m/s, a third measurement was taken. The median value of each parameter was finally recorded.

Carotid ultrasound was performed by an experienced technician using an Aplio 400 system (Toshiba, Japan) equipped with a dedicated intima‐media thickness (IMT) measurement program. This program maintained consistent image acquisition parameters, including postprocessing map, dynamic range, persistence, frame rate, and power output, while transmit gain was adjusted for optimal image quality. Both left and right carotid arteries were scanned. IMT was measured on the far wall of the common carotid artery as determined by the built‐in software algorithm, which automatically calculated the thickness. The presence of a carotid plaque was defined as a focal thickening > 1.2 mm.

Subjects were categorized into groups with elevated arterial stiffness (defined as baPWV ≥ 1400 cm/s) and normal arterial stiffness (baPWV < 1400 cm/s) (Takashima et al. 2014). For sensitivity analysis in network construction, sub‐cohorts of elevated arterial stiffness and normal arterial stiffness were established via two different propensity score matching strategies using bias‐corrected logistic regression model: one matched on age, sex, and BMI, and the other additionally matched on medication usage. The caliper width for propensity matching was restricted to 0.2 of the standard deviation of the logit to ensure optimal pair matching.

### 2.3 Collection of Other Covariates and Biological Samples

Anthropometric parameters, including body weight, height, SBP, and diastolic blood pressure (DBP), were collected by trained staff. Blood pressure measurements were taken on the right upper arm while the subject was in the sitting position after at least 10–15 min of rest, using a validated digital automatic analyzer (Omron HEM‐7136). A comprehensive questionnaire including demographic characteristics, medical history, current medication, and lifestyles was performed by trained staff in a face‐to‐face manner. The dietary diversity score was evaluated based on a food frequency questionnaire including 9 food groups, namely vegetables, fruits, nuts, meat, eggs, fish, milk, dairy products, and tea, as previously described (Yin et al. 2017). The 10‐year cardiovascular disease risk was calculated using the Prediction for Atherosclerotic Cardiovascular Disease Risk in China (China‐PAR) equation (Yang et al. 2016).

Fecal samples were collected using the MGIEasy stool collection kit, which contains a room‐temperature stable reagent, and were stored in a central freezer at −80°C until analysis. Plasma samples were collected on the same day as the fecal samples, in the morning following a fast of approximately 10–12 h, between 8:00 and 9:00 a.m.

### 2.4 Fecal ITS1 Sequencing

DNA extraction from fecal samples was conducted by the MagMAX Microbiome Ultra Nucleic Acid Isolation Kit (Thermo Fisher Scientific, MA, USA). The concentration and purity of the extracted DNA were further evaluated by using 1% agarose gels. Internal transcribed spacer 1 (primers: sense 5′‐GGAAGTAAAAGTCGTAACAAGG‐3′ and antisense 5′‐GCTGCGTTCTTCATCGATGC‐3′) sequencing was implemented by using the Illumina NovaSeq platform (Illumina, San Diego, USA) by Novogene Co. Ltd. (Beijing, China) to generate 250‐bp paired‐end reads.

### 2.5 Human Genomics

Genomic DNA extraction from buffy coat samples employed the TIANamp Blood DNA Kit (TIANGEN, Beijing, China) in strict accordance with manufacturer protocols. For library construction, input DNA quantities exceeded 1 μg with concentrations standardized to ≥ 80 ng/μL. All genotyping utilized the Infinium Chinese Genotyping Array‐24 v1.0 BeadChip (Illumina platform), conducted by WeGene Co. Ltd. (Shenzhen, China).

### 2.6 Metabolomics Profiling in Study Participants

Widely targeted metabolomic analysis was conducted by Metware (Wuhan, China) using their integrated platform (Chen et al. 2013) which enabled large‐scale detection, identification, and quantification of metabolites, with protocol modifications detailed in Supporting Information S1.

### 2.7 Statistical Analysis

All statistical analyses were performed with R software (version 4.4.1). Frequency counts with percentages described categorical variables, analyzed by Chi‐Square tests. Continuous variable normality was evaluated using Shapiro–Wilk test. These data were further summarized as medians with interquartile ranges (IQRs) and compared via Wilcoxon rank‐sum tests. All multiple comparisons incorporated Benjamini‐Hochberg false discovery rate correction (*p*_*adj*_), except where specified. Supporting Information S1 provide expanded multi‐omics analytical details.

## 3 Results

### 3.1 Characterization of Gut Mycobiome in Individuals With Normal or Elevated Arterial Stiffness

Overall, the median age of study participants was 50 years, 55% were female, and 259 subjects had elevated arterial stiffness. As expected, individuals with elevated arterial stiffness were older, more likely to be male and overweight, had a higher blood pressure and worse lipid profiles. Moreover, several established risk factors for adverse cardiovascular events, such as the mean arterial pressure, intima‐media thickness, ABI, and pulse pressure (PP), were substantially higher in subjects with elevated arterial stiffness compared to normal arterial stiffness (Table 1). Of note, PP is an established marker of large artery stiffness, and its marked elevation in the high‐stiffness group further reinforced the classification based on baPWV. We then explored the involvement of gut mycobiome in arterial stiffness using ITS sequencing in fecal samples. A total of 304 genera from 13 phyla were found in this cohort. The predominant fungal phylum was *Ascomycota* (25.2%), followed by *Basidiomycota* (22.9%), and *Mortierellomycota* in both groups (14.8%, Figure S1A). At the genus level, the three most abundant genera were *Candida*, *Aspergillus*, and *Issatchenkia*, with mean relative abundances of 2.97%, 2.63%, and 2.27%, respectively (Figure S1B). Contrary to previous findings in other metabolic disorders, no significant differences in alpha and beta diversity were observed between subjects with normal and elevated arterial stiffness (Figure S1C,D).

**TABLE 1:** Basic characteristics of the study participants.

Characteristic	Total subjects	Normal Arterial Stiffness	Elevated Arterial Stiffness	*p*
	*N* = 763	*N* = 504	*N* = 259	
Clinical characteristics				
Male, *n* (%)	344 (45.09%)	194 (38.49%)	150 (57.92%)	< 0.001
Age, years	50 [45, 56]	48 [43, 54]	55 [49, 62]	< 0.001
BMI, kg/m^2^	23.90 [21.60, 25.90]	23.50 [21.30, 25.60]	24.30 [22.70, 26.25]	< 0.001
Dietary diversity	5 [5, 6]	5 [5, 6]	5 [5, 6]	0.499
Vascular function measurements				
RbaPWV, cm/s	1307.00 [1172.00, 1488.50]	1207.00 [1106.00, 1305.00]	1572.00 [1485.50, 1688.00]	< 0.001
LbaPWV, cm/s	1301.00 [1158.00, 1469.00]	1208.50 [1103.00, 1298.25]	1553.00 [1468.00, 1687.50]	< 0.001
baPWV, cm/s	1310.50 [1166.25, 1481.75]	1207.50 [1109.00, 1307.12]	1565.50 [1480.25, 1683.75]	< 0.001
RABI	1.10 [1.05, 1.14]	1.09 [1.04, 1.13]	1.12 [1.07, 1.16]	< 0.001
LABI	1.09 [1.05, 1.14]	1.08 [1.04, 1.13]	1.12 [1.07, 1.16]	< 0.001
ABI	1.10 [1.05, 1.14]	1.09 [1.04, 1.12]	1.12 [1.08, 1.16]	< 0.001
RbaMAP, mmHg	93.40 [84.28, 102.05]	88.48 [80.75, 94.66]	104.20 [98.05, 111.73]	< 0.001
LbaMAP, mmHg	93.00 [83.90, 101.65]	87.50 [80.60, 94.85]	103.50 [97.90, 111.66]	< 0.001
baMAP, mmHg	93.43 [84.45, 101.33]	87.84 [80.42, 94.69]	103.83 [98.24, 111.39]	< 0.001
Plaque, *n* (%)	0 [0, 0]	0 [0, 0]	0 [0, 1]	< 0.001
IMT, mm	0.70 [0.50, 0.90]	0.60 [0.50, 0.80]	0.80 [0.60, 1.10]	< 0.001
China‐Par	1.68 [0.77, 3.52]	1.06 [0.54, 2.14]	3.91 [2.34, 6.55]	< 0.001
Laboratory measurements				
SBP, mmHg	122.50 [112.50, 133.50]	116.50 [109.00, 125.50]	134.50 [125.75, 148.00]	< 0.001
DBP, mmHg	79.50 [73.50, 86.50]	77.00 [71.00, 83.50]	85.00 [79.00, 93.00]	< 0.001
PP, mmHg	42.5 [36.50, 49.50]	39.50 [35.00, 45.50]	50.00 [42.75, 57.00]	< 0.001
FG, mmol/L	4.72 [4.30, 5.20]	4.62 [4.24, 5.05]	5.00 [4.49, 5.52]	< 0.001
TG, mmol/L	1.15 [0.80, 1.63]	1.07 [0.76, 1.50]	1.30 [0.96, 1.96]	< 0.001
TC, mmol/L	5.28 [4.76, 5.95]	5.20 [4.67, 5.88]	5.48 [4.94, 6.19]	< 0.001
HDL‐c, mmol/L	1.39 [1.19, 1.65]	1.44 [1.23, 1.68]	1.33 [1.10, 1.58]	< 0.001
LDL‐c, mmol/L	3.16 [2.72, 3.68]	3.09 [2.62, 3.64]	3.26 [2.86, 3.79]	0.003
CREA, μmol/L	74.70 [64.90, 87.60]	72.40 [63.48, 85.23]	79.40 [67.85, 94.70]	< 0.001
Medication usage				
Anti‐hypertensive drugs, *n* (%)	102 (13.37%)	38 (7.54%)	64 (24.71%)	< 0.001
Anti‐diabetic drugs, *n* (%)	36 (4.72%)	11 (2.18%)	25 (9.65%)	< 0.001
Hypolipidemic agents, *n* (%)	23 (3.01%)	10 (1.98%)	13 (5.02%)	0.036

*Note:* Data were shown as median (interquartile range) or number (percentage). *p* value represented the difference between groups in percent (%) or median (IQR).

### 3.2 Divergent Interaction Patterns of Gut Fungi in Subjects With Normal and Elevated Arterial Stiffness

**FIGURE 1:**
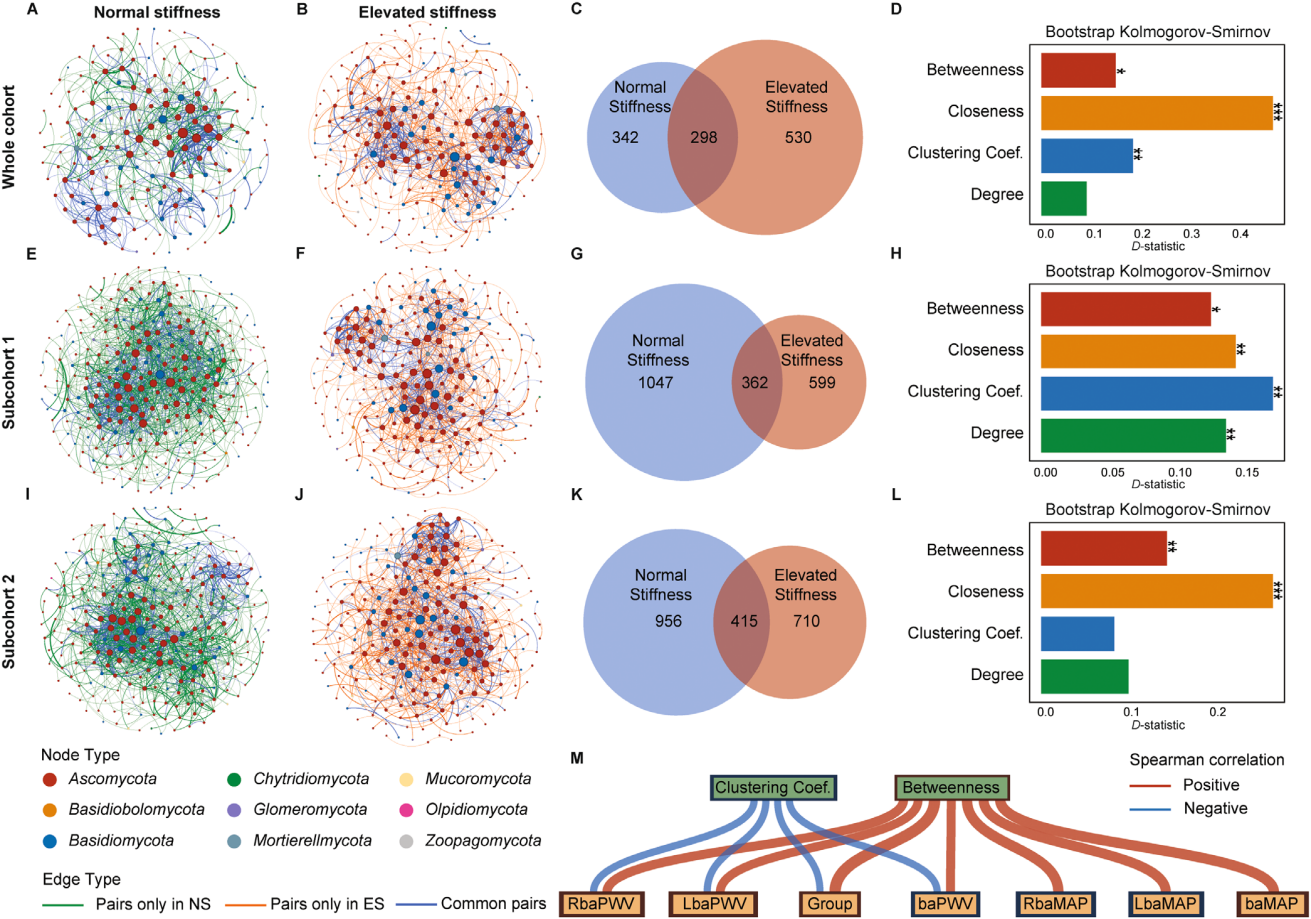
Distinct fungal interaction patterns with the progression of elevated arterial stiffness. Co‐abundant networks based on significant Spearman correlations among gut fungi in subjects with normal (A, E, I) and elevated (B, F, J) arterial stiffness within the whole cohort (*n* = 504 for normal stiffness, *n* = 259 for elevated stiffness) (A, B), sub‐cohort 1 (matched on age, sex, BMI; *n* = 204 per group) (E, F), and sub‐cohort 2 (additionally matched on medication usage; *n* = 200 per group) (I, J), respectively. Edges represent significant correlations (*p*_adj_ < 0.05) with an absolute Spearman's rho ≥ 0.3. Edge colors indicate correlations unique to the normal stiffness group (green), unique to the elevated stiffness group (red), or common to both (blue). Venn diagrams showing the number of unique and shared correlation pairs in the whole cohort (C), sub‐cohort 1 (G), and sub‐cohort 2 (K). Comparison of topological parameters between groups via boostrap Komogorov‐Smirnov test within the whole cohort (D), sub‐cohort 1 (H), and sub‐cohort 2 (L). (M) Sankey diagram illustrating significant Spearman correlations (*p*_adj_ < 0.05) between topological features and clinical phenotypes in the whole cohort; red and blue represent positive and negative correlations, respectively.

Given that individual taxa within the microbial consortia are ecologically linked and the complex microbial ecosystem is unevenly influenced by individual taxa, co‐abundance analysis at genus level was further performed. Intriguingly, there was a distinct interaction pattern in the mycobiome between subjects with normal and elevated arterial stiffness. Specifically, 640 and 828 significant positive correlations were found in the fungal community of individuals with normal and elevated arterial stiffness, respectively. Only 298 pairs of the interactions were shared in both groups, while over 50% of the interactions were unique within each group (Figure 1A–C). Consistently, the distribution of node betweenness, node closeness, and clustering coefficient was significantly different between the two groups (*p* < 0.05), indicating a distinct network structure of the gut mycobiome in subjects with or without elevated arterial stiffness (Figure 1D and Figure S2A). To further exclude the potential confounding effect of imbalance in sample size, sex, age, and BMI in each group on the association of fungal interaction network with elevated arterial stiffness, individuals with or without elevated arterial stiffness were propensity matched (Table S1). Similarly, the majority of the significant fungal interaction pairs were specific within each group, and a distinct distribution of all topology indicators was found between the fungal interaction networks in individuals with normal and elevated arterial stiffness (Figure 1E–H; Figure S2B). Additionally, when the potential effect of medication usage on gut mycobiome was further taken into consideration, the distinct patterns of fungal interaction networks in the community of individuals with normal and elevated arterial stiffness remained largely the same (Table S2; Figure 1I–L; Figure S2C). Of note, the global clustering coefficient, an indicator of network cohesion and interconnectivity (Kajihara and Hynson 2024), was inversely associated with baPWV, while the node betweenness, an indicator of communication across nodes (Kajihara and Hynson 2024), was positively correlated with both PWV and mean arterial pressure (Figure 1M; Table S3), indicating a less clustered but more communicative fungal community in individuals with elevated arterial stiffness.

**FIGURE 2:**
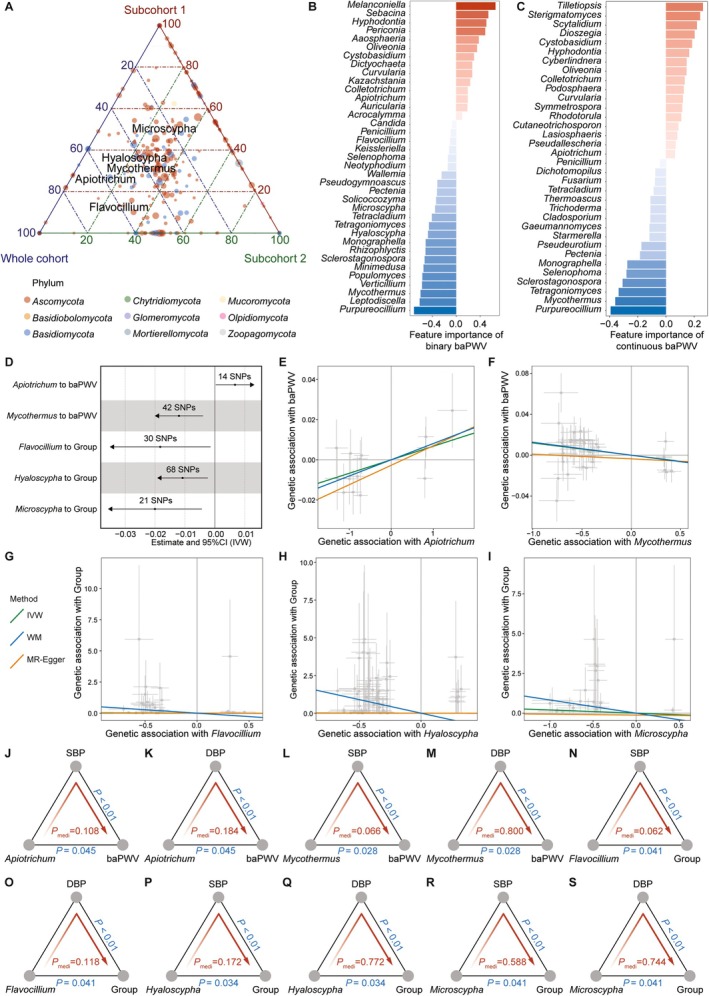
Key fungi in the progression of elevated arterial stiffness. (A) Ternary plot illustrating the NetMoss scores of fungal genera driving the interaction network transition from normal to elevated arterial stiffness across the whole cohort (*n* = 763), sub‐cohort 1 (*n* = 408, matched on age, sex, BMI) and sub‐cohort 2 (*n* = 400, additionally matched on medication usage). Node color represents phylum affiliation; node size corresponds to the mean genus abundance in the whole cohort. Bar charts show fungal genera significantly associated with (B) elevated arterial stiffness and (C) continuous baPWV, identified via MaAsLin2 analysis, adjusted for age, sex, BMI, and medication usage (*p*_adj_ < 0.05, *n* = 763). Blue and red bars indicate enrichment in normal or elevated stiffness groups, respectively. (D) Forest plot presenting Mendelian randomization estimates (beta values with 95% CIs) of causal associations between key fungi and baPWV or arterial stiffness. Scatter plots depict SNP effects on (E) *Apiotrichum*, (F) *Mycothermus*, (G) *Flavocillium*, (H) *Hyaloscypha*, and (I) *Microscypha* against effects on baPWV or stiffness; line slope represents MR effect sizes. (J–S) Mediation analysis depicting mediating pathways linking key fungal genera to arterial stiffness and continuous baPWV through SBP or DBP. Path coefficients, *p* values and proportion of the mediation effects to total effects were denoted beside each path.

Furthermore, the NetMoss score was calculated for each node to identify the driving fungal genera contributing to the shift of interaction networks from normal to elevated arterial stiffness. A total of 223, 249, and 260 fungal genera were identified as significant features for the network shift from normal to elevated arterial stiffness in the whole cohort and two propensity‐matched sub‐cohorts, respectively (Figure 2A, *p*_adj_ < 0.05). Among them, the 160 overlapping taxa, mainly from *Ascomycota*, *Basidiomycota*, *Mucoromycota*, and *Glomeromycota* genera (Figure 2A), were considered as important features responsible for the network shifts with the progression of elevated arterial stiffness. Of the 160 key fungal taxa, a total of 37 fungal taxa, mainly from the *Ascomycota* phylum, were differentially enriched between subjects with normal or elevated arterial stiffness, after adjustment for sex, age, BMI, and medication usage (*p*_adj_ < 0.05, Figure 2B). *Melanconiella*, *Sebacina*, and *Hyphodontia* ranked among the top three fungi taxa demonstrating a strong positive association with elevated arterial stiffness, while *Purpureocillium*, *Leptodiscella*, and *Mycothermus* showed the most remarkable negative correlation with enhanced arterial stiffness (*p*_adj_ < 0.05, Figure 2B). Given that categorical data may limit our understanding of the true patterns of association, we further examined the relevance of fungal taxa in continuous baPWV. Among the 34 fungal taxa significantly associated with baPWV, *Tilletiopsis*, a genus from the *Basidiomycota* phylum which had been found to be upregulated in type 2 diabetes (Zhou et al. 2023), demonstrated the strongest positive association with baPWV. Conversely, *Purpureocillium*, a genus within the phylum *Ascomycota* capable of producing secondary metabolites with anticancer and antimicrobial properties (Chen and Hu 2021), was inversely associated with baPWV (*p*_adj_ < 0.05, Figure 2C). Taken together, the above findings demonstrated that key fungi taxa driving the shifts of the interaction networks were closely associated with the development of elevated arterial stiffness.

### 3.3 Potential Causal Links Between Important Fungus Taxa and the Progression of Elevated Arterial Stiffness

To establish potential causal relationships between key fungal taxa and arterial stiffness progression, we implemented bidirectional Mendelian randomization (MR) analysis. Among the 160 candidate important fungi, 5 genera demonstrated potential causal links with increased arterial stiffness: *Apiotrichum* with promotive effect, *Mycothermus*, *Flavocillium*, *Hyaloscypha*, and *Microscypha* with protective effects (Figure 2D–I; Tables S4 and S5). In detail, inverse variance weighted (IVW) estimates revealed *Apiotrichum* significantly increased baPWV (Beta_IVW_ = 0.007, 95% CI = 0.003 to 0.010, *p* < 0.05). Conversely, *Mycothermus* (Beta_IVW_ = −0.012, 95% CI = −0.016 to −0.008, *p* < 0.05) reduced arterial stiffness, with *Flavocillium* (Beta_IVW_ = −0.018, 95% CI = −0.027 to −0.010, *p* < 0.05), *Hyaloscypha* (Beta_IVW_ = −0.011, 95% CI = −0.015 to −0.006, *p* < 0.05), and *Microscypha* (Beta_IVW_ = −0.020, 95% CI = −0.028 to −0.012, *p* < 0.05) also demonstrating significant protective effects (Figure 2D). Robustness of the above results was further validated through multiple approaches: First, consistent effect directions were observed across the other two MR methods (Figure 2E–I). Second, all instrumental variables exceeded the critical threshold (F‐statistics > 10), eliminating weak instrument bias. Furthermore, MR‐Egger regression detected no significant horizontal pleiotropy (intercept *p* > 0.05; Table S5). Finally, leave‐one‐out analysis confirmed that no single nucleotide polymorphism drove the observed association (Figure S3). Moreover, reverse MR analysis indicated no potential causal effects of arterial stiffness on *Flavocillium* or *Microscypha*. Although significant associations appeared for *Apiotrichum*, *Mycothermus*, and *Hyaloscypha* in IVW analysis, significant MR‐Egger intercept terms (Figure S4, Table S6) invalidated these reverse causal interpretations. Collectively, the above findings demonstrated that *Apiotrichum*, *Mycothermus*, *Flavocillium*, *Hyaloscypha*, and *Microscypha* presented as five important fungal genera leading to the elevation of arterial stiffness.

### 3.4 Circulating Metabolites Serve as Crucial Mediators Linking Altered Gut Mycobiome to Elevated Arterial Stiffness

**FIGURE 3:**
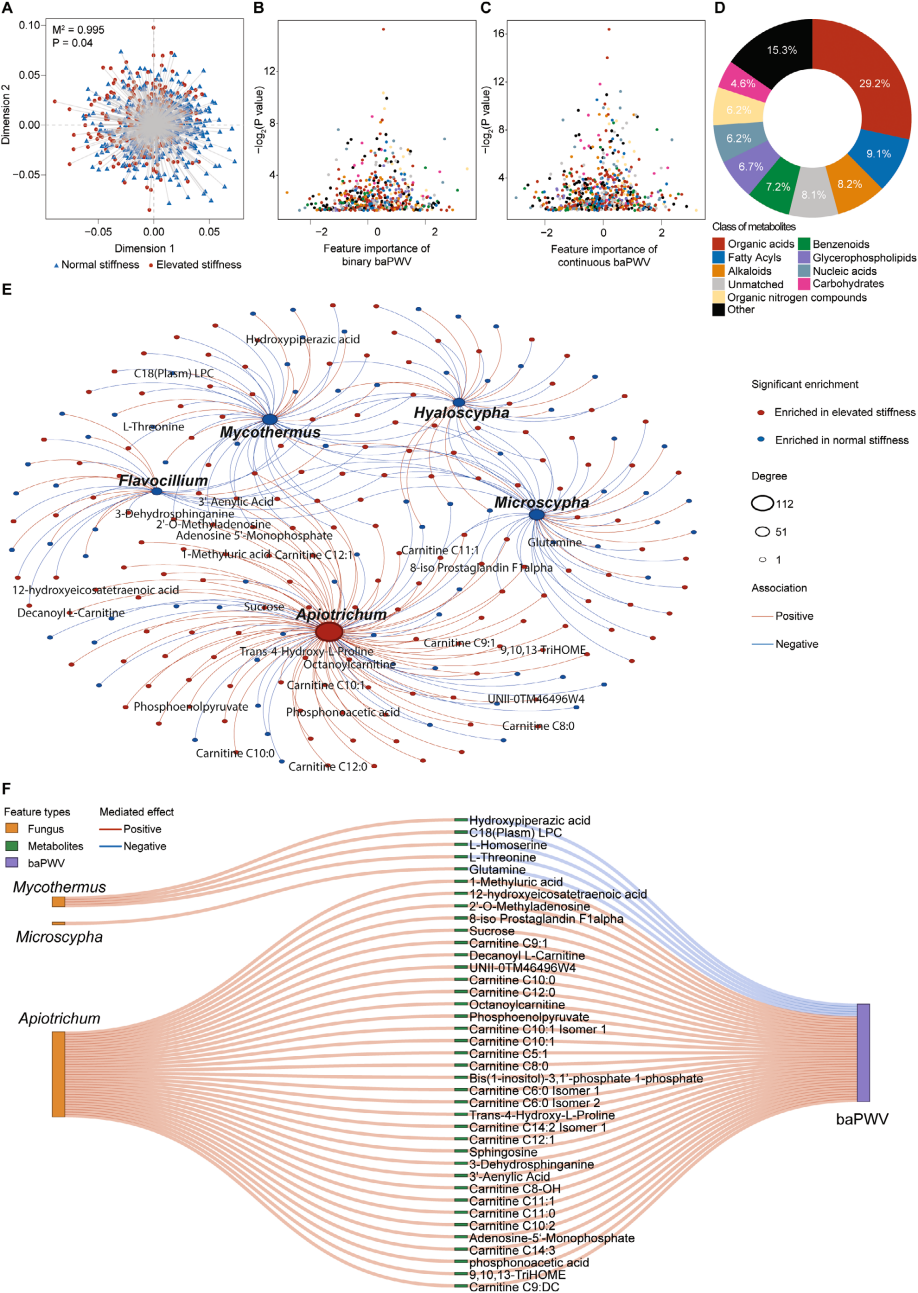
Plasma metabolites mediate the effects of key fungal genera on arterial stiffness. (A) Procrustes analysis (*n* = 682 participants with paired metabolomics data) reveals concordance between gut fungal and plasma metabolite profiles. Points are colored and shaped by arterial stiffness status (blue triangle for normal stiffness, and red circle for elevated stiffness); gray lines connect paired fungal and metabolite samples from the same individual. Metabolites significantly associated with (B) elevated arterial stiffness and (C) continuous baPWV, identified by Maaslin2 (*p*_adj_ < 0.05, *n* = 763). (D) Pie chart displaying the superclass distribution (Class I) of significant metabolites pooled from (B, C). (E) Correlation network illustrating significant Spearman associations (*p*_adj_ < 0.05) between key fungal genera and metabolites. Node size represents degree; node color indicates enrichment group (blue for normal stiffness, and red for elevated stiffness); edge color denotes correlation direction (red for positive, and blue for negative). (F) Sankey diagram visualizing mediating pathways linking key fungi to baPWV via metabolites. Red and blue lines represent positive and negative associations, respectively.

Given that blood pressure was a strong predictor for the progression of elevated arterial stiffness (Levy et al. 2007), we first evaluated whether the key fungi affected arterial stiffness via the modulation of blood pressure. Unexpectedly, neither SBP nor DBP accounted for the effect of *Apiotrichum*, *Mycothermus*, *Flavocillium*, *Hyaloscypha*, and *Microscypha* on arterial stiffness (Figure 2J–S), suggesting that other mechanisms other than blood pressure mediate the modulatory effect of key fungi on arterial stiffness. As small molecular metabolites always function as microbial effectors in the modulation of metabolic health, we then interrogated whether and how circulating metabolites mediated the effect of key fungi on arterial stiffness through an integrative analysis. Procrustes analysis demonstrated a significant covariance between the gut mycobiome and plasma metabolites (*M* ^2^ = 0.995, *p* < 0.05; Figure 3A), suggesting that alterations in the mycobiome interactions may influence circulating metabolites. After adjustment for sex, age, BMI, and medication usage, 708 and 583 metabolites, mainly spanning organic acids (29.2%), fatty acyls (9.1%), and alkaloids (8.2%), were found to be closely associated with elevated arterial stiffness and baPWV, respectively (Figure 3B–D). We first focused on short‐chain fatty acids (SCFAs), given their established roles in gut microbial metabolism and host metabolic health. Among the 13 metabolites annotated as SCFAs, 8 of them showed significance between the two stiffness groups (*p*_*FDR*_ < 0.05): γ‐Hydroxybutyric acid, 2‐hydroxy‐2‐methylbutyric acid, 2‐amino‐4‐oxovaleric acid, 2‐hydroxyisocaproic acid, and 2‐ethyl‐2‐hydroxybutyric acid were higher, while 2‐hydroxyhexanoic acid, 5‐hydroxyhexanoic acid, and (S)‐leucic acid were decreased in the elevated stiffness group, with consistent correlations to multiple cardiovascular parameters (Figure S5A–C). Among all the significant metabolites, 81 metabolites mainly from organic acids demonstrated a positive association with *Apiotrichum*. On the contrary, 37, 17, 19, and 40 metabolites, mainly from organic acids (*n* = 47) and fatty acyls (*n* = 19), were primarily negatively correlated with *Mycothermus*, *Flavocillium*, *Hyaloscypha*, and *Microscypha*, respectively (Figure 3E). However, for SCFAs, only several correlations with *Microscypha* and *Flavocillium* were observed at a relaxed threshold (*p*_*FDR*_ < 0.2), and the direction of these associations was only partially consistent with the baPWV‐related changes (Figure S5C). Mediation analysis further demonstrated that only 39 metabolites functioned as significant mediators linking increased *Apiotrichum* but decreased *Mycothermus* and *Microscypha* on elevated arterial stiffness (Figure 3F; Table S7), whereas none of the SCFAs exhibited a significant mediation effect for any fungal genus. More specifically, glutamate was the only one metabolite that mediated the protective effect of *Microscypha* against increasing baPWV, accounting for 12.34% of the effect. Similarly, L‐threonine, L‐homoserine, hydroxypiperazic acid, and C18(Plasm) LPC explained 13.03%–22.10% of the favorable effect of *Mycothermus* on arterial stiffness. On the contrary, of the 19 metabolites, mainly from carnitine species and organic acids, Carnitine C5:1 explained the highest proportion of the adverse effect of *Apiotrichum* on arterial stiffness, followed by Carnitine C11:1 and Adenosine 5′‐Monophosphate (Figure 3F; Table S7).

**FIGURE 4:**
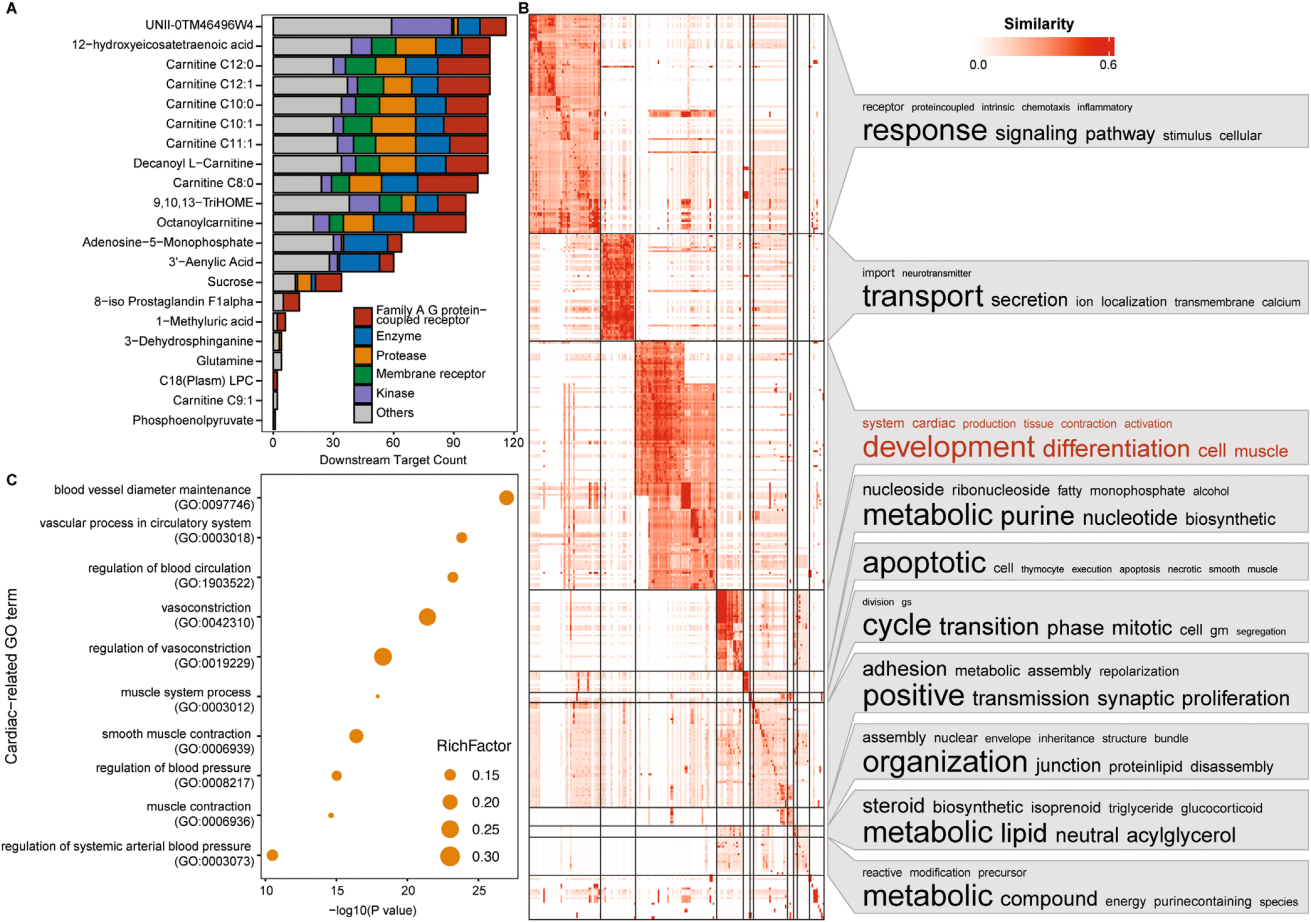
Downstream targets of key metabolites. (A) Bar plot indicating the number of downstream targets per metabolite (*n* = 459 targets in total), colored by target category. Targets were predicted using SwissTargetPrediction with a probability threshold > 0.1. (B) Similarity clustering heatmap of enriched pathways with term frequency represented by font size. (C) Top 10 significantly enriched GO terms derived from the largest cluster in (B), which is primarily related to cardiac function. Node size represents the rich factor.

**FIGURE 5:**
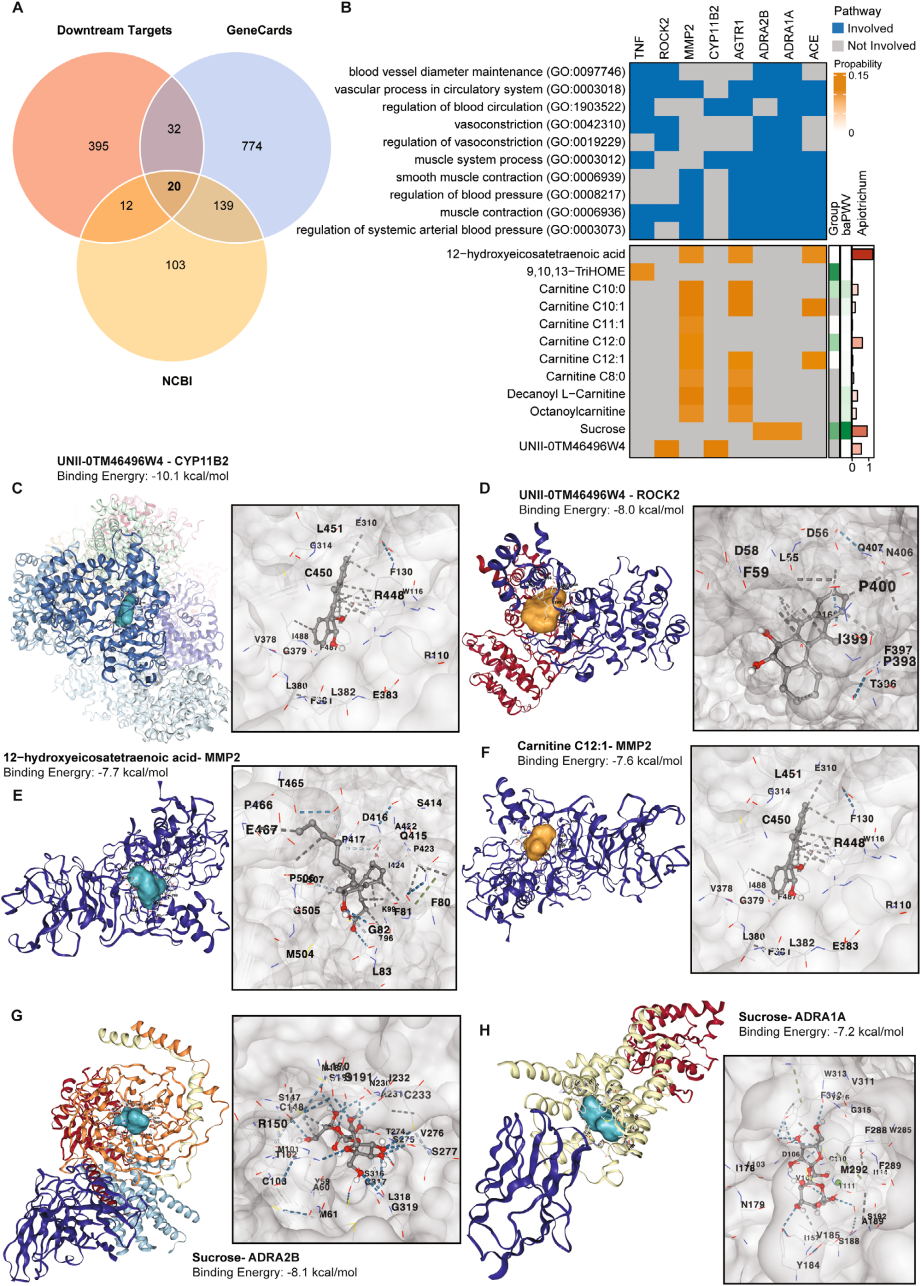
Binding affinity of the fungi‐related metabolites to targets associated with arterial stiffness. (A) Overlap between potential downstream targets of metabolites with significant mediation effects (*n* = 459) and arterial stiffness‐related genes curated from NCBI (*n* = 274) and GeneCards (*n* = 965) repositories. (B) Heatmap showing the targets involved in the top 10 pathways related to cardiac function (retrieved from Figure 4C) and their probability as downstream targets for each metabolite, with reference to the gene sets in (A). The heatmap in the right showing the correlation coefficient between each metabolite and elevated arterial stiffness and baPWV, respectively. The bar chart in the right represented the correlation coefficient between *Apiotrichum* and each metabolite. (C–H) The top binding site and binding energy between each metabolite and the corresponding downstream targets. A binding energy < −7.0 kcal/mol was considered indicative of highly stable binding. For each metabolite‐target pair, the 3D interaction mode is shown, with potential binding sites displayed.

Moreover, of the 459 downstream targets of these metabolites with significant mediation effects, most of them belonged to enzymes (16.99%), family A G protein‐coupled receptors (15.47%), and kinase (12.64%, Figure 4A; Table S8). Gene Ontology (GO) enrichment analysis further suggested that the downstream targets of these metabolites were primarily involved in the differentiation and development of muscle cells related to cardiac system (Figure 4B), such as blood vessel diameter maintenance, vascular process in circulatory system, regulation of blood circulation, and vasoconstriction (Figure 4C). This result suggested that vasoconstriction increases vascular smooth muscle tone, which reduces blood vessel diameter and arterial compliance. Lower compliance accelerates pulse wave propagation, thereby directly elevating baPWV. The regulation of blood circulation and vascular processes further coordinate systemic hemodynamic changes that amplify this effect. These findings further indicate that increased *Apiotrichum* but decreased *Mycothermus* and *Microscypha* may correlate with the elevated arterial stiffness via various muscular and vascular processes. Moreover, 20 of the 459 potential downstream targets, such as fatty acid binding protein 4, matrix metallopeptidase 9, and retinol binding protein 4, had been previously reported to be involved in the modulation of arterial stiffness in both NCBI and GeneCard databases (Figure 5A). Of the 20 putative targets, alpha‐1A adrenergic receptor (ADRA1A), alpha‐2B adrenergic receptor (ADRA2B), cytochrome P450 Family 11 subfamily B member 2 (CYP11B2), angiotensin II receptor type 1 (AGTR1), matrix metallopeptidase 2 (MMP2), and rho associated coiled‐coil containing protein kinase 2 (ROCK2) were found to be involved in the top 10 enriched pathways related to vascular process and vasoconstriction (Figure 5B). Of note, all these targets were the downstream regulators of metabolites which mediated the detrimental effect of *Apiotrichum* on arterial stiffness. For instance, carnitine species were primarily associated with MMP2, an enzyme capable of cleaving components of the extracellular matrix (Zordoky et al. 2015), and angiotensin I converting enzyme (ACE), a key enzyme in renin‐angiotensin system (Kosacka et al. 2022). Additionally, 12−hydroxyeicosatetraenoic acid was closely associated with MMP2, ACE and AGTR1, while trans−5,6−Dihydro−5,6−dihydroxy−7,12−dimethylbenz[a]anthracene (UNII‐0TM46496W4) was primarily related to CYP11B2 and ROCK2. Importantly, the numerical associations of *Apiotrichum*‐related metabolites with potential downstream targets were further supported by the molecular docking analysis. The majority of the metabolites demonstrated a binding energy lower than −5.0 kcal/mol with the corresponding targets, indicating a relatively steady interaction (Table S11). Among them, 6 highly stable bindings with a binding energy less than −7 kcal/mol were observed. For example, UNII‐0TM46496W4 demonstrated a strong binding capacity with CYP11B2 and ROCK2 with a binding energy of −10.1 kcal/mol and −8.0 kcal/mol, respectively (Figure 5C,D). Similarly, 12‐hydroxyeicosatetraenoic acid and Carnitine C 12:1 showed high affinities with MMP2 with a binding energy of −7.7 kcal/mol and −7.6 kcal/mol, respectively (Figure 5E,F), while sucrose was predicted to bind to ADRA2B and ADRA1A with a binding energy of −8.3 kcal/mol and −7.2 kcal/mol, respectively (Figure 5G,H). Taken together, increased UNII‐0TM46496W4, 12‐hydroxyeicosatetraenoic acid, Carnitine C12:1, and sucrose were identified as key microbial transducers associated with the detrimental effect of *Apiotrichum* on elevated arterial stiffness.

To further explore the functional link between *Apiotrichum* and the identified metabolite signatures, we performed secondary metabolite gene cluster prediction on the reference genome of a representative strain (*Apiotrichum porosum* DSM 27194) using antiSMASH. The analysis revealed multiple biosynthetic gene clusters (BGCs) relevant to our metabolomic findings (Figure S6). A lysine‐associated BGC (Region 2.1) was identified, and lysine is the established precursor for carnitine biosynthesis, supporting the detection of carnitine species such as Carnitine C12:1 in our cohort. Several saccharide BGCs (Regions 3.1, 3.2, etc.) were predicted, consistent with the potential for sucrose‐related metabolism. Additionally, a fatty acid BGC (Region 4.1) was detected, whose presence is compatible with the biosynthesis of oxygenated fatty acids like 12‐hydroxyeicosatetraenoic acid. These *in silico* findings provide orthogonal genomic support for the metabolic capacity of *Apiotrichum* to produce or contribute to the key mediator classes identified, thereby strengthening the biological plausibility of the observed associations.

## 4 Discussion

In this deeply phenotyped cohort of participants with varying levels of baPWV, we demonstrated, for the first time, that fungal co‐occurrence network altered with the deterioration of elevated arterial stiffness and preceded the changes in fungal compositions. Key fungal genera, including *Apiotrichum*, *Mycothermus*, *Flavocillium*, *Hyaloscypha*, and *Microscypha*, were potentially causally linked to increased baPWV levels. Moreover, through an integration of multi‐omics and *in silico* analysis, increased UNII‐0TM46496W4, 12‐hydroxyeicosatetraenoic acid, and sucrose were identified as molecular transducers linking *Apiotrichum* to elevated arterial stiffness, via the modulation of vascular vasoconstriction and smooth muscle cell function.

Imbalance in the gut microbiome has been recently identified as an emerging therapeutic target for several aging‐related disorders, including vascular aging (Brunt et al. 2019). Despite an integral part of the microbial community, the functional role of gut fungi in vascular health remains largely unknown. In contrast to an inverse association of bacteria diversity with arterial stiffness in middle‐aged women from the UK (Menni et al. 2018), no significant difference in fungal diversity was observed in subjects with normal or enhanced arterial stiffness. This observation was further supported by the insignificant difference in microbial diversity in medication naïve participants with normal and elevated arterial stiffness matched for age and sex (Luo et al. 2023), suggesting that the difference in diversity level might be biased by demographic characteristics. Moreover, as a complex ecological community, it is the interactions of microorganisms, rather than the compositions of the microbes alone, that determine the stability and metabolic capacities of the microbiome and drive the association with health outcomes (He et al. 2024). In support of this notion, we found that despite similar fungal taxon composition between the two groups, the fungal interaction networks were distinctly different, even after adjustment for age, sex, BMI, and medication use. This suggests that alterations in fungal interactions may precede compositional changes during the progression of arterial stiffness. Moreover, key parameters assessing the structure of the co‐occurrence network, such as betweenness and clustering coefficient, were closely associated with arterial stiffness and mean arterial pressure, indicating that gut fungal networks tended to be less clustered and more communicative in individuals with elevated arterial stiffness, while the symbiotic patterns in subjects with normal arterial stiffness were dominated by key fungal species with high clustering coefficients. Similar to the observation in this study, an increasingly complex interaction of fungal taxa was found with the deterioration of glycaemic control, without significant difference in composition (He et al. 2024), lending further support to the hypothesis that fungal co‐abundant networks may represent a novel target for the management of cardiometabolic disorders.

Moreover, given the long‐standing controversy about whether fungal changes promote cardiometabolic diseases or disturbance in metabolic homeostasis leads to alterations of gut mycobiome, bi‐directional MR analysis was further employed. Among the various fungal taxa driving the transformation of interaction networks with the exacerbation of arterial stiffness, enrichment of *Apiotrichum*, while depletion of *Mycothermus*, *Flavocillium*, *Hyaloscypha*, and *Microscypha* were found to be causally involved in the increase of arterial stiffness. Though increased abundance of *Apiotrichum* had been reported in patients with hypertension and chronic kidney disease (Qiu et al. 2023), which may accelerate arterial stiffness, neither SBP nor DBP could explain the adverse effect of *Apiotrichum* on elevated arterial stiffness. On the contrary, capable of producing xylooligosaccharides, a prebiotic reported to alleviate colonic inflammation caused by obesity (Fei et al. 2020), it was not surprising to find that increased *Mycothermus* might delay the progression of enhanced arterial stiffness. In a similar fashion, consistent with our observation that *Flavocillium* protected against elevated arterial stiffness, it was found to be a newly proposed genus of *Cordyceps* fungi, which was traditionally used in Chinese medicine for promoting metabolic health (Wang et al. 2020). Moreover, though reports about the role of *Hyaloscypha* and *Microscypha* in cardiometabolic health were limited, *Hyaloscypha* was found to be enriched in healthy infants following an exposure to perinatal antibiotics (Tejesvi et al. 2023), and increased *Microscypha* was identified as an independent predictor of favorable prognosis in patients with COVID‐19 (Lv et al. 2021; Farsi et al. 2022; Tandarto et al. 2024). Taken together, though further investigation is needed to clarify the exact mechanisms responsible for the distinct fungal interactions, we believe that targeted modulation of these key fungal species through lifestyle interventions, such as dietary changes, probiotics, prebiotics, and physical activity, may serve as an alternative strategy for the management of arterial stiffness.

In addition to the activation of immune responses (Wu et al. 2021), the fungal community may also exert its role through circulating metabolites, either via the production of secondary metabolites or through interaction with bacteria (Ost and Round 2023). Consistently, a significant association between intestinal fungi and plasma metabolites was observed in our cohort. Moreover, a cluster of metabolites, mainly from organic acids and fatty acids, was found to be the microbial effectors linking altered gut mycobiome to arterial stiffness. Consistent with the positive correlations between acylcarnitine species, particularly medium and long‐chain acylcarnitines, and PWV in the elderly (Koh et al. 2018), a cluster of acylcarnitine species was found to be crucial mediators linking the adverse effect of *Apiotrichum* on arterial stiffness, possibly through a disturbance in endoplasmic reticulum, peroxisome, and/or mitochondrial function. Moreover, several biomarkers of cardiovascular diseases, such as 1‐Methyluric acid, previously reported to be associated with congenital heart defect (Wang et al. 2023), 12‐hydroxyeicosatetraenoic acid, closely related to ischemic cardiac disease, maladaptive cardiac hypertrophy, and heart failure (Pascale et al. 2021), and phosphoenolpyruvate, capable of inducing endothelial dysfunction and cell senescence (An et al. 2023), mediated the deteriorative effect of *Apiotrichum* on elevated arterial stiffness. Additionally, several metabolites reported to be involved in inflammatory diseases and oxidative stress, such as 3‐Dehydrosphinganine (Qian et al. 2023), 8‐iso Prostaglandin F1alpha (Geraci et al. 2025), and 9,10,13‐TriHOME (Caligiuri et al. 2014), were found to be enriched in individuals with elevated arterial stiffness and accounted for approximately 73.79% of the detrimental effect of *Apiotrichum* on arterial stiffness. On the contrary, several amino acids which were reported to protect against cardiometabolic disorders, such as glutamine (Hasani et al. 2021), L‐Homoserine (Patel et al. 2020), and L‐Threonine (Petruhnova et al. 2024), were remarkably lower in individuals with elevated arterial stiffness and explained a large proportion of the favorable effect of *Microscypha* and *Mycothermus* on arterial stiffness. Notably, we identified fasting plasma sucrose as a key metabolite mediating the detrimental effect of *Apiotrichum* enrichment on arterial stiffness (Table S7). According to classical physiology, intact sucrose is not expected to circulate in fasting plasma because it is rapidly hydrolyzed by small intestinal sucrase. However, using high‐sensitivity GC–MS sugar profiling, Mack and colleagues demonstrated that more sugar compounds than anticipated, including sucrose, can be detected in fasting human plasma, with levels comparable to other monosaccharides (except glucose) (Mack et al. 2020). This technical evidence supports the feasibility of detecting fasting plasma sucrose using modern metabolomics platforms. Nevertheless, no published evidence suggests that *Apiotrichum* is capable of producing sucrose. Therefore, we hypothesize that excessive proliferation of *Apiotrichum*, as a core feature of fungal interaction network disturbance, may compromise intestinal barrier integrity, thereby facilitating the translocation of intact sucrose from the gut lumen into the systemic circulation. This interpretation aligns with previous work establishing sucrose as a permeability marker for gastroduodenal injury (Meddings et al. 1993). We acknowledge that direct evidence linking *Apiotrichum* to gut barrier dysfunction in the present cohort is lacking, and this speculative mechanism requires rigorous experimental validation in future studies before causal conclusions can be drawn.

Furthermore, several highly probable binding interactions between these important fungi‐related metabolites and targets implicated in the modulation of vascular functions had been found. For example, UNII‐0TM46496W4, an *Apiotrichum*‐related metabolite, served as a ligand for both CYP11B2 and ROCK2, with high affinity. As the crucial enzyme responsible for synthesizing aldosterone, the upregulation of CYP11B2 could promote the buildup of fibrous tissue in arteries and lead to structural stiffening (Lacolley et al. 2009). Beyond its role in fibrosis, aldosterone has been shown to impair endothelial function by reducing nitric oxide (NO) bioavailability through enhanced oxidative stress and endothelial NADPH oxidase activation, further compromising vascular relaxation (Maron et al. 2012). Moreover, ROCK2 was reported to be the key enzyme that drives arterial stiffness by regulating vascular smooth muscle cell contraction, actin polymerization, and fibrosis (Li et al. 2021). ROCK2 directly phosphorylates endothelial nitric oxide synthase (eNOS) at Thr495, suppressing NO production and promoting endothelial dysfunction (Vanhoutte et al. 2016). Similarly, MMP2, the key player in vascular remodelling (Yasmin et al. 2005) and a direct negative regulator of eNOS via its proteolytic cleavage (Nagareddy et al. 2012), demonstrated a strong binding affinity with both 12‐hydroxyeicosatetraenoic acid and carnitine C12:1. Though the upregulation of MMP2 by 12‐hydroxyeicosatetraenoic (Yang et al. 2019) acid and Carnitine C12:1 (Li et al. 2026) had been reported in both in vitro and in vivo studies, our findings suggest that it may serve as the molecular mechanism whereby increased *Apiotrichum* correlates with exacerbated arterial stiffness. Additionally, consistent with a previous report that activation of ADRA2B and ADRA1A leads to vasoconstriction and elevated blood pressure (Tikhonoff et al. 2008) while counteracting NO‐mediated endothelial relaxation (Belo Nunes et al. 2013), it was identified as a downstream effector of sucrose in the circulation. Collectively, though we could not differentiate the origin of these metabolites at this stage, and more investigation in more diverse populations are warranted, our integrative analysis suggested that alterations of gut mycobiome are associated with the progression of arterial stiffness via organic acids and fatty acyls.

In addition, our cohort is characterized by a substantially higher prevalence of antihypertensive drug use in the elevated arterial stiffness group. (24.71% vs. 7.54%, *p* < 0.001; Table 1), making them a potentially important confounder in the association between gut mycobiome and arterial stiffness. Although direct evidence linking these medications to the gut mycobiome remains limited, several lines of research support the biological plausibility of such confounding. For instance, losartan can increase the abundance of beneficial bacteria such as *Lactobacillus*, and diltiazem can be directly metabolized by the gut commensal *Bacteroides thetaiotaomicron* (Xiong et al. 2021). More directly, the antihypertensive agent aliskiren has been shown to possess intrinsic antifungal activity against *Candida albicans* , impairing its growth, biofilm formation, and adhesion (Kathwate and Karuppayil 2013). Thus, antihypertensive drugs may influence the gut mycobiome through both indirect (bacteria‐fungi cross‐talk) and direct (antifungal) pathways. To rigorously address this confounding concern, we performed propensity score matching (PSM) that additionally accounted for medication usage (Table S2). In this medication‐matched sub‐cohort, the distinct topological properties of the fungal co‐occurrence network remained robust compared with the original cohort (Figure 1I–L), and the key genera retained their significant associations with arterial stiffness (Figure 2A). Moreover, our bidirectional MR analyses, which are not susceptible to confounding by medication usage, independently supported a causal role for these fungal genera (Figure 2D–I). Collectively, these results suggest that antihypertensive drugs are unlikely to be a major factor of our primary findings, although residual confounding cannot be entirely excluded.

Despite an extensive adjustment and several sensitivity analyses, several limitations of this study should be acknowledged. First, our participants were exclusively Chinese recruited from a single center, and thus further validation in larger and more diverse ethnic groups is warranted. Second, this study was cross‐sectional, limiting its ability to establish causal inferences. However, we employed bi‐directional MR analysis to investigate the potential causal involvement of fungal genera in the modulation of arterial stiffness. Third, although integrative analysis and *in silico* analysis improved the biological understanding of fungi in vascular health, further validation in animal models is warranted.

In summary, our study uncovers a strong association between disturbance in gut fungi and the deterioration of arterial stiffness and identifies several organic acids and amino acids as potential molecular transducers to the modulatory effect of important fungi on arterial stiffness. Specifically, we highlight that several *Apiotrichum*‐related metabolites may contribute to the modulation of arterial stiffness via multiple downstream targets. This finding highlights the potential of targeting the gut mycobiome for improving vascular health.

## Author Contributions

**Jiarui Chen:** writing – review and editing, writing – original draft, visualization, validation, formal analysis, investigation. **Jialin He:** methodology, software, writing – review and editing. **Bingqi Ye:** methodology, software, writing – review and editing. **Jialu Yang:** resources, data curation, writing – review and editing. **Ludi Liu:** resources, data curation, writing – review and editing. **Jingmeng Ju:** resources, writing – review and editing. **Sinan Li:** writing – review and editing, visualization. **Benjie Li:** writing – review and editing, visualization. **Min Xia:** conceptualization, writing – review and editing, supervision, project administration. **Yan Liu:** conceptualization, writing – review and editing, supervision, project administration, funding acquisition. All authors reviewed the manuscript.

## Funding

This work was supported by National Key Research and Development Program of China (2023YFC3606300), Joint Funds of the National Natural Science Foundation of China (No. U24A20769), Young Scientists Fund of the National Natural Science Foundation of China (No. 82404256), the National Natural Science Foundation of China (No. 82273611), and Fundamental Research Funds for the Central University, Sun Yat‐sen University (2025QNPY01).

## Ethics Statement

This study was approved by the Ethics Committee of the School of Public Health at Sun Yat‐sen University (2017‐001), and was in accordance with the principles of the Declaration of Helsinki. Written informed consent was obtained from each participant.

## Consent

The authors have nothing to report.

## Conflicts of Interest

The authors declare no conflicts of interest.

## Supporting information


**Figure S1:** Gut Fungal Community in Subjects with Normal and Elevated Arterial Stiffness. (A) Stacked bar plots showing the taxonomic composition at the phylum level for each individual (*n* = 504 and *n* = 259 for normal and elevated arterial stiffness groups, respectively). (B) Phylogenetic tree of the top 40 most abundant fungal genera, constructed from ITS1 sequencing data. Colors in (A) and (B) represent phylum‐level annotation. (C) Box plot comparing the Shannon diversity index between groups. No significant difference was detected using logistic regression by adjusting sex, age, and BMI (*p* = 0.83). (D) Principal coordinate analysis (PCoA) plot based on Bray–Curtis dissimilarity, illustrating beta diversity between groups. PERMANOVA was used to assesss compositional. In panels (C) and (D), blue and red denote the normal and elevated arterial stiffness groups, respectively.
**Figure S2:** Distribution of network properties in individuals with normal and elevated arterial stiffness. Density plots showing the distribution of four network properties including node degree, betweenness centrality, closeness centrality, and clustering coefficient in the fungal interaction network of subjects with normal and elevated stiffness in the (A) whole cohort, (B) sub‐cohort 1, and (C) sub‐cohort 2, respectively. Blue and red indicated normal and elevated arterial stiffness, respectively.
**Figure S3:** Leave‐one‐out plots for MR results. Each row represented the SNP‐exposure effect size with corresponding SE. The line with yellow diamond represented the average effect of all SNPs as calculated by the inverse variance weighted method.
**Figure S4:** Reverse MR analysis. Scatterplot of associations between genetic variant closely related to arterial stiffness and key fungi taxa, including (A) Apiotrichum, (B) Mycothermus, (C) Flavocillium, (D) Hyaloscypha, and (E) Microscypha. The slope of each line corresponded to the estimated MR effect.
**Figure S5:** Association of SCFAs with arterial stiffness, cardiovascular parameters, and key fungal genera. (A and B) Bar charts showing the significant associations of SCFAs with binary baPWV (A) and continuous baPWV (B). Red bars indicate positive associations and blue bars indicate negative associations. (C) Heatmap displaying Spearman correlation coefficients between the identified SCFAs and multiple cardiovascular parameters (left panel) as well as with significant fungal genera (right panel). Red indicates positive correlation, and blue indicates negative correlation. Cells with *p*_*FDR*_ < 0.05 are marked with solid circles. Cells with *p*_*FDR*_ < 0.2 are marked with hollow circles. Non‐significant cells are shaded in gray.
**Figure S6:** Identified secondary metabolite regions of representative *Apiotrichum* strain (*Apiotrichum porosum* DSM 27194) genome. Gene structure diagrams of different regions in the genome of *Apiotrichum porosum* DSM 27194. Different colors indicate distinct annotation types.
**Table S1:** Basic Characteristics of the study participants matched for sex, age, and BMI (subchort 1).
**Table S2:** Basic Characteristics of the study participants matched for age, sex, BMI and medication (subcohort 2).
**Table S3:** Partial Spearman correlation between network topology and cardiovascular parameters.
**Table S4:** Summary statistics of genetic instruments used in this study.
**Table S5:** Forward MR results.
**Table S6:** Backward MR results.
**Table S7:** Mediation analysis results.
**Table S8:** Downstream target analysis.
**Table S9:** Top 50 related genes with arterial stiffness retreived from NCBI database.
**Table S10:** Top 50 related genes with PWV retreived from GeneCards database.
**Table S11:** Molecular docking results.

**Data S1:** acel70650‐sup‐0002‐Supinfo1.docx.

## Data Availability

ITS sequencing data from this study has been deposited at the NCBI Sequence Read Archive under BioProject: PRJNA1273442. The metabolomics raw data have been deposited in the MetaboLights database under accession number REQ20260522219902. This study doesn't generate any new codes. Any additional information required to reanalyze the data reported in this paper is available from the lead contact upon reasonable request.
