# Supplementary material for: Apiotrichum‐Centered Fungal Interaction Network Disturbance Correlates With Increased Arterial Stiffness
Source: Aging Cell. 2026 Jul 25;25(8):e70650. doi: 10.1111/acel.70650 (PMC13401687; doi:10.1111/acel.70650)
Supplement: Supplementary file 1 — Figure S1: Gut Fungal Community in Subjects with Normal and Elevated Arterial Stiffness. (A) Stacked bar plots showing the taxonomic composition at the phylum level for each individual (n = 504 and n = 259 for normal and elevated arterial stiffness groups, respectively). (B) Phylogenetic tree of the top 40 most abundant fungal genera, constructed from ITS1 sequencing data. Colors in (A) and (B) represent phylum‐level annotation. (C) Box plot comparing the Shannon diversity index between groups. No significant difference was detected using logistic regression by adjusting sex, age, and BMI (p = 0.83). (D) Principal coordinate analysis (PCoA) plot based on Bray–Curtis dissimilarity, illustrating beta diversity between groups. PERMANOVA was used to assesss compositional. In panels (C) and (D), blue and red denote the normal and elevated arterial stiffness groups, respectively. Figure S2: Distribution of network properties in individuals with normal and elevated arterial stiffness. Density plots showing the distribution of four network properties including node degree, betweenness centrality, closeness centrality, and clustering coefficient in the fungal interaction network of subjects with normal and elevated stiffness in the (A) whole cohort, (B) sub‐cohort 1, and (C) sub‐cohort 2, respectively. Blue and red indicated normal and elevated arterial stiffness, respectively. Figure S3: Leave‐one‐out plots for MR results. Each row represented the SNP‐exposure effect size with corresponding SE. The line with yellow diamond represented the average effect of all SNPs as calculated by the inverse variance weighted method. Figure S4: Reverse MR analysis. Scatterplot of associations between genetic variant closely related to arterial stiffness and key fungi taxa, including (A) Apiotrichum, (B) Mycothermus, (C) Flavocillium, (D) Hyaloscypha, and (E) Microscypha. The slope of each line corresponded to the estimated MR effect. Figure S5: Association of SCFAs with arterial stiffness [file ACEL-25-e70650-s002.docx]

**SUPPLEMENTAL MATERIAL TO**

***Apiotrichum*-centered fungal interaction network disturbance correlates with increased arterial stiffness**

Jiarui Chen^1^, Jialin He^1^, Bingqi Ye^2^, Jialu Yang^1^, Ludi Liu^2^, Jingmeng Ju^1^, Sinan Li^1^, Benjie Li^1^, Min Xia^1^, Yan Liu^1,*^

^1^Department of Nutrition, School of Public Health, Sun Yat-sen University, Guangzhou, P.R. China

^2^Department of Statistics and Epidemiology, School of Public Health, Sun Yat-sen University, Guangzhou, P.R. China

Correspondence to: Prof. Yan Liu ([liuyan215@mail.sysu.edu.cn](mailto:liuyan215@mail.sysu.edu.cn)).

**This file includes:**

1. **Supplemental Methods**
2. **Figures S1-S6**
3. **Table S1-S11**

**Supplemental Methods**

**Metabolomics profiling in study participants**

A novel integrated method for large-scale detection, identification, and quantification of metabolites^62^ was employed for metabolomics profiling in study participants. Briefly, 50 μL of each plasma sample was thawed on ice, vortexed for 10 sec, followed by the addition of 300 μL of pure methanol with internal standards, including L-2-chlorophenylalanine, [^2^H_3_]-L-carnitine HCl, 4-fluoro-L-α-phenylglycine, L-phenylalanine (2-^13^C, 99%), [^2^H_5_]-hippuric acid, [^2^H_5_]-kynurenic acid, [^2^H_5_]-phenoxy acetic acid^62^. The mixture of plasma and internal standards was vortexed for 3 min, and the supernatants were recovered by centrifugation at 12,000 rpm for 10 min at 4°C. Then, 10 μL of the supernatant taken from each sample was pooled together to create a “mixed sample”, followed by a full scan mode with QTRAP^®^ 6500+ system to acquire a high-resolution data including RT, Q_1_, and Q_3_. Peak annotation of the mixed sample was performed based on the orthogonal combination of RT, precursor/product ion pairs (Q1/Q3), and MS/MS spectral information matched against the *in house* database of Metware Co., Ltd (Wuhan, China). Compounds with identical molecular formulas but distinct Q1 channels, different RT windows, or non-overlapping MS/MS fragmentation patterns were processed as independent chromatographic features. This multi-dimensional identification strategy reduces the risk of misassignment between isomeric or isobaric metabolites. The ion pair information was then transferred to the triple quadrupole (QQQ) instrument. Finally, individual plasma samples were analyzed by QQQ in multiple reaction monitoring (MRM) mode to get a more accurate quantification of metabolites. More details for this process and instrument setup were as follows: chromatographic separation was performed on ACQUITY UPLC HSS T3 C18 (1.8 µm, 2.1 mm×100 mm, Waters) using a UPLC system (Shim-pack UFLC SHI-MADZU CBM A system, https://www.shimadzu.com/; QTRAP^®^ 6500+ System, https://sciex.com/). Mass spectrometry (MS) detection was performed by triple quadrupole-linear ion trap mass spectrometry (QTRAP), equipped with an electrospray ionization (ESI) source. Full scans with a range of m/z 50-1000 were acquired in LIT and QQQ scans with positive and negative ion modes and controlled by Analyst 1.6.3 software (Sciex). The ESI source parameters were as follows: source temperature 500°C; ion spray voltage (IS) 5500 V (positive), -4500 V (negative); ion source gas I (GSI), gas II (GSII), curtain gas (CUR) were set at 55, 60, and 25.0 psi, respectively; the collision gas (CAD) was high. Instrument tuning and mass calibration were performed with 10 and 100 μmol/L polypropylene glycol solutions in QQQ and LIT modes, respectively. A specific set of MRM transitions was monitored for each period according to the metabolites eluted within this period.

**Multi-omics analyses**

***Quality control, taxonomy profiling and annotation for ITS data***

The PIPITS pipeline (version 2.8)^63^ was utilized for ITS sequencing data analysis, employing default parameters that included quality filtering, read-pair merging, and ITS1 filtering. This pipeline used the FASTX-Toolkit for quality filtering, the removal of short sequences (length < 100 bp) and singletons, and it extracted the ITS1 region sequence using ITSx. Remaining reads were binned into operational taxonomic units (OTUs) based on 97% similarity, and chimeras were removed using VSEARCH (version 2.17.1). Individual OTUs were taxonomically classified based on the UNITE (Version 9.0) database with the SINTAX algorithm^64^. For downstream analysis, all samples were normalized using cumulative sum scaling via MetagenomeSeq (version 1.40.0)^65^.

Alpha diversity, measured by the Shannon index, was calculated for each sample at the genus level using the vegan package (version 2.6-4)^66^. Logistic regression was implemented to compare the difference of alpha diversity between the two groups with the adjustment for age, sex, and BMI. Beta diversity was assessed using the Bray-Curtis distance matrix, and a permutational multivariate analysis of variance (PERMANOVA) was conducted to evaluate the relationship between fungal composition and arterial stiffness, adjusting for age, sex, and BMI, with 999 permutations.

***Co-abundant network of fungi***

To investigate the ecological interactions among fungal genera, co-abundant networks were constructed in all fungal genera detected with no prevalence filtering using pairwise Spearman correlation analyses within subjects categorized by arterial stiffness status. Only significant correlations (*P*_adj_ < 0.05) with an absolute coefficient value (|rho|) greater than 0.3 were included in the network construction, and were visualized with Gephi version 0.10. Network topological parameters, including node, edge, average degree, average path length, and cluster coefficient, were calculated utilizing the igraph package (version 1.4.3).

The significance of each pair of fungal genera was compared between the networks of subjects with normal or elevated arterial stiffness. These pairs were categorized into three groups: (1) pairs significant only in subjects with normal arterial stiffness, (2) pairs significant only in individuals with elevated arterial stiffness, and (3) pairs significant in both networks. The counts of these three types of pairs were recorded and visualized using a Venn diagram. For each network, node attributes, including degree, betweenness centrality, closeness centrality, and clustering coefficient, were computed using a bootstrapping approach with 10,000 iterations. Differences in network attributes were assessed with a two-sample Kolmogorov-Smirnov test. The associations between topological features and clinical parameters indicative of arterial stiffness were determined via partial Spearman correlation analysis with the adjustment for age, sex, and BMI.

Furthermore, to mitigate the potential impact of unbalanced sample sizes and confounding factors such as age, sex, and BMI on the fungi networks, participants with and without elevated arterial stiffness were matched using propensity scores derived from a logistic model with bias correction. This matching (sub-cohort 1) employed a 1:1 nearest-neighbor matching approach, restricting the caliper width for propensity matching to 0.05 to ensure optimal pair matching. A total of 408 individuals were successfully matched, with 204 participants from each group. Moreover, to further account for the potential effects of medication usage on gut fungi, individuals were matched based on propensity scores from a logistic model that included age, sex, BMI, and the use of three types of medications: anti-hypertensive, anti-diabetic, and hypolipidemic agents (sub-cohort 2). This resulted in 400 successful matches, with 200 participants in each group. All analyses related to the networks were repeated in this propensity-matched subgroup to verify changes in ecological connections among fungal taxa associated with elevated arterial stiffness.

***Key fungal genera and their association with arterial stiffness***

The NetMoss score^67^ was calculated for each node to evaluate its significance in the network transition between the two groups. This score was compared to its null distribution, generated through a permutation test with 100 replications, where networks were reconstructed by randomly reshuffling sample labels. Comparisons with the null model were assessed using one-sample t-test, with a significance level of *P*_adj_ < 0.05. Only fungal genera that showed statistical significance in the whole cohort, as well as both propensity-matched sub-cohorts were regarded as network key features.

Moreover, the associations between the fungal genera identified as network key features and binary baPWV (normal controls with baPWV < 1400 cm/s and elevated arterial stiffness with baPWV ≥ 1400 cm/s), or contentious baPWV were assessed using generalized linear models in MaAsLin2^68^, employing default parameters. Age, sex, BMI, and medication usage were adjusted as confounders. Only fungal genera significantly associated with elevated arterial stiffness or baPWV (*P*_adj_ < 0.2) were regarded as key fungal genera and subjected to the subsequent MR analysis.

***SNPs selection and bi-directional Mendelian randomization analysis***

A bi-directional one-sample MR analysis was employed to assess the causal relationships between key fungal genera and arterial stiffness. Quality control was conducted with PLINK (v.1.9), and single-nucleotide polymorphisms (SNPs) were excluded based on the following criteria: (1) Minor Allele Frequency < 5%; (2) Hardy-Weinberg equilibrium violation with *P* < 0.00001, and (3) genotype calling rate < 5%^69^. Additionally, linkage disequilibrium (LD) was calculated for each pair of SNPs over a window of 50 SNPs, with one SNP of any pair removed if the LD exceeded 0.5. Ultimately, a total of 399,453 SNPs were retained for the current genome-wide association study involving 752 participants. The threshold of *P* < 5×10^-5^ was set for identifying mycobiome-associated SNPs to maximize the proportion of genetic variance explained by the selected SNPs, as previously described^69^.

Bi-directional MR analysis was conducted using the MendelianRandomization^70^ (version 0.9.0). SNPs with an F statistic (beta^2^/SE^2^)>10 were considered as strong genetic instrumental variables (IVs) and were utilized for subsequent MR analysis. MR estimates were calculated using IVW methods with a random effects model. In addition, we reported MR estimates using the weighted median and MR-Egger regression methods. To ensure the validity of the results, potential causal estimates were selected based on the following criteria: (1) *P* < 0.05 calculated by the IVW method; (2) consistent direction in effect sizes across the three methods; and (3) *P*>0.05 for the intercept term calculated by the MR-Egger method, indicating no pleiotropic effects. SNP annotation was performed using VarNote^71^ to obtain positional information, variant effects and nearest neighbour genes of the SNPs. Moreover, to identify potential heterogeneous SNPs, a “leave-one-out” analysis was performed by omitting each instrumental SNP in turn. Furthermore, reverse MR analysis on selected important fungal genera was performed by the same procedure to ensure causality.

***Mediating effect of blood pressure from fungal genera to arterial stiffness***

To further explore the mediating effect of blood pressure on the causal relationships between key fungal genera and arterial stiffness (measured as either binary or continous baPWV), mediation analysis was conducted using the mediate package (version 4.0). The total effect, direct effect, and indirect effect of both SBP and DBP from the key fungal genera on arterial stiffness were estimated. The results were validated through a simulation exercise bootstrapped 1000 times.

***Differential metabolites and their association with phenotypes and key fungal taxa***

For the metabolomics data, all samples were normalized using total sum scaling prior to downstream analyses. Procrustes analysis was firstly conducted to investigate the overall relationship between fungal genera and circulating metabolites, with *P* value generated through 999 permutations. Furthermore, to identify metabolites independently associated with binary baPWV (normal controls with baPWV < 1400 cm/s and elevated arterial stiffness with baPWV ≥ 1400 cm/s) or contentious baPWV, generalized linear models in MaAsLin2^68^ was employed with default parameters. Age, sex, BMI and medication usage were adjusted as confounders. Only metabolites significantly associated with binary or contentious baPWV (*P*_adj_ < 0.2) were regarded as important and subjected to further analyses. Spearman correlation analysis was performed to evaluate the associations between the identified SCFAs and multiple cardiovascular parameters, as well as the associations between key fungal genera and significant metabolites. Moreover, mediation analysis was performed to explore whether selected metabolites mediate the effect of key fungi on arterial stiffness. The results were validated through simulation exercises bootstrapped 1000 times, and visualized using Sankey diagrams generated with the R package networkD3 (version 0.4).

***In silico analysis for downstream targets of selected metabolites***

The metabolites with significant mediating effects of key fungi on arterial stiffness were subjected to downstream targets prediction (probability > 0.1 as the cut-off) by SwissTargetPrediction (<http://www.swisstargetprediction.ch/>). Furthermore, GO enrichment analysis was performed with all these potential downstream targets as input gene sets using clusterProfiler. The simplifyEnrichment package was then employed to cluster the similarity matrices of the enriched terms into groups using "binary cuts" to explore the main biological information contained in the results of the enrichment analysis.

To identify genes previously associated with arterial stiffness, we searched two public databases on August 29, 2024. In the NCBI Gene database (https://www.ncbi.nlm.nih.gov/gene), we used the search term "arterial stiffness" without additional filters and retrieved all human genes from the search results. In GeneCards (https://www.genecards.org), we used the search term "PWV" and included all genes returned by the default search mode. No further filtering was applied to ensure maximal inclusivity. The top 50 list of genes retrieved from NCBI Gene and GeneCards is provided in **Supplementary Tables 9** **and** **10**, respectively.

Furthermore, to evaluate the binding affinities and modes of interactions between selected metabolites and their downstream targets, AutodockVina 1.2.2, an *in silico* protein-ligand docking software, was employed^72^. Only targets that overlap in both the top 10 pathways related to cardiac functions and those previously reported to be involved in arterial stiffness in NCBI and GeneCards database were subjected to further analysis. The molecular structures of the metabolites were retrieved from PubChem Compound (https://pubchem.ncbi.nlm.nih.gov/)^73^. The 3D coordinates of the selected targets (**Supplementary Table 11**) were downloaded from the PDB (<https://www.rcsb.org/pdb/home/home.do>). For docking analysis, all protein and metabolites files were converted into PDBQT format with all water molecules excluded and polar hydrogen atoms added. The grid box was set to 30 Å x 30 Å x 30 Å, and grid point distance was 0.05 nm. Molecular docking was performed by AutodockVina 1.2.2 (<https://autodock.scripps.edu>). Only a binding energy of less than -7 kcal/mol, which was indicative of a highly stable binding, was considered as reliable and shown in the figure.

Moreover, to conduct the secondary metabolite gene cluster prediction for *Apiotrichum*, the genome sequence and annotation of the representative *Apiotrichum* strain (*Apiotrichum porosum* DSM 27194) were retrieved from the NCBI Genome database. Secondary metabolite biosynthetic gene clusters (BGCs) were predicted using antiSMASH^74^ (version 8.0.4) with the detection strictness set to “loose”. All other parameters were kept as default. The resulting BGCs were manually inspected for relevance to the identified metabolite classes. The output is presented in **Supplementary Figure 6**.

**Supplementary Figures**

**Supplementary Figure 1. Gut Fungal Community in Subjects with Normal and Elevated Arterial Stiffness**. (A) Stacked bar plots showing the taxonomic composition at the phylum level for each individual (n = 504 and n = 259 for normal and elevated arterial stiffness groups, respectively). (B) Phylogenetic tree of the top 40 most abundant fungal genera, constructed from ITS1 sequencing data. Colors in (A) and (B) represent phylum-level annotation. (C) Box plot comparing the Shannon diversity index between groups. No significant difference was detected using logistic regression by adjusting sex, age, and BMI (*P* = 0.83). (D) Principal coordinate analysis (PCoA) plot based on Bray-Curtis dissimilarity, illustrating beta diversity between groups. PERMANOVA was used to assesss compositional. In panels (C) and (D), blue and red denote the normal and elevated arterial stiffness groups, respectively

**
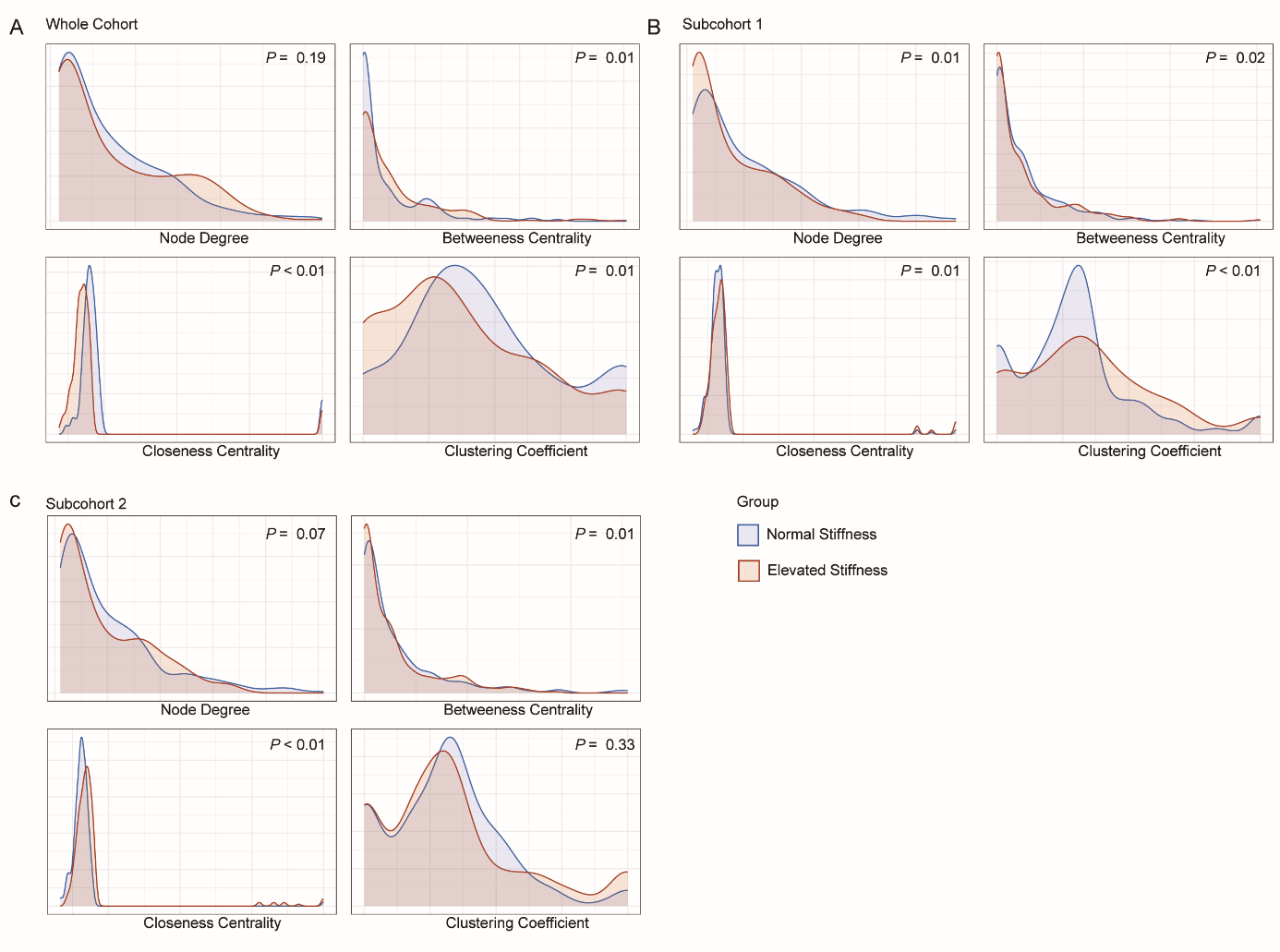
**

**Supplementary Figure 2. Distribution of network properties in individuals with normal and elevated arterial stiffness.** Density plots showing the distribution of four network properties including node degree, betweenness centrality, closeness centrality, and clustering coefficient in the fungal interaction network of subjects with normal and elevated stiffness in the (A) whole cohort, (B) sub-cohort 1, and (C) sub-cohort 2, respectively. Blue and red indicated normal and elevated arterial stiffness, respectively.

**
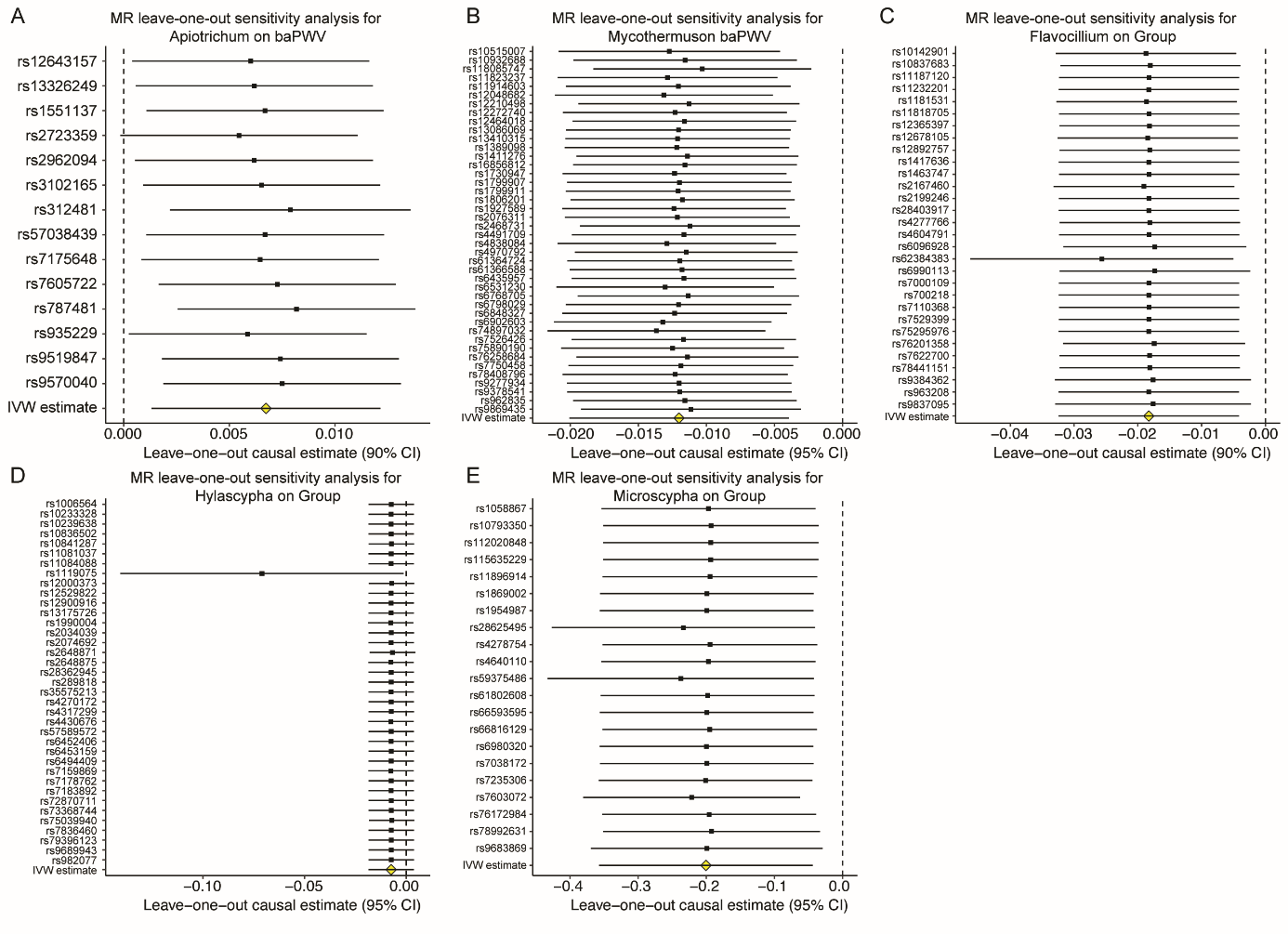
**

**Supplementary Figure 3. Leave-one-out plots for MR results.** Each row represented the SNP-exposure effect size with corresponding SE. The line with yellow diamond represented the average effect of all SNPs as calculated by the inverse variance weighted method.

**
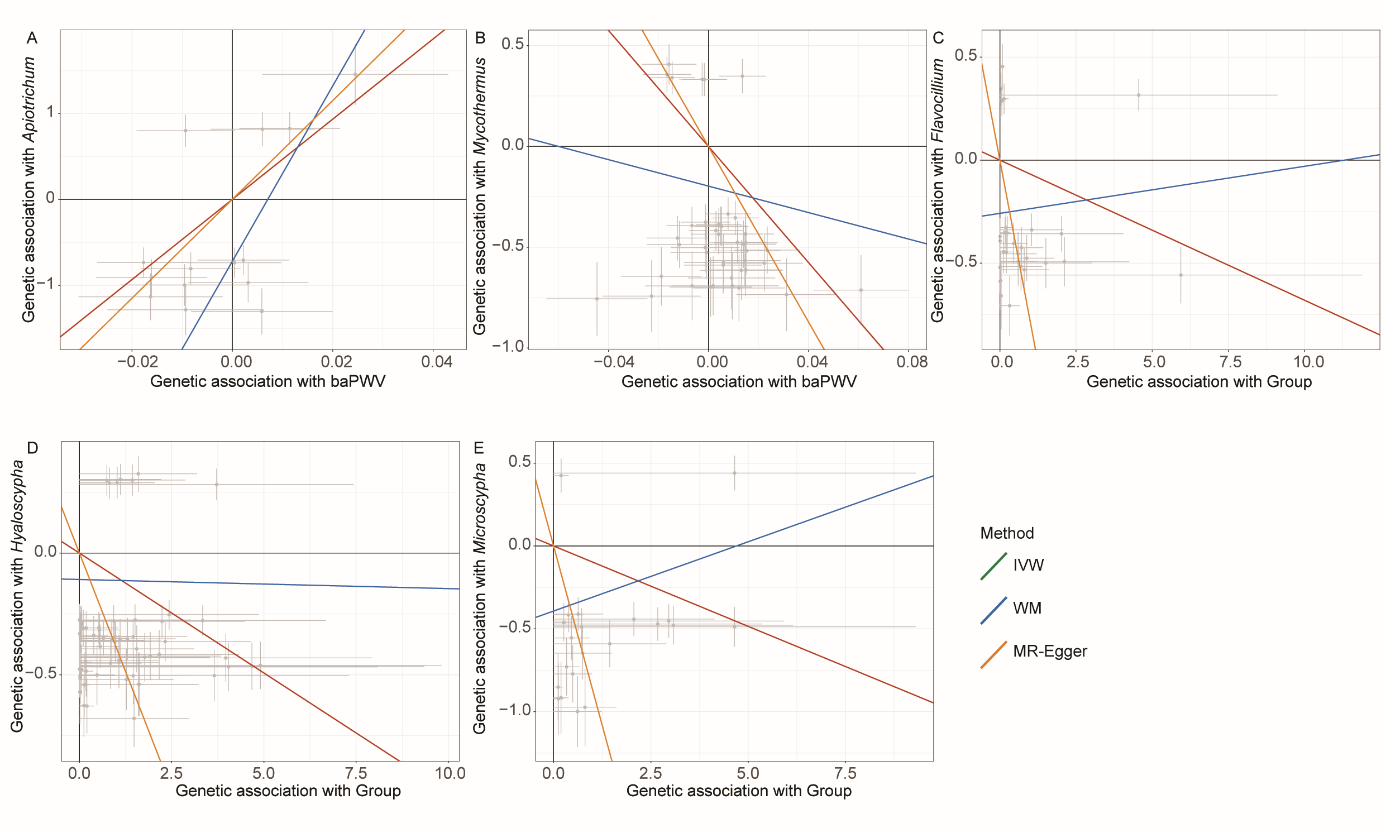
**

**Supplementary Figure 4. Reverse MR analysis.** Scatterplot of associations between genetic variant closely related to arterial stiffness and key fungi taxa, including (A) *Apiotrichum*, (B) *Mycothermus*, (C) *Flavocillium*, (D) *Hyaloscypha*, and (E) *Microscypha*. The slope of each line corresponded to the estimated MR effect.

**Supplementary Figure 5. Association of SCFAs with arterial stiffness, cardiovascular parameters, and key fungal genera.** (**A and B**) Bar charts showing the significant associations of SCFAs with binary baPWV (**A**) and continuous baPWV (**B**). Red bars indicate positive associations and blue bars indicate negative associations. (**C**) Heatmap displaying Spearman correlation coefficients between the identified SCFAs and multiple cardiovascular parameters (left panel) as well as with significant fungal genera (right panel). Red indicates positive correlation, and blue indicates negative correlation. Cells with *P_FDR_* < 0.05 are marked with solid circles. Cells with *P_FDR_* < 0.2 are marked with hollow circles. Non‑significant cells are shaded in gray.

**Supplementary Figure 6. Identified secondary metabolite regions of representative *Apiotrichum* strain (*Apiotrichum* *porosum* DSM 27194) genome.** Gene structure diagrams of different regions in the genome of *Apiotrichum porosum* DSM 27194. Different colors indicate distinct annotation types.

**Table S1 Basic Characteristics of the study participants matched for sex, age, and BMI (subchort 1)**

| **Characteristic** | **Total** | **Normal Arterial Stiffness** | **Elevated Arterial Stiffness** | ***P* value** | |
| --- | --- | --- | --- | --- | --- |
|  | **N = 408** | **N = 204** | **N = 204** |  |  |
| **Clinical characteristics** | | | | |  |
| Male, n (%) | 225 (29.49%) | 110 (21.83%) | 115 (44.4%) | 0.691 | |
| Age, years | 54 [48.75, 58] | 54 [49, 58] | 52.5 [48, 57] | 0.941 | |
| BMI, kg/m^2^ | 24.2 [22.1, 26.3] | 24.4 [21.8, 26.3] | 24.15 [22.45, 26.2] | 0.912 | |
| Dietary Diversity | 5 [5, 6] | 5 [5, 6] | 5 [5, 6] | 0.946 | |
| **Vascular function measurements** | | | | |  |
| RbaPWV, cm/s | 1403.5 [1250.75, 1566.25] | 1250.5 [1155.75, 1325] | 1566.5 [1483.75, 1675] | <0.001 | |
| LbaPWV, cm/s | 1390 [1239, 1551.25] | 1239 [1147.75, 1321.75] | 1551.5 [1468, 1675.5] | <0.001 | |
| baPWV, cm/s | 1400.25 [1241, 1556.75] | 1241 [1160.75, 1325.75] | 1557.5 [1479.62, 1672.25] | <0.001 | |
| RABI | 1.11 [1.06, 1.15] | 1.11 [1.06, 1.14] | 1.12 [1.07, 1.16] | 0.11 | |
| LABI | 1.11 [106, 1.14] | 1.10 [1.06, 1.14] | 1.11 [1.06, 1.15] | 0.105 | |
| ABI | 1.11 [107, 1.15] | 1.10 [1.07, 11.14] | 1.12 [1.07, 1.16] | 0.084 | |
| RbaMAP, mmHg | 97.10 [89.00, 105.48] | 89.95 [83.78, 95.94] | 104.28 [98.05, 112.0.6] | <0.001 | |
| LbaMAP, mmHg | 96.83 [87.89, 105.21] | 89.05 [83.15, 95.54] | 103.63 [98.00, 111.79] | <0.001 | |
| baMAP, mmHg | 97.26 [88.25, 105.53] | 89.28 [83.72, 95.51] | 103.86 [98.66, 111.82] | <0.001 | |
| plaque, n (%) | 0 [0, 1] | 0 [0, 0] | 0 [0, 1] | <0.001 | |
| IMT, mm | 0.73 [0.6, 1] | 0.7 [0.6, 1] | 0.8 [0.6, 1.1] | 0.153 | |
| China-PAR | 2.66 [1.47, 4.33] | 1.97 [1.13, 3.33] | 3.32 [1.88, 5.8] | <0.001 | |
| **Laboratory measurements** | | | | |  |
| SBP, mmHg | 127 [116.88, 137.5] | 119.25 [111.5, 127] | 134.5 [126.38, 146.5] | <0.001 | |
| DBP, mmHg | 81.5 [75, 88] | 77 [72.5, 83.5] | 85.75 [79.38, 93.25] | <0.001 | |
| PP, mmHg | 44.00 [38.88, 52.00] | 41.50 [36.00, 46.50] | 49.25 [42.50, 56.00] | <0.001 | |
| FG, mmol/L | 4.83 [4.36, 5.33] | 4.7 [4.26, 5.24] | 4.97 [4.5, 5.43] | 0.002 | |
| TG, mmol/L | 1.22 [0.86, 1.84] | 1.19 [0.81, 1.68] | 1.3 [0.91, 2] | 0.004 | |
| TC, mmol/L | 5.48 [4.94, 6.1] | 5.42 [4.94, 6.06] | 5.52 [4.94, 6.19] | 0.442 | |
| HDL-c, mmol/L | 1.37 [1.17, 1.61] | 1.39 [1.2, 1.63] | 1.35 [1.12, 1.59] | 0.071 | |
| LDL-c, mmol/L | 3.26 [2.86, 3.79] | 3.25 [2.87, 3.82] | 3.26 [2.86, 3.75] | 0.805 | |
| ALP, IU/L | 86.25 [73.65, 100.35] | 82.55 [73.12, 97.35] | 89.3 [75.7, 103.32] | 0.048 | |
| CREA, umol/L | 79.2 [68.3, 90.62] | 81.1 [69.2, 91.08] | 79.05 [68.1, 89.93] | 0.715 | |
| **Medication Usage** | | | | |  |
| Anti-hypertensive drugs, n (%) | 65 (8.52%) | 23 (4.56%) | 42 (16.22%) | 0.015 | |
| Anti-diabetic drugs, n (%) | 23 (3.01%) | 8 (1.59%) | 15 (5.79%) | 0.198 | |
| Hypolipidemic agents, n (%) | 16 (2.1%) | 7 (1.39%) | 9 (3.47%) | 0.799 | |

Data were shwon as median (interquartile range) or number (percentage). *P* value represented the difference between groups in percent (%) or median (IQR).

**Table S2 Basic Characteristics of the study participants matched for age, sex, BMI and medication (subcohort 2)**

| **Characteristic** | **Total** | **Normal Arterial Stiffness** | **Elevated Arterial Stiffness** | ***P* value** | |
| --- | --- | --- | --- | --- | --- |
|  | **N = 400** | **N = 200** | **N = 200** |  |  |
| **Clinical characteristics** | | | | |  |
| Male, n (%) | 218 (28.57%) | 107 (21.23%) | 111 (42.86%) | 0.763 | |
| Age, years | 53 [48, 58] | 54 [48, 58] | 52 [48, 58] | 0.844 | |
| BMI, kg/m^2^ | 24.2 [22.2, 26.2] | 24.3 [21.98, 26.3] | 24.2 [22.67, 26.2] | 0.903 | |
| Dietary Diversity | 5 [5, 6] | 5 [4.71, 6] | 5 [5, 6] | 0.382 | |
| **Vascular function measurements** | | | | |  |
| RbaPWV, cm/s | 1405 [1263.25, 1560.75] | 1261.5 [1170.5, 1325] | 1561.5 [1476.5, 1671.25] | <0.001 | |
| LbaPWV, cm/s | 1401 [1248.75, 1542.25] | 1248.5 [1165, 1328] | 1542.5 [1467.75, 1653.5] | <0.001 | |
| baPWV, cm/s | 1400.25 [1257.5, 1545.88] | 1257 [1167.25, 1326.75] | 1546.25 [1474.38, 1658.25] | <0.001 | |
| RABI | 1.11 [1.06, 1.15] | 1.11 [1.06, 1.15] | 1.12 [1.06, 1.16] | 0.335 | |
| LABI | 1.11 [1.06, 1.15] | 1.10 [1.07, 1.14] | 1.11 [106, 1.15] | 0.551 | |
| ABI | 1.11 [1.07, 1.15] | 1.11 [1.07, 1.14] | 1.12 [107, 1.15] | 0.4 | |
| RbaMAP, mmHg | 97.65 [88.54, 106.65] | 89.58 [83.19, 95.95] | 104.40 [97.84, 112.06] | <0.001 | |
| LbaMAP, mmHg | 96.98 [87.64, 105.73] | 88.33 [82.98, 96.06] | 103.60 [97.95, 113.00] | <0.001 | |
| baMAP, mmHg | 97.26 [88.08, 106.51] | 88.88 [83.08, 95.27] | 103.94 [98.46, 112.43] | <0.001 | |
| plaque, n (%) | 0 [0, 1] | 0 [0, 0] | 0 [0, 1] | NA | |
| IMT, mm | 0.71 [0.6, 1] | 0.7 [0.6, 0.94] | 0.8 [0.6, 1.06] | 0.139 | |
| China-PAR | 2.66 [1.46, 4.16] | 2.15 [1, 3.32] | 3.21 [1.86, 5.5] | <0.001 | |
| **Laboratory measurements** | | | | |  |
| SBP, mmHg | 126.5 [116, 138] | 119 [111.5, 126.62] | 134.5 [125.75, 149.5] | <0.001 | |
| DBP, mmHg | 81.96 [75.38, 89] | 78.75 [72, 84.5] | 85.5 [79, 93.12] | <0.001 | |
| PP, mmHg | 44.00 [38.00, 51.62] | 40 [35.50, 46.50] | 49.50 [42.50, 56.50] | <0.001 | |
| FG, mmol/L | 4.81 [4.36, 5.34] | 4.66 [4.24, 5.17] | 4.99 [4.5, 5.48] | <0.001 | |
| TG, mmol/L | 1.2 [0.86, 1.77] | 1.17 [0.81, 1.66] | 1.28 [0.93, 1.95] | 0.008 | |
| TC, mmol/L | 5.44 [4.88, 6.11] | 5.38 [4.82, 5.98] | 5.5 [4.92, 6.28] | 0.145 | |
| HDL-c, mmol/L | 1.37 [1.18, 1.64] | 1.4 [1.18, 1.7] | 1.36 [1.17, 1.6] | 0.096 | |
| LDL-c, mmol/L | 3.23 [2.78, 3.8] | 3.23 [2.74, 3.79] | 3.24 [2.82, 3.8] | 0.552 | |
| ALP, IU/L | 87.35 [73.62, 101.32] | 83.2 [72.83, 98.85] | 89.8 [76, 102.65] | 0.128 | |
| CREA, umol/L | 79.05 [67.62, 90.83] | 77.75 [68.5, 89.65] | 79.2 [67.2, 92.2] | 0.176 | |
| **Medication Usage** | | | | |  |
| Anti-hypertensive drugs, n (%) | 65 (8.52%) | 33 (6.55%) | 32 (12.36%) | 1 | |
| Anti-diabetic drugs, n (%) | 21 (2.75%) | 9 (1.79%) | 12 (4.63%) | 0.654 | |
| Hypolipidemic agents, n (%) | 9 (1.18%) | 6 (1.19%) | 3 (1.16%) | 0.5 | |

Data were shwon as median (interquartile range) or number (percentage). *P* value represented the difference between groups in percent (%) or median (IQR).

| **Topology** | **Metadata** | **Rho** | ***P_fdr_*** |
| --- | --- | --- | --- |
| Betweenness | baMAP | 0.19 | 0.00 |
| Betweenness | baPWV | 0.16 | 0.02 |
| Betweenness | Group | 0.20 | 0.00 |
| Betweenness | LbaMAP | 0.19 | 0.01 |
| Betweenness | LbaPWV | 0.16 | 0.03 |
| Betweenness | RbaMAP | 0.19 | 0.00 |
| Betweenness | RbaPWV | 0.17 | 0.02 |
| Clustering Coefficient | baPWV | -0.13 | 0.10 |
| Clustering Coefficient | Group | -0.12 | 0.12 |
| Clustering Coefficient | LbaPWV | -0.13 | 0.10 |
| Clustering Coefficient | RbaPWV | -0.13 | 0.10 |

**Table S3 Partial Spearman correlation between network topology and cardiovascular parameters**

**Table S4 Summary statistics of genetic instruments used in this study**

| **Exposure** | **SNP** | **A1** | **A2** | **A2 freq** | **BETA** | ***P*** | **Se** | **F value** | **Chr** | **Pos** | **H2** |
| --- | --- | --- | --- | --- | --- | --- | --- | --- | --- | --- | --- |
| g__Apiotrichum | rs12643157 | C | T | 0.937333333 | 1.453079676 | 3.44E-05 | 0.350826981 | 17.15508539 | 4 | 5158526 | 0 |
| g__Apiotrichum | rs13326249 | A | G | 0.884154461 | -1.133466199 | 3.03E-05 | 0.271716359 | 17.40145549 | 3 | 14639798 | 0 |
| g__Apiotrichum | rs1551137 | G | A | 0.733022636 | 0.815504487 | 3.03E-05 | 0.1954966 | 17.40100344 | 4 | 190438606 | 0 |
| g__Apiotrichum | rs2723359 | A | G | 0.598535286 | -0.735465209 | 2.74E-05 | 0.175373827 | 17.58711928 | 3 | 151626902 | 0 |
| g__Apiotrichum | rs2962094 | C | T | 0.715046605 | 0.823968068 | 1.34E-05 | 0.189276018 | 18.95088869 | 5 | 35134877 | 0 |
| g__Apiotrichum | rs3102165 | T | C | 0.843541944 | -0.99628942 | 3.56E-05 | 0.240982794 | 17.09223942 | 4 | 39433498 | 0 |
| g__Apiotrichum | rs312481 | A | G | 0.881491345 | -1.303461605 | 8.96E-07 | 0.265294604 | 24.14013262 | 3 | 53551902 | 0 |
| g__Apiotrichum | rs57038439 | T | C | 0.902 | -1.284787761 | 1.32E-05 | 0.294917639 | 18.97847377 | 9 | 10675420 | 0 |
| g__Apiotrichum | rs7175648 | T | C | 0.682423435 | -0.806961606 | 1.69E-05 | 0.187572742 | 18.50828979 | 15 | 97446342 | 0 |
| g__Apiotrichum | rs7605722 | A | G | 0.583333333 | -0.737598593 | 2.78E-05 | 0.176014338 | 17.56079073 | 2 | 16200946 | 0 |
| g__Apiotrichum | rs787481 | T | C | 0.587882823 | 0.798776524 | 1.49E-05 | 0.18448487 | 18.74688325 | 1 | 68095207 | 0 |
| g__Apiotrichum | rs935229 | T | G | 0.760319574 | -0.907579072 | 1.51E-05 | 0.209763354 | 18.72016656 | 12 | 41180171 | 0 |
| g__Apiotrichum | rs9519847 | C | T | 0.541944075 | -0.707282813 | 4.12E-05 | 0.172476062 | 16.81622668 | 13 | 106570407 | 0 |
| g__Apiotrichum | rs9570040 | C | T | 0.827151335 | -0.969970189 | 1.89E-05 | 0.226735914 | 18.30105564 | 13 | 59660889 | 0 |
| g__Mycothermus | rs10515007 | T | C | 0.711051931 | -0.39281661 | 1.63E-05 | 0.091140088 | 18.57636756 | 17 | 50395539 | 0 |
| g__Mycothermus | rs10932688 | C | G | 0.855140187 | -0.478219059 | 2.79E-05 | 0.114138316 | 17.55459586 | 2 | 217863481 | 0 |
| g__Mycothermus | rs118085747 | A | C | 0.939252336 | -0.712497864 | 3.61E-05 | 0.172478779 | 17.0645876 | 10 | 131277020 | 0 |
| g__Mycothermus | rs11823237 | C | T | 0.858189081 | -0.487050456 | 2.86E-05 | 0.116401894 | 17.50764823 | 11 | 60488893 | 0 |
| g__Mycothermus | rs11914603 | T | G | 0.899864682 | -0.588873262 | 2.59E-05 | 0.139988532 | 17.69533358 | 3 | 184759792 | 0 |
| g__Mycothermus | rs12048682 | G | A | 0.91011984 | -0.644257927 | 7.74E-06 | 0.144056527 | 20.00108999 | 1 | 201755933 | 0 |
| g__Mycothermus | rs12210498 | A | G | 0.507323569 | 0.340455417 | 2.80E-05 | 0.081272028 | 17.54843164 | 6 | 93917260 | 0 |
| g__Mycothermus | rs12272740 | C | T | 0.882157124 | -0.525644496 | 4.73E-05 | 0.129193348 | 16.55403767 | 11 | 60409034 | 0 |
| g__Mycothermus | rs12464018 | A | G | 0.863515313 | -0.485361967 | 3.30E-05 | 0.116902945 | 17.23775019 | 2 | 217864601 | 0 |
| g__Mycothermus | rs13086069 | C | T | 0.894141145 | -0.576437794 | 4.15E-05 | 0.140622936 | 16.80322222 | 3 | 184802703 | 0 |
| g__Mycothermus | rs13410315 | C | T | 0.576564581 | 0.332847399 | 4.61E-05 | 0.081687143 | 16.60286063 | 2 | 127554969 | 0 |
| g__Mycothermus | rs1389098 | G | A | 0.514647137 | 0.331338158 | 3.79E-05 | 0.080422012 | 16.97434551 | 4 | 142705418 | 0 |
| g__Mycothermus | rs1411276 | A | G | 0.753661784 | 0.407235853 | 3.30E-05 | 0.09808218 | 17.23898901 | 1 | 92257971 | 0 |
| g__Mycothermus | rs16856812 | C | T | 0.877496671 | -0.517933443 | 1.81E-05 | 0.120795653 | 18.38422364 | 2 | 217865319 | 0 |
| g__Mycothermus | rs1730947 | T | C | 0.870838881 | -0.502035011 | 4.29E-05 | 0.122703561 | 16.73993146 | 16 | 8804683 | 0 |
| g__Mycothermus | rs1799907 | T | A | 0.758322237 | -0.396933626 | 3.31E-05 | 0.095625305 | 17.230192 | 6 | 33152835 | 0 |
| g__Mycothermus | rs1799911 | A | G | 0.778254649 | -0.435105842 | 1.18E-05 | 0.099310409 | 19.19553813 | 6 | 33138677 | 0 |
| g__Mycothermus | rs1806201 | G | A | 0.516644474 | -0.335140105 | 4.61E-05 | 0.082249232 | 16.60309661 | 12 | 13717508 | 0 |
| g__Mycothermus | rs1927589 | T | C | 0.665113182 | -0.375875305 | 1.81E-05 | 0.087666332 | 18.38323377 | 17 | 50376564 | 0 |
| g__Mycothermus | rs2076311 | A | C | 0.765306122 | -0.417459101 | 1.57E-05 | 0.096663843 | 18.65089839 | 6 | 33145369 | 0 |
| g__Mycothermus | rs2468731 | T | C | 0.581225033 | 0.356411071 | 3.18E-05 | 0.085678666 | 17.30441715 | 8 | 140482534 | 0 |
| g__Mycothermus | rs4491709 | T | C | 0.894772118 | -0.565783284 | 9.59E-06 | 0.127827387 | 19.59081035 | 2 | 217894756 | 0 |
| g__Mycothermus | rs4838084 | A | G | 0.842210386 | -0.454767424 | 3.73E-05 | 0.11029012 | 17.00221411 | 9 | 126715275 | 0 |
| g__Mycothermus | rs4970792 | C | A | 0.795605859 | -0.434172859 | 2.24E-05 | 0.102408081 | 17.97450297 | 1 | 109155515 | 0 |
| g__Mycothermus | rs61364724 | C | A | 0.928095872 | -0.652573698 | 3.02E-05 | 0.156405771 | 17.40818867 | 17 | 9708057 | 0 |
| g__Mycothermus | rs61366588 | G | A | 0.914780293 | -0.614288989 | 3.45E-05 | 0.14833597 | 17.14954129 | 17 | 5857804 | 0 |
| g__Mycothermus | rs6435957 | T | C | 0.898135819 | -0.612402773 | 3.00E-06 | 0.131104949 | 21.81906429 | 2 | 217878209 | 0 |
| g__Mycothermus | rs6531230 | C | T | 0.937416778 | -0.742468717 | 2.52E-05 | 0.176247696 | 17.74636558 | 2 | 20520878 | 0 |
| g__Mycothermus | rs6768705 | G | A | 0.944594595 | -0.735200181 | 4.44E-05 | 0.180043791 | 16.67458032 | 3 | 103624840 | 0 |
| g__Mycothermus | rs6798029 | A | G | 0.604527297 | -0.386513803 | 1.98E-06 | 0.081285001 | 22.61044884 | 3 | 10763460 | 0 |
| g__Mycothermus | rs6848327 | T | C | 0.914780293 | -0.697241626 | 1.96E-06 | 0.146551762 | 22.63520995 | 4 | 121395702 | 0 |
| g__Mycothermus | rs6902603 | C | A | 0.525333333 | 0.348371116 | 3.05E-05 | 0.083554129 | 17.38394935 | 6 | 96384515 | 0 |
| g__Mycothermus | rs74897032 | T | G | 0.946737683 | -0.755717208 | 3.17E-05 | 0.181628424 | 17.31214944 | 22 | 48385947 | 0 |
| g__Mycothermus | rs7526426 | C | T | 0.807432432 | -0.475305003 | 5.89E-06 | 0.104914752 | 20.52445588 | 1 | 56059223 | 0 |
| g__Mycothermus | rs75890190 | T | G | 0.940079893 | -0.691913852 | 4.38E-05 | 0.169328471 | 16.69721644 | 2 | 30552223 | 0 |
| g__Mycothermus | rs76258684 | G | A | 0.916107383 | -0.578971553 | 3.17E-05 | 0.139136494 | 17.3153925 | 15 | 54696253 | 0 |
| g__Mycothermus | rs7750458 | A | G | 0.935419441 | -0.702189068 | 1.94E-05 | 0.164383507 | 18.24700721 | 6 | 33045698 | 0 |
| g__Mycothermus | rs78408796 | C | A | 0.91388518 | -0.691518384 | 1.84E-06 | 0.144955102 | 22.75833317 | 4 | 121396626 | 0 |
| g__Mycothermus | rs9277934 | T | C | 0.757656458 | -0.395012052 | 3.62E-05 | 0.095630337 | 17.06197564 | 6 | 33153528 | 0 |
| g__Mycothermus | rs9378541 | T | C | 0.937249666 | -0.691159825 | 3.31E-05 | 0.166503638 | 17.23096171 | 6 | 8342797 | 0 |
| g__Mycothermus | rs962835 | T | G | 0.669773635 | -0.35465121 | 2.66E-05 | 0.08442777 | 17.64543023 | 3 | 10764022 | 0 |
| g__Mycothermus | rs9869435 | A | G | 0.848868176 | -0.514354446 | 1.09E-05 | 0.116947605 | 19.34382413 | 3 | 184756694 | 0 |
| g__Flavocillium | rs10142901 | C | A | 0.877496671 | 0.454009957 | 2.31E-05 | 0.107257597 | 17.91738505 | 14 | 22588560 | 0 |
| g__Flavocillium | rs10837683 | G | A | 0.869507324 | -0.444646507 | 1.57E-05 | 0.102946978 | 18.65531407 | 11 | 41598851 | 0 |
| g__Flavocillium | rs11187120 | T | C | 0.888149134 | -0.516209242 | 1.02E-05 | 0.116956535 | 19.48061068 | 10 | 94415666 | 0 |
| g__Flavocillium | rs11232201 | A | G | 0.767643142 | -0.357463966 | 2.59E-05 | 0.084976913 | 17.69549208 | 11 | 80256116 | 0 |
| g__Flavocillium | rs1181531 | C | T | 0.534666667 | 0.28684325 | 4.73E-05 | 0.070500726 | 16.55396746 | 7 | 43879274 | 0 |
| g__Flavocillium | rs11818705 | T | C | 0.889480692 | -0.491508575 | 2.91E-05 | 0.117582197 | 17.47346585 | 10 | 94392728 | 0 |
| g__Flavocillium | rs12365397 | G | A | 0.734979973 | -0.354248466 | 1.32E-05 | 0.081309929 | 18.98142429 | 11 | 43236061 | 0 |
| g__Flavocillium | rs12678105 | A | C | 0.598535286 | 0.297722861 | 4.29E-05 | 0.072769697 | 16.73875812 | 8 | 61882751 | 0 |
| g__Flavocillium | rs12892757 | G | A | 0.717333333 | -0.352682908 | 6.25E-06 | 0.078067216 | 20.40945672 | 14 | 79016243 | 0 |
| g__Flavocillium | rs1417636 | T | G | 0.896804261 | -0.493215458 | 3.20E-05 | 0.118592465 | 17.29653714 | 10 | 112567414 | 0 |
| g__Flavocillium | rs1463747 | T | G | 0.844207723 | -0.404589227 | 3.44E-05 | 0.097668329 | 17.16015125 | 5 | 152913504 | 0 |
| g__Flavocillium | rs2167460 | T | C | 0.721038615 | 0.344115996 | 1.53E-05 | 0.079586641 | 18.69516796 | 6 | 56884451 | 0 |
| g__Flavocillium | rs2199246 | C | T | 0.875333333 | -0.531969666 | 6.58E-07 | 0.106960545 | 24.73583194 | 8 | 80093457 | 0 |
| g__Flavocillium | rs28403917 | A | G | 0.907616361 | -0.499724625 | 3.88E-05 | 0.121448547 | 16.93077562 | 4 | 59621069 | 0 |
| g__Flavocillium | rs4277766 | T | C | 0.933422104 | -0.706785007 | 9.30E-07 | 0.144066183 | 24.06858472 | 4 | 21910553 | 0 |
| g__Flavocillium | rs4604791 | T | C | 0.889480692 | -0.491508575 | 2.91E-05 | 0.117582197 | 17.47346585 | 10 | 94356912 | 0 |
| g__Flavocillium | rs6096928 | G | A | 0.93608522 | -0.585451661 | 3.92E-05 | 0.142368163 | 16.91049511 | 20 | 51026656 | 0 |
| g__Flavocillium | rs62384383 | T | A | 0.905206943 | -0.521049704 | 2.33E-05 | 0.123162018 | 17.89800866 | 5 | 152969986 | 0 |
| g__Flavocillium | rs6990113 | C | A | 0.927430093 | -0.587707739 | 3.17E-05 | 0.141241298 | 17.31407999 | 8 | 13578804 | 0 |
| g__Flavocillium | rs7000109 | T | C | 0.850199734 | -0.425331754 | 1.91E-05 | 0.099488991 | 18.27702749 | 8 | 80062983 | 0 |
| g__Flavocillium | rs700218 | A | C | 0.73701731 | -0.33854719 | 1.63E-05 | 0.078547984 | 18.57669042 | 5 | 39354542 | 0 |
| g__Flavocillium | rs7110368 | C | T | 0.879076087 | -0.455133886 | 1.54E-05 | 0.105295637 | 18.68347216 | 11 | 41565405 | 0 |
| g__Flavocillium | rs7529399 | C | T | 0.884 | -0.476120145 | 2.07E-05 | 0.111843221 | 18.12231959 | 1 | 7575666 | 0 |
| g__Flavocillium | rs75295976 | T | C | 0.923435419 | -0.557780088 | 4.62E-05 | 0.136911278 | 16.59768678 | 11 | 20239335 | 0 |
| g__Flavocillium | rs76201358 | A | G | 0.946071904 | -0.658543214 | 4.90E-05 | 0.162184164 | 16.48738073 | 10 | 5686458 | 0 |
| g__Flavocillium | rs7622700 | G | T | 0.693440428 | -0.327540304 | 1.10E-05 | 0.074501472 | 19.32857271 | 3 | 132446336 | 0 |
| g__Flavocillium | rs78441151 | G | A | 0.868841545 | -0.445725482 | 1.63E-05 | 0.10339298 | 18.58458302 | 11 | 41582435 | 0 |
| g__Flavocillium | rs9384362 | C | A | 0.802263648 | -0.370911612 | 3.10E-05 | 0.089028394 | 17.35736446 | 6 | 156133632 | 0 |
| g__Flavocillium | rs963208 | T | C | 0.66045273 | 0.31570678 | 3.77E-05 | 0.076609434 | 16.98256615 | 1 | 85686552 | 0 |
| g__Flavocillium | rs9837095 | A | G | 0.828 | -0.392092454 | 2.10E-05 | 0.092176302 | 18.09415935 | 3 | 167323709 | 0 |
| g__Hyaloscypha | rs10182181 | G | A | 0.556591212 | 0.303072078 | 1.97E-06 | 0.063716011 | 22.62531843 | 2 | 25150296 | 0.239093711 |
| g__Hyaloscypha | rs10496771 | C | T | 0.87816245 | -0.393426756 | 4.54E-05 | 0.096474589 | 16.6303699 | 2 | 138197269 | 0.564025607 |
| g__Hyaloscypha | rs10519766 | T | C | 0.887483356 | -0.464979698 | 1.82E-06 | 0.097434277 | 22.77426904 | 15 | 33215966 | 0.349574958 |
| g__Hyaloscypha | rs10910953 | T | G | 0.937416778 | -0.539558858 | 3.58E-05 | 0.130540701 | 17.08385088 | 1 | 181512453 | 0.421326277 |
| g__Hyaloscypha | rs10918593 | C | T | 0.726301736 | -0.28725599 | 3.39E-05 | 0.06928722 | 17.18825981 | 1 | 162030654 | 0.430235536 |
| g__Hyaloscypha | rs11619840 | A | C | 0.931424767 | -0.51537395 | 4.23E-05 | 0.125861314 | 16.76719458 | 13 | 108218958 | 0.441853991 |
| g__Hyaloscypha | rs11676272 | G | A | 0.565912117 | 0.298944181 | 2.51E-06 | 0.063504882 | 22.15980744 | 2 | 25141538 | 0.235852094 |
| g__Hyaloscypha | rs11852876 | T | C | 0.887483356 | -0.430909146 | 1.01E-05 | 0.097592144 | 19.49582889 | 15 | 33238431 | 0.358593889 |
| g__Hyaloscypha | rs11855526 | C | T | 0.886817577 | -0.461369551 | 2.11E-06 | 0.097278959 | 22.49365515 | 15 | 33218561 | 0.35493547 |
| g__Hyaloscypha | rs11923992 | A | G | 0.924766977 | -0.679339823 | 5.81E-09 | 0.116684901 | 33.89571702 | 3 | 131190769 | 0.395824226 |
| g__Hyaloscypha | rs11930532 | C | T | 0.842210386 | -0.362201092 | 3.64E-05 | 0.087711787 | 17.05232472 | 4 | 68812283 | 0.524713411 |
| g__Hyaloscypha | rs12053710 | C | A | 0.847797063 | -0.352280216 | 4.37E-05 | 0.086191728 | 16.70495569 | 21 | 26626467 | 0.497374823 |
| g__Hyaloscypha | rs12442670 | T | C | 0.758988016 | -0.308196196 | 4.13E-05 | 0.075172237 | 16.808912 | 15 | 79904871 | 0.643832434 |
| g__Hyaloscypha | rs12580453 | C | A | 0.934087883 | -0.519419252 | 3.10E-05 | 0.124687857 | 17.35352738 | 12 | 12440857 | 0.565772872 |
| g__Hyaloscypha | rs12637848 | T | C | 0.90412783 | -0.464838286 | 6.95E-06 | 0.103409366 | 20.20617122 | 3 | 131068497 | 0.4656093 |
| g__Hyaloscypha | rs12645114 | C | T | 0.780958722 | -0.318062364 | 2.42E-05 | 0.075330898 | 17.82700019 | 4 | 168543061 | 0.538347111 |
| g__Hyaloscypha | rs1364058 | A | G | 0.559920107 | -0.277214187 | 8.26E-06 | 0.06217902 | 19.87664856 | 2 | 8149627 | 0.370098226 |
| g__Hyaloscypha | rs16882265 | T | C | 0.941411451 | -0.570473618 | 1.62E-05 | 0.132297626 | 18.59375168 | 6 | 19478455 | 0.529536247 |
| g__Hyaloscypha | rs16904404 | C | T | 0.935419441 | -0.62682836 | 9.75E-07 | 0.128013568 | 23.97647159 | 8 | 132088508 | 0.602256317 |
| g__Hyaloscypha | rs16971211 | T | G | 0.792666667 | -0.337565591 | 2.15E-05 | 0.079453329 | 18.05062077 | 15 | 79943006 | 0.657887812 |
| g__Hyaloscypha | rs17153095 | C | T | 0.900133156 | -0.485198982 | 3.25E-06 | 0.104248175 | 21.66221405 | 7 | 106066417 | 0.293478841 |
| g__Hyaloscypha | rs1911845 | T | C | 0.782956059 | -0.330762742 | 1.45E-05 | 0.076277067 | 18.80378162 | 5 | 19929288 | 0.390349312 |
| g__Hyaloscypha | rs2223252 | A | G | 0.665778961 | -0.307295889 | 2.04E-06 | 0.064699685 | 22.55844545 | 20 | 8075223 | 0.417280885 |
| g__Hyaloscypha | rs2236549 | A | G | 0.586551265 | -0.274010491 | 2.79E-05 | 0.065398968 | 17.55466689 | 1 | 203670761 | 0.498969533 |
| g__Hyaloscypha | rs2289803 | G | T | 0.813581891 | -0.363600935 | 6.14E-06 | 0.080412867 | 20.44555445 | 3 | 27296212 | 0.724107809 |
| g__Hyaloscypha | rs2384061 | A | G | 0.575233023 | 0.288996402 | 6.10E-06 | 0.063895895 | 20.4568589 | 2 | 25135620 | 0.238816288 |
| g__Hyaloscypha | rs267759 | A | G | 0.747669774 | 0.326132407 | 9.35E-06 | 0.073589698 | 19.64055988 | 5 | 36137620 | 0.76438859 |
| g__Hyaloscypha | rs28409161 | A | G | 0.906125166 | -0.50359119 | 1.92E-06 | 0.105750201 | 22.67743057 | 15 | 33250654 | 0.498537525 |
| g__Hyaloscypha | rs2880058 | A | G | 0.726364847 | -0.293444401 | 2.72E-05 | 0.069936429 | 17.60535322 | 1 | 162014632 | 0.442537289 |
| g__Hyaloscypha | rs3169983 | G | A | 0.848868176 | -0.383148349 | 1.58E-05 | 0.088759141 | 18.63407037 | 18 | 61654297 | 0.538686863 |
| g__Hyaloscypha | rs35923448 | A | G | 0.916111851 | -0.628468908 | 2.22E-08 | 0.112342013 | 31.29559351 | 3 | 131222816 | 0.418492391 |
| g__Hyaloscypha | rs3749393 | C | T | 0.90562249 | -0.478143547 | 4.40E-06 | 0.104133237 | 21.0832645 | 3 | 131082393 | 0.477394979 |
| g__Hyaloscypha | rs3820363 | G | A | 0.932090546 | -0.500832608 | 4.58E-05 | 0.122873704 | 16.61373569 | 1 | 165372786 | 0.484341955 |
| g__Hyaloscypha | rs3829481 | T | C | 0.887483356 | -0.46497969 | 1.82E-06 | 0.097434279 | 22.77426727 | 15 | 33202425 | 0.349574958 |
| g__Hyaloscypha | rs3849145 | A | G | 0.600801068 | 0.28206599 | 1.17E-05 | 0.064362218 | 19.20611172 | 10 | 51049548 | 0.607624448 |
| g__Hyaloscypha | rs41473744 | A | G | 0.887483356 | -0.430909154 | 1.01E-05 | 0.097592142 | 19.4958303 | 15 | 33236850 | 0.358593889 |
| g__Hyaloscypha | rs4446102 | C | T | 0.722370173 | -0.34346816 | 7.95E-07 | 0.069574565 | 24.3709223 | 2 | 45997867 | 0.772112381 |
| g__Hyaloscypha | rs4669822 | C | T | 0.930093209 | -0.496569484 | 4.12E-05 | 0.121096411 | 16.81502471 | 2 | 12320631 | 0.471130752 |
| g__Hyaloscypha | rs4837980 | G | A | 0.941333333 | -0.541688749 | 4.41E-05 | 0.13260479 | 16.68711288 | 9 | 119649615 | 0.385190783 |
| g__Hyaloscypha | rs4860868 | G | T | 0.841544607 | -0.359308359 | 4.12E-05 | 0.087617696 | 16.81709518 | 4 | 68815847 | 0.519908852 |
| g__Hyaloscypha | rs4860878 | A | G | 0.846870839 | -0.358113266 | 4.95E-05 | 0.088246675 | 16.46812347 | 4 | 68858166 | 0.498951503 |
| g__Hyaloscypha | rs57245635 | C | T | 0.683089214 | -0.316180314 | 2.29E-06 | 0.066897108 | 22.33854909 | 16 | 59889269 | 0.533356572 |
| g__Hyaloscypha | rs57253863 | T | G | 0.613848202 | -0.280722666 | 1.40E-05 | 0.064627578 | 18.86771 | 13 | 85539064 | 0.558669956 |
| g__Hyaloscypha | rs58396975 | C | T | 0.898801598 | -0.452657924 | 1.60E-05 | 0.104915904 | 18.61476675 | 15 | 98455297 | 0.445714946 |
| g__Hyaloscypha | rs610118 | G | A | 0.899467377 | -0.440260685 | 2.60E-05 | 0.104682531 | 17.68770072 | 3 | 27336213 | 0.66196506 |
| g__Hyaloscypha | rs62228630 | G | A | 0.79494008 | -0.330446001 | 2.63E-05 | 0.078614645 | 17.66827242 | 22 | 44770316 | 0.661279574 |
| g__Hyaloscypha | rs6424815 | T | G | 0.937416778 | -0.539558862 | 3.58E-05 | 0.1305407 | 17.08385153 | 1 | 181507754 | 0.421326277 |
| g__Hyaloscypha | rs6439301 | A | G | 0.67976032 | -0.307629054 | 6.33E-06 | 0.068135804 | 20.3846815 | 3 | 131239386 | 0.99 |
| g__Hyaloscypha | rs6545814 | G | A | 0.57723036 | 0.287788175 | 7.12E-06 | 0.064093318 | 20.16138563 | 2 | 25131316 | 0.241623289 |
| g__Hyaloscypha | rs6697584 | C | T | 0.937333333 | -0.539268303 | 3.62E-05 | 0.130556086 | 17.06143444 | 1 | 181509718 | 0.422529794 |
| g__Hyaloscypha | rs6752378 | A | C | 0.55792277 | 0.299903699 | 2.39E-06 | 0.063579642 | 22.24987046 | 2 | 25150116 | 0.229933988 |
| g__Hyaloscypha | rs6976195 | G | A | 0.50665779 | -0.253127443 | 3.41E-05 | 0.061084837 | 17.17163425 | 7 | 97119556 | 0.684131904 |
| g__Hyaloscypha | rs706375 | T | C | 0.545272969 | -0.274625382 | 1.50E-05 | 0.063450779 | 18.73300412 | 1 | 56681024 | 0.601917565 |
| g__Hyaloscypha | rs72719309 | T | C | 0.891129032 | -0.467740233 | 2.16E-06 | 0.098733409 | 22.44301456 | 15 | 33198440 | 0.338353979 |
| g__Hyaloscypha | rs73081241 | T | C | 0.905459387 | -0.450026623 | 2.00E-05 | 0.105506127 | 18.19370081 | 12 | 25409782 | 0.574987666 |
| g__Hyaloscypha | rs74795342 | A | G | 0.893475366 | -0.427817597 | 1.59E-05 | 0.099119368 | 18.62945868 | 21 | 20326336 | 0.615671212 |
| g__Hyaloscypha | rs75222709 | G | T | 0.892809587 | -0.423075649 | 1.91E-05 | 0.098949062 | 18.2815365 | 21 | 20327427 | 0.60119049 |
| g__Hyaloscypha | rs7623918 | A | G | 0.870173103 | -0.447670544 | 9.57E-07 | 0.091357417 | 24.01205987 | 3 | 131078605 | 0.473784731 |
| g__Hyaloscypha | rs7713161 | A | G | 0.58988016 | -0.278391466 | 1.18E-05 | 0.06354411 | 19.1938118 | 5 | 17796513 | 0.557770876 |
| g__Hyaloscypha | rs7728335 | A | G | 0.645139814 | -0.280436856 | 2.51E-05 | 0.06655393 | 17.75508576 | 5 | 150975571 | 0.42638388 |
| g__Hyaloscypha | rs78197736 | A | G | 0.921438083 | -0.505373161 | 1.93E-05 | 0.118260303 | 18.2619173 | 12 | 3671556 | 0.502395193 |
| g__Hyaloscypha | rs7960599 | G | A | 0.918109188 | -0.476966654 | 4.27E-05 | 0.116545185 | 16.7489413 | 12 | 3656119 | 0.527525219 |
| g__Hyaloscypha | rs79663003 | C | T | 0.900133156 | -0.416681018 | 3.84E-05 | 0.101205066 | 16.9512967 | 21 | 20310893 | 0.608705481 |
| g__Hyaloscypha | rs8027369 | G | A | 0.887483356 | -0.464979758 | 1.82E-06 | 0.09743426 | 22.77428318 | 15 | 33198928 | 0.349574958 |
| g__Hyaloscypha | rs827540 | T | C | 0.922043011 | -0.504167029 | 1.30E-05 | 0.115628558 | 19.0115996 | 8 | 104299426 | 0.38175843 |
| g__Hyaloscypha | rs944722 | C | T | 0.683823529 | -0.328820257 | 5.99E-06 | 0.072640613 | 20.49076266 | 17 | 26092037 | 0.459713391 |
| g__Hyaloscypha | rs9531686 | T | G | 0.682550336 | -0.342268303 | 3.41E-07 | 0.067120559 | 26.0029267 | 13 | 85549736 | 0.572155171 |
| g__Hyaloscypha | rs9546986 | G | T | 0.717043941 | -0.349883253 | 5.37E-07 | 0.069802201 | 25.12511606 | 13 | 85543153 | 0.491947177 |
| g__Microscypha | rs1058867 | G | A | 0.752330226 | -0.489559443 | 1.38E-05 | 0.112610211 | 18.89971773 | 21 | 34669381 | 0 |
| g__Microscypha | rs10793350 | C | T | 0.736351531 | -0.461605309 | 4.32E-05 | 0.112875962 | 16.72394228 | 11 | 78694515 | 0 |
| g__Microscypha | rs112020848 | A | G | 0.933770015 | -0.999300979 | 2.60E-06 | 0.212607379 | 22.09204704 | 13 | 91490657 | 0 |
| g__Microscypha | rs115635229 | C | T | 0.91011984 | -0.772238378 | 6.68E-06 | 0.171473577 | 20.28188651 | 6 | 95509314 | 0 |
| g__Microscypha | rs11896914 | T | C | 0.949197861 | -0.973198505 | 2.78E-05 | 0.232242069 | 17.5598613 | 2 | 37904652 | 0.014187574 |
| g__Microscypha | rs1869002 | G | A | 0.695302013 | -0.441752944 | 1.97E-05 | 0.103493043 | 18.2195059 | 11 | 3870463 | 0 |
| g__Microscypha | rs1954987 | G | A | 0.780292943 | -0.47795803 | 3.63E-05 | 0.115727849 | 17.05704383 | 18 | 71249180 | 0 |
| g__Microscypha | rs28625495 | T | C | 0.939333333 | -0.851651269 | 2.06E-05 | 0.199984876 | 18.13548973 | 4 | 8585517 | 0 |
| g__Microscypha | rs4278754 | G | A | 0.819333333 | -0.554841702 | 2.21E-05 | 0.130777546 | 17.9999718 | 16 | 26356098 | 0 |
| g__Microscypha | rs4640110 | G | A | 0.529294274 | -0.411061296 | 3.66E-05 | 0.099579487 | 17.04014964 | 14 | 81692191 | 0 |
| g__Microscypha | rs59375486 | G | A | 0.943408788 | -0.922412767 | 1.82E-05 | 0.215215233 | 18.36980711 | 11 | 134357770 | 0 |
| g__Microscypha | rs61802608 | G | T | 0.853878116 | -0.590215202 | 2.81E-05 | 0.140922789 | 17.54116059 | 1 | 160530637 | 0 |
| g__Microscypha | rs66593595 | C | T | 0.57057257 | -0.452800428 | 7.05E-06 | 0.100794887 | 20.18071984 | 9 | 16687437 | 0 |
| g__Microscypha | rs66816129 | G | T | 0.683578104 | -0.416216676 | 4.51E-05 | 0.102022248 | 16.64367293 | 11 | 3879472 | 0 |
| g__Microscypha | rs6980320 | G | A | 0.772969374 | -0.487975635 | 4.12E-05 | 0.11899889 | 16.81552588 | 7 | 26653490 | 0 |
| g__Microscypha | rs7038172 | C | T | 0.553928096 | -0.471064857 | 2.28E-06 | 0.099661361 | 22.34126609 | 9 | 16708269 | 0 |
| g__Microscypha | rs7235306 | A | G | 0.659121172 | 0.440070302 | 2.27E-05 | 0.103874125 | 17.94854974 | 18 | 57216026 | 0 |
| g__Microscypha | rs7603072 | C | T | 0.533954727 | 0.424513071 | 3.36E-05 | 0.10234625 | 17.20434999 | 2 | 137209221 | 0 |
| g__Microscypha | rs76172984 | G | A | 0.888814913 | -0.647058327 | 4.14E-05 | 0.157848066 | 16.80383196 | 2 | 116453531 | 0 |
| g__Microscypha | rs78992631 | A | G | 0.910785619 | -0.729540563 | 2.26E-05 | 0.172177215 | 17.95343406 | 6 | 95512321 | 0 |
| g__Microscypha | rs9683869 | A | G | 0.946071904 | -0.916090825 | 2.39E-05 | 0.216817169 | 17.85211719 | 4 | 8585665 | 0 |

**Table S5 Forward MR results**

| **Exposure** | **Outcome** | **No. of SNPs** | **Method** | ***P* value** | **Estimate** | **Lower** | **Upper** |
| --- | --- | --- | --- | --- | --- | --- | --- |
| Apiotrichum | baPWV | 14 | IVW | 0.041 | 0.007 | 0 | 0.013 |
| Apiotrichum | baPWV | 14 | Weighted | 0.058 | 0.008 | 0 | 0.017 |
| Apiotrichum | baPWV | 14 | MR-Egger | 0.538 | 0.010 | -0.021 | 0.041 |
| Apiotrichum | baPWV | 14 | MR-Egger Intercept | 0.848 | -0.003 | -0.031 | 0.025 |
| Mycothermus | baPWV | 42 | IVW | 0.003 | -0.012 | -0.02 | -0.004 |
| Mycothermus | baPWV | 42 | Weighted | 0.028 | -0.013 | -0.024 | -0.001 |
| Mycothermus | baPWV | 42 | MR-Egger | 0.779 | -0.005 | -0.037 | 0.028 |
| Mycothermus | baPWV | 42 | MR-Egger Intercept | 0.643 | -0.004 | -0.019 | 0.012 |
| Flavocillium | Group | 30 | IVW | 0.033 | -0.018 | -0.035 | -0.001 |
| Flavocillium | Group | 30 | Weighted | 0.006 | -0.550 | -0.941 | -0.159 |
| Flavocillium | Group | 30 | MR-Egger | 0.489 | -0.034 | -0.131 | 0.063 |
| Flavocillium | Group | 30 | MR-Egger Intercept | 0.743 | 0.008 | -0.038 | 0.053 |
| Hyaloscypha | Group | 68 | IVW | 0.012 | -0.011 | -0.019 | -0.002 |
| Hyaloscypha | Group | 68 | Weighted | 0.000 | -1.788 | -2.35 | -1.225 |
| Hyaloscypha | Group | 68 | MR-Egger | 0.291 | -0.017 | -0.048 | 0.014 |
| Hyaloscypha | Group | 68 | MR-Egger Intercept | 0.694 | 0.002 | -0.009 | 0.013 |
| Microscypha | Group | 21 | IVW | 0.012 | -0.020 | -0.357 | -0.044 |
| Microscypha | Group | 21 | Weighted | 0.012 | -0.828 | -1.477 | -0.18 |
| Microscypha | Group | 21 | MR-Egger | 0.871 | -0.054 | -0.705 | 0.597 |
| Microscypha | Group | 21 | MR-Egger Intercept | 0.649 | -0.121 | -0.641 | 0.400 |

**Table S6 Backward MR results**

| **Exposure** | **Outcome** | **No. of SNPs** | **Method** | ***P* value** | **Estimate** | **Lower** | **Upper** |
| --- | --- | --- | --- | --- | --- | --- | --- |
| baPWV | Apiotrichum | 14 | IVW | 0.015 | 46.628 | 9.076 | 84.181 |
| baPWV | Apiotrichum | 14 | Weighted | 0.02 | 57.449 | 8.87 | 106.027 |
| baPWV | Apiotrichum | 14 | MR-Egger | 0.001 | 101.955 | 42.535 | 161.375 |
| baPWV | Apiotrichum | 14 | MR-Egger Intercept | 0.028 | -0.719 | -1.362 | -0.077 |
| baPWV | Mycothermus | 42 | IVW | 0.003 | -14.388 | -24.034 | -4.742 |
| baPWV | Mycothermus | 42 | Weighted | 0 | -21.744 | -29.745 | -13.743 |
| baPWV | Mycothermus | 42 | MR-Egger | 0.657 | -3.28 | -17.764 | 11.204 |
| baPWV | Mycothermus | 42 | MR-Egger Intercept | 0.049 | -0.197 | -0.393 | 0 |
| Group | Flavocillium | 30 | IVW | 0.178 | -0.068 | -0.167 | 0.031 |
| Group | Flavocillium | 30 | Weighted | 0.002 | -0.787 | -1.285 | -0.289 |
| Group | Flavocillium | 30 | MR-Egger | 0.651 | 0.023 | -0.076 | 0.122 |
| Group | Flavocillium | 30 | MR-Egger Intercept | 0.001 | -0.258 | -0.406 | -0.111 |
| Group | Hyaloscypha | 68 | IVW | 0 | -0.099 | -0.142 | -0.055 |
| Group | Hyaloscypha | 68 | Weighted | 0 | -0.389 | -0.538 | -0.24 |
| Group | Hyaloscypha | 68 | MR-Egger | 0.885 | 0.004 | -0.046 | 0.053 |
| Group | Hyaloscypha | 68 | MR-Egger Intercept | 0 | -0.264 | -0.354 | -0.175 |
| Group | Microscypha | 21 | IVW | 0.051 | -0.097 | -0.195 | 0 |
| Group | Microscypha | 21 | Weighted | 0 | -0.866 | -0.134 | -0.392 |
| Group | Microscypha | 21 | MR-Egger | 0.176 | 0.072 | -0.032 | 0.177 |
| Group | Microscypha | 21 | MR-Egger Intercept | 0 | -0.515 | -0.748 | -0.282 |

**Table S7 Mediation analysis results**

| **Exposure** | **Mediator** | **Outcome** | ***P*_mediation_** | **Prop.** | ***P*_Exposure-Outcome_** | ***P*_Exposure-Mediator_** | ***P*_Mediator-Outcome_** |
| --- | --- | --- | --- | --- | --- | --- | --- |
| Apiotrichum | Carnitine C5:1 | baPWV | 0.04 | 53.14% | < 0.01 | 0.04 | < 0.01 |
| Apiotrichum | Carnitine C11:1 | baPWV | 0.03 | 42.74% | < 0.01 | 0.02 | < 0.01 |
| Apiotrichum | Adenosine 5'-Monophosphate | baPWV | < 0.01 | 38.56% | < 0.01 | < 0.01 | < 0.01 |
| Apiotrichum | 2'-O-Methyladenosine | baPWV | < 0.01 | 38.14% | < 0.01 | < 0.01 | < 0.01 |
| Apiotrichum | 3'-Aenylic Acid | baPWV | < 0.01 | 32.82% | < 0.01 | 0.01 | < 0.01 |
| Apiotrichum | Carnitine C11:0 | baPWV | 0.02 | 31.04% | < 0.01 | 0.05 | < 0.01 |
| Apiotrichum | Carnitine C8-OH | baPWV | 0.01 | 30.60% | < 0.01 | 0.01 | < 0.01 |
| Apiotrichum | Carnitine C9:DC | baPWV | 0.02 | 30.00% | < 0.01 | 0.03 | < 0.01 |
| Apiotrichum | 12-hydroxyeicosatetraenoic acid | baPWV | 0.02 | 28.78% | < 0.01 | < 0.01 | < 0.01 |
| Apiotrichum | Carnitine C12:1 | baPWV | 0.02 | 28.71% | < 0.01 | 0.04 | < 0.01 |
| Apiotrichum | Carnitine C12:0 | baPWV | 0.05 | 27.78% | < 0.01 | 0.05 | < 0.01 |
| Apiotrichum | Carnitine C14:2 Isomer 1 | baPWV | < 0.01 | 27.40% | < 0.01 | 0.02 | < 0.01 |
| Apiotrichum | Bis(1-inositol) -3,1'-phosphate 1-phosphate | baPWV | 0.01 | 27.29% | < 0.01 | 0.01 | < 0.01 |
| Apiotrichum | 8-iso Prostaglandin F1alpha | baPWV | 0.05 | 27.24% | < 0.01 | 0.05 | < 0.01 |
| Apiotrichum | 9,10,13-TriHOME | baPWV | 0.04 | 25.79% | < 0.01 | < 0.01 | < 0.01 |
| Apiotrichum | Sucrose | baPWV | 0.04 | 25.74% | < 0.01 | 0.03 | < 0.01 |
| Apiotrichum | Carnitine C14:3 | baPWV | 0.01 | 25.47% | < 0.01 | 0.01 | < 0.01 |
| Apiotrichum | Carnitine C9:1 | baPWV | 0.02 | 24.69% | < 0.01 | 0.02 | < 0.01 |
| Apiotrichum | Carnitine C10:1 Isomer1 | baPWV | 0.01 | 24.31% | < 0.01 | 0.01 | < 0.01 |
| Apiotrichum | Carnitine C10:1 | baPWV | < 0.01 | 23.50% | < 0.01 | 0.01 | < 0.01 |
| Apiotrichum | 1-Methyluric acid | baPWV | 0.04 | 22.51% | < 0.01 | 0.03 | < 0.01 |
| Mycothermus | L-Threonine | baPWV | 0.01 | 22.10% | < 0.01 | 0.03 | < 0.01 |
| Mycothermus | L-Homoserine | baPWV | 0.03 | 22.10% | < 0.01 | 0.03 | < 0.01 |
| Apiotrichum | Trans-4-Hydroxy-L-Proline | baPWV | 0.02 | 21.92% | < 0.01 | 0.03 | < 0.01 |
| Apiotrichum | Phosphoenolpyruvate | baPWV | 0.04 | 21.71% | < 0.01 | 0.05 | < 0.01 |
| Apiotrichum | Carnitine C8:0 | baPWV | 0.02 | 21.68% | < 0.01 | 0.01 | < 0.01 |
| Apiotrichum | Carnitine C10:2 | baPWV | 0.04 | 21.48% | < 0.01 | 0.01 | < 0.01 |
| Apiotrichum | UNII-0TM46496W4 | baPWV | 0.03 | 20.81% | < 0.01 | 0.05 | < 0.01 |
| Apiotrichum | Sphingosine | baPWV | 0.04 | 20.75% | < 0.01 | 0.04 | < 0.01 |
| Apiotrichum | 3-Dehydrosphinganine | baPWV | 0.04 | 20.75% | < 0.01 | 0.04 | < 0.01 |
| Apiotrichum | Carnitine C10:0 | baPWV | 0.01 | 20.21% | < 0.01 | 0.02 | < 0.01 |
| Apiotrichum | phosphonoacetic acid | baPWV | 0.03 | 20.17% | < 0.01 | 0.02 | < 0.01 |
| Apiotrichum | Octanoylcarnitine | baPWV | < 0.01 | 19.38% | < 0.01 | 0.03 | < 0.01 |
| Apiotrichum | Carnitine C6:0 Isomer 2 | baPWV | < 0.01 | 18.94% | < 0.01 | 0.03 | < 0.01 |
| Apiotrichum | Carnitine C6:0 Isomer 1 | baPWV | 0.01 | 17.88% | < 0.01 | 0.04 | < 0.01 |
| Apiotrichum | Decanoyl L-Carnitine | baPWV | 0.01 | 16.50% | < 0.01 | 0.03 | < 0.01 |
| Mycothermus | Hydroxypiperazic acid | baPWV | 0.04 | 15.71% | < 0.01 | 0.02 | < 0.01 |
| Mycothermus | C18(Plasm) LPC | baPWV | 0.01 | 13.03% | < 0.01 | 0.03 | < 0.01 |
| Microscypha | Glutamine | baPWV | 0.04 | 12.34% | 0.04 | 0.02 | 0.01 |

**Table S8 downstream target analysis**

| **Metabolites** | **Target** | **Target Class** | **Probability*** |
| --- | --- | --- | --- |
| Hydroxypiperazic acid | FBP1 | Enzyme | 0.024 |
| Glutamine | GRIA1 | Ligand-gated ion channel | 0.126 |
| Glutamine | GRIA4 | Ligand-gated ion channel | 0.126 |
| Glutamine | GRIA2 | Ligand-gated ion channel | 0.126 |
| Glutamine | GRIK5 | Ligand-gated ion channel | 0.115 |
| C18(Plasm) LPC | LPAR3 | Family A G protein-coupled receptor | 0.120 |
| C18(Plasm) LPC | LPAR1 | Family A G protein-coupled receptor | 0.120 |
| 1-Methyluric acid | KCNH2 | Voltage-gated ion channel | 0.151 |
| 1-Methyluric acid | ADORA1 | Family A G protein-coupled receptor | 0.142 |
| 1-Methyluric acid | ACHE | Hydrolase | 0.125 |
| 1-Methyluric acid | ADORA2A | Family A G protein-coupled receptor | 0.116 |
| 1-Methyluric acid | ADORA2B | Family A G protein-coupled receptor | 0.116 |
| 1-Methyluric acid | ADORA3 | Family A G protein-coupled receptor | 0.107 |
| 12-hydroxyeicosatetraenoic acid | PPARG | Nuclear receptor | 0.118 |
| 12-hydroxyeicosatetraenoic acid | LTB4R | Family A G protein-coupled receptor | 0.110 |
| 12-hydroxyeicosatetraenoic acid | ALOX5 | Oxidoreductase | 0.102 |
| 12-hydroxyeicosatetraenoic acid | PPARA | Nuclear receptor | 0.102 |
| 12-hydroxyeicosatetraenoic acid | PPARD | Nuclear receptor | 0.102 |
| 12-hydroxyeicosatetraenoic acid | TBXAS1 | Cytochrome P450 | 0.102 |
| 12-hydroxyeicosatetraenoic acid | AGTR1 | Family A G protein-coupled receptor | 0.102 |
| 12-hydroxyeicosatetraenoic acid | CNR1 | Family A G protein-coupled receptor | 0.102 |
| 12-hydroxyeicosatetraenoic acid | PTGDR2 | Family A G protein-coupled receptor | 0.102 |
| 12-hydroxyeicosatetraenoic acid | THRA | Nuclear receptor | 0.102 |
| 12-hydroxyeicosatetraenoic acid | THRB | Nuclear receptor | 0.102 |
| 12-hydroxyeicosatetraenoic acid | ALOX15 | Enzyme | 0.102 |
| 12-hydroxyeicosatetraenoic acid | ALOX12 | Enzyme | 0.102 |
| 12-hydroxyeicosatetraenoic acid | TTL | Enzyme | 0.102 |
| 12-hydroxyeicosatetraenoic acid | IMPDH1 | Oxidoreductase | 0.102 |
| 12-hydroxyeicosatetraenoic acid | IMPDH2 | Oxidoreductase | 0.102 |
| 12-hydroxyeicosatetraenoic acid | FABP4 | Fatty acid binding protein family | 0.102 |
| 12-hydroxyeicosatetraenoic acid | PTGER2 | Family A G protein-coupled receptor | 0.102 |
| 12-hydroxyeicosatetraenoic acid | CASP3 | Protease | 0.102 |
| 12-hydroxyeicosatetraenoic acid | CASP7 | Protease | 0.102 |
| 12-hydroxyeicosatetraenoic acid | CASP8 | Protease | 0.102 |
| 12-hydroxyeicosatetraenoic acid | SIRT1 | Eraser | 0.102 |
| 12-hydroxyeicosatetraenoic acid | MMP13 | Protease | 0.102 |
| 12-hydroxyeicosatetraenoic acid | MMP3 | Protease | 0.102 |
| 12-hydroxyeicosatetraenoic acid | MMP2 | Protease | 0.102 |
| 12-hydroxyeicosatetraenoic acid | CCR1 | Family A G protein-coupled receptor | 0.102 |
| 12-hydroxyeicosatetraenoic acid | JAK3 | Kinase | 0.102 |
| 12-hydroxyeicosatetraenoic acid | ACE | Protease | 0.102 |
| 12-hydroxyeicosatetraenoic acid | SYK | Kinase | 0.102 |
| 12-hydroxyeicosatetraenoic acid | GSK3B | Kinase | 0.102 |
| 12-hydroxyeicosatetraenoic acid | GSK3A | Kinase | 0.102 |
| 12-hydroxyeicosatetraenoic acid | MME | Protease | 0.102 |
| 12-hydroxyeicosatetraenoic acid | HSD17B2 | Enzyme | 0.102 |
| 12-hydroxyeicosatetraenoic acid | ITGB1 | Membrane receptor | 0.102 |
| 12-hydroxyeicosatetraenoic acid | ITGA4 | Membrane receptor | 0.102 |
| 12-hydroxyeicosatetraenoic acid | NR1H4 | Nuclear receptor | 0.102 |
| 12-hydroxyeicosatetraenoic acid | ITGB7 | Membrane receptor | 0.102 |
| 12-hydroxyeicosatetraenoic acid | ITGA4 | Membrane receptor | 0.102 |
| 12-hydroxyeicosatetraenoic acid | AMPD3 | Enzyme | 0.102 |
| 12-hydroxyeicosatetraenoic acid | BCL2 | Other ion channel | 0.102 |
| 12-hydroxyeicosatetraenoic acid | CYP19A1 | Cytochrome P450 | 0.102 |
| 12-hydroxyeicosatetraenoic acid | MDM2 | Other nuclear protein | 0.102 |
| 12-hydroxyeicosatetraenoic acid | TYMS | Transferase | 0.102 |
| 12-hydroxyeicosatetraenoic acid | TRPM8 | Voltage-gated ion channel | 0.102 |
| 12-hydroxyeicosatetraenoic acid | CASP1 | Protease | 0.102 |
| 12-hydroxyeicosatetraenoic acid | PTGER1 | Family A G protein-coupled receptor | 0.102 |
| 12-hydroxyeicosatetraenoic acid | PDE5A | Phosphodiesterase | 0.102 |
| 12-hydroxyeicosatetraenoic acid | ITGAV | Membrane receptor | 0.102 |
| 12-hydroxyeicosatetraenoic acid | ITGB3 | Membrane receptor | 0.102 |
| 12-hydroxyeicosatetraenoic acid | MAPK10 | Kinase | 0.102 |
| 12-hydroxyeicosatetraenoic acid | MCL1 | Other cytosolic protein | 0.102 |
| 12-hydroxyeicosatetraenoic acid | ITGA2B | Membrane receptor | 0.102 |
| 12-hydroxyeicosatetraenoic acid | ITGB3 | Membrane receptor | 0.102 |
| 12-hydroxyeicosatetraenoic acid | ITGB5 | Membrane receptor | 0.102 |
| 12-hydroxyeicosatetraenoic acid | ITGAV | Membrane receptor | 0.102 |
| 12-hydroxyeicosatetraenoic acid | ITGAV | Membrane receptor | 0.102 |
| 12-hydroxyeicosatetraenoic acid | ITGB6 | Membrane receptor | 0.102 |
| 12-hydroxyeicosatetraenoic acid | IKBKB | Kinase | 0.102 |
| 12-hydroxyeicosatetraenoic acid | PTGER4 | Family A G protein-coupled receptor | 0.102 |
| 12-hydroxyeicosatetraenoic acid | SOAT1 | Enzyme | 0.102 |
| 12-hydroxyeicosatetraenoic acid | CYP2C9 | Cytochrome P450 | 0.102 |
| 12-hydroxyeicosatetraenoic acid | DGAT1 | Enzyme | 0.102 |
| 12-hydroxyeicosatetraenoic acid | CPT1A | Enzyme | 0.102 |
| 12-hydroxyeicosatetraenoic acid | FOLH1 | Protease | 0.102 |
| 12-hydroxyeicosatetraenoic acid | CPT1B | Group translocator | 0.102 |
| 12-hydroxyeicosatetraenoic acid | CPT2 | Enzyme | 0.102 |
| 12-hydroxyeicosatetraenoic acid | GCGR | Family B G protein-coupled receptor | 0.102 |
| 12-hydroxyeicosatetraenoic acid | PTPN1 | Phosphatase | 0.102 |
| 12-hydroxyeicosatetraenoic acid | PTGER3 | Family A G protein-coupled receptor | 0.102 |
| 12-hydroxyeicosatetraenoic acid | ADAMTS5 | Protease | 0.102 |
| 12-hydroxyeicosatetraenoic acid | AMPD2 | Enzyme | 0.102 |
| 12-hydroxyeicosatetraenoic acid | MMP14 | Protease | 0.102 |
| 12-hydroxyeicosatetraenoic acid | GRM2 | Family C G protein-coupled receptor | 0.102 |
| 12-hydroxyeicosatetraenoic acid | GALK1 | Enzyme | 0.102 |
| 12-hydroxyeicosatetraenoic acid | AKR1C3 | Enzyme | 0.102 |
| 12-hydroxyeicosatetraenoic acid | CDK2 | Other cytosolic protein | 0.102 |
| 12-hydroxyeicosatetraenoic acid | CCNA1 | Other cytosolic protein | 0.102 |
| 12-hydroxyeicosatetraenoic acid | CCNA2 | Other cytosolic protein | 0.102 |
| 12-hydroxyeicosatetraenoic acid | F10 | Protease | 0.102 |
| 12-hydroxyeicosatetraenoic acid | GRIN2D | Ligand-gated ion channel | 0.102 |
| 12-hydroxyeicosatetraenoic acid | MMP1 | Protease | 0.102 |
| 12-hydroxyeicosatetraenoic acid | PDE6D | Phosphodiesterase | 0.102 |
| 12-hydroxyeicosatetraenoic acid | S1PR2 | Family A G protein-coupled receptor | 0.102 |
| 12-hydroxyeicosatetraenoic acid | AURKA | Kinase | 0.102 |
| 12-hydroxyeicosatetraenoic acid | EGFR | Kinase | 0.102 |
| 12-hydroxyeicosatetraenoic acid | PTGIS | Cytochrome P450 | 0.102 |
| 12-hydroxyeicosatetraenoic acid | ECE1 | Protease | 0.102 |
| 12-hydroxyeicosatetraenoic acid | LIMK2 | Kinase | 0.102 |
| 12-hydroxyeicosatetraenoic acid | CYP26A1 | Cytochrome P450 | 0.102 |
| 12-hydroxyeicosatetraenoic acid | RARG | Nuclear receptor | 0.102 |
| 12-hydroxyeicosatetraenoic acid | RARA | Nuclear receptor | 0.102 |
| 12-hydroxyeicosatetraenoic acid | ADAMTS4 | Protease | 0.102 |
| 12-hydroxyeicosatetraenoic acid | P2RX3 | Ligand-gated ion channel | 0.102 |
| 12-hydroxyeicosatetraenoic acid | FGFR1 | Kinase | 0.102 |
| 12-hydroxyeicosatetraenoic acid | CYP26B1 | Cytochrome P450 | 0.102 |
| 12-hydroxyeicosatetraenoic acid | FAAH | Enzyme | 0.102 |
| 12-hydroxyeicosatetraenoic acid | EDNRB | Family A G protein-coupled receptor | 0.102 |
| 12-hydroxyeicosatetraenoic acid | CPA1 | Protease | 0.102 |
| 12-hydroxyeicosatetraenoic acid | OPRM1 | Family A G protein-coupled receptor | 0.102 |
| 12-hydroxyeicosatetraenoic acid | OPRD1 | Family A G protein-coupled receptor | 0.102 |
| 12-hydroxyeicosatetraenoic acid | EDNRA | Family A G protein-coupled receptor | 0.102 |
| 12-hydroxyeicosatetraenoic acid | TP53 | Transcription factor | 0.102 |
| 12-hydroxyeicosatetraenoic acid | SLC6A1 | Electrochemical transporter | 0.102 |
| 12-hydroxyeicosatetraenoic acid | DPP4 | Protease | 0.102 |
| 12-hydroxyeicosatetraenoic acid | RCE1 | Protease | 0.102 |
| 12-hydroxyeicosatetraenoic acid | CMA1 | Protease | 0.102 |
| 12-hydroxyeicosatetraenoic acid | BCL2L2 | Other cytosolic protein | 0.102 |
| 12-hydroxyeicosatetraenoic acid | BCL2A1 | Unclassified protein | 0.102 |
| 8-iso Prostaglandin F1alpha | PTGFR | Family A G protein-coupled receptor | 0.692 |
| 8-iso Prostaglandin F1alpha | PTGER1 | Family A G protein-coupled receptor | 0.633 |
| 8-iso Prostaglandin F1alpha | PTGER4 | Family A G protein-coupled receptor | 0.633 |
| 8-iso Prostaglandin F1alpha | PTGER3 | Family A G protein-coupled receptor | 0.599 |
| 8-iso Prostaglandin F1alpha | PTGDR | Family A G protein-coupled receptor | 0.599 |
| 8-iso Prostaglandin F1alpha | PPARG | Nuclear receptor | 0.214 |
| 8-iso Prostaglandin F1alpha | PPARA | Nuclear receptor | 0.214 |
| 8-iso Prostaglandin F1alpha | PPARD | Nuclear receptor | 0.214 |
| 8-iso Prostaglandin F1alpha | PTGER2 | Family A G protein-coupled receptor | 0.173 |
| 8-iso Prostaglandin F1alpha | PTGIR | Family A G protein-coupled receptor | 0.156 |
| 8-iso Prostaglandin F1alpha | SLC22A6 | Electrochemical transporter | 0.139 |
| 8-iso Prostaglandin F1alpha | LTB4R | Family A G protein-coupled receptor | 0.114 |
| 8-iso Prostaglandin F1alpha | SLC6A4 | Electrochemical transporter | 0.106 |
| Sucrose | CDK1 | Kinase | 0.127 |
| Sucrose | HSP90AA1 | Other cytosolic protein | 0.118 |
| Sucrose | VEGFA | Secreted protein | 0.102 |
| Sucrose | PSEN2 | Protease | 0.102 |
| Sucrose | PSENEN | Protease | 0.102 |
| Sucrose | NCSTN | Protease | 0.102 |
| Sucrose | APH1A | Protease | 0.102 |
| Sucrose | PSEN1 | Protease | 0.102 |
| Sucrose | APH1B | Protease | 0.102 |
| Sucrose | FGF1 | Secreted protein | 0.102 |
| Sucrose | HPSE | Enzyme | 0.102 |
| Sucrose | FGF2 | Secreted protein | 0.102 |
| Sucrose | LGALS4 | Other cytosolic protein | 0.102 |
| Sucrose | LGALS3 | Other cytosolic protein | 0.102 |
| Sucrose | LGALS8 | Other cytosolic protein | 0.102 |
| Sucrose | HTR2B | Family A G protein-coupled receptor | 0.102 |
| Sucrose | ADRA2A | Family A G protein-coupled receptor | 0.102 |
| Sucrose | ADRA2C | Family A G protein-coupled receptor | 0.102 |
| Sucrose | ADRA2B | Family A G protein-coupled receptor | 0.102 |
| Sucrose | DRD1 | Family A G protein-coupled receptor | 0.102 |
| Sucrose | DRD2 | Family A G protein-coupled receptor | 0.102 |
| Sucrose | ADRA1D | Family A G protein-coupled receptor | 0.102 |
| Sucrose | HTR2A | Family A G protein-coupled receptor | 0.102 |
| Sucrose | HTR2C | Family A G protein-coupled receptor | 0.102 |
| Sucrose | DRD3 | Family A G protein-coupled receptor | 0.102 |
| Sucrose | CYP2D6 | Cytochrome P450 | 0.102 |
| Sucrose | HTR6 | Family A G protein-coupled receptor | 0.102 |
| Sucrose | ADRA1A | Family A G protein-coupled receptor | 0.102 |
| Sucrose | HTR1B | Family A G protein-coupled receptor | 0.102 |
| Sucrose | FOLH1 | Protease | 0.102 |
| Sucrose | RORC | Nuclear receptor | 0.102 |
| Sucrose | TRPV1 | Voltage-gated ion channel | 0.102 |
| Sucrose | STAT3 | Transcription factor | 0.102 |
| Sucrose | PYGL | Enzyme | 0.102 |
| Carnitine C9:1 | SLC25A20 | Electrochemical transporter | 0.641 |
| Carnitine C9:1 | NR1H4 | Nuclear receptor | 0.411 |
| Decanoyl L-Carnitine | SLC25A20 | Electrochemical transporter | 0.996 |
| Decanoyl L-Carnitine | NR1H4 | Nuclear receptor | 0.499 |
| Decanoyl L-Carnitine | CPT1A | Enzyme | 0.112 |
| Decanoyl L-Carnitine | OXER1 | Family A G protein-coupled receptor | 0.112 |
| Decanoyl L-Carnitine | PTGER1 | Family A G protein-coupled receptor | 0.112 |
| Decanoyl L-Carnitine | PTGER2 | Family A G protein-coupled receptor | 0.112 |
| Decanoyl L-Carnitine | PTGDR | Family A G protein-coupled receptor | 0.112 |
| Decanoyl L-Carnitine | PTGER4 | Family A G protein-coupled receptor | 0.112 |
| Decanoyl L-Carnitine | CPT1B | Group translocator | 0.112 |
| Decanoyl L-Carnitine | TBXA2R | Family A G protein-coupled receptor | 0.112 |
| Decanoyl L-Carnitine | PTGDR2 | Family A G protein-coupled receptor | 0.112 |
| Decanoyl L-Carnitine | NR4A1 | Nuclear receptor | 0.112 |
| Decanoyl L-Carnitine | ESR2 | Nuclear receptor | 0.112 |
| Decanoyl L-Carnitine | CNR1 | Family A G protein-coupled receptor | 0.112 |
| Decanoyl L-Carnitine | PPARG | Nuclear receptor | 0.112 |
| Decanoyl L-Carnitine | PPARA | Nuclear receptor | 0.112 |
| Decanoyl L-Carnitine | ALOX12 | Enzyme | 0.112 |
| Decanoyl L-Carnitine | TBXAS1 | Cytochrome P450 | 0.112 |
| Decanoyl L-Carnitine | P2RY12 | Family A G protein-coupled receptor | 0.112 |
| Decanoyl L-Carnitine | PPARD | Nuclear receptor | 0.112 |
| Decanoyl L-Carnitine | BCHE | Hydrolase | 0.112 |
| Decanoyl L-Carnitine | ITGAV | Membrane receptor | 0.112 |
| Decanoyl L-Carnitine | ITGB3 | Membrane receptor | 0.112 |
| Decanoyl L-Carnitine | ITGA2B | Membrane receptor | 0.112 |
| Decanoyl L-Carnitine | ITGB3 | Membrane receptor | 0.112 |
| Decanoyl L-Carnitine | AGTR1 | Family A G protein-coupled receptor | 0.112 |
| Decanoyl L-Carnitine | MMEL1 | Hydrolase | 0.112 |
| Decanoyl L-Carnitine | LTB4R | Family A G protein-coupled receptor | 0.112 |
| Decanoyl L-Carnitine | FFAR2 | Family A G protein-coupled receptor | 0.112 |
| Decanoyl L-Carnitine | CTSA | Protease | 0.112 |
| Decanoyl L-Carnitine | ABHD12 | Enzyme | 0.112 |
| Decanoyl L-Carnitine | ABHD16A | Enzyme | 0.112 |
| Decanoyl L-Carnitine | ITGB1 | Membrane receptor | 0.112 |
| Decanoyl L-Carnitine | ITGA4 | Membrane receptor | 0.112 |
| Decanoyl L-Carnitine | MMP3 | Protease | 0.112 |
| Decanoyl L-Carnitine | MMP2 | Protease | 0.112 |
| Decanoyl L-Carnitine | ENPP2 | Enzyme | 0.112 |
| Decanoyl L-Carnitine | ELANE | Protease | 0.112 |
| Decanoyl L-Carnitine | PDE4A | Phosphodiesterase | 0.112 |
| Decanoyl L-Carnitine | PDE4B | Phosphodiesterase | 0.112 |
| Decanoyl L-Carnitine | PDE4D | Phosphodiesterase | 0.112 |
| Decanoyl L-Carnitine | FFAR1 | Family A G protein-coupled receptor | 0.112 |
| Decanoyl L-Carnitine | ALOX5AP | Other cytosolic protein | 0.112 |
| Decanoyl L-Carnitine | PLA2G4A | Enzyme | 0.112 |
| Decanoyl L-Carnitine | MMP12 | Protease | 0.112 |
| Decanoyl L-Carnitine | MMP8 | Protease | 0.112 |
| Decanoyl L-Carnitine | CTSH | Protease | 0.112 |
| Decanoyl L-Carnitine | VCP | Primary active transporter | 0.112 |
| Decanoyl L-Carnitine | ADAMTS5 | Protease | 0.112 |
| Decanoyl L-Carnitine | ADAMTS4 | Protease | 0.112 |
| Decanoyl L-Carnitine | MDM2 | Other nuclear protein | 0.112 |
| Decanoyl L-Carnitine | RORC | Nuclear receptor | 0.112 |
| Decanoyl L-Carnitine | PTGIR | Family A G protein-coupled receptor | 0.112 |
| Decanoyl L-Carnitine | MMP13 | Protease | 0.112 |
| Decanoyl L-Carnitine | CCKBR | Family A G protein-coupled receptor | 0.112 |
| Decanoyl L-Carnitine | TRPA1 | Voltage-gated ion channel | 0.112 |
| Decanoyl L-Carnitine | GCGR | Family B G protein-coupled receptor | 0.112 |
| Decanoyl L-Carnitine | PDGFRB | Kinase | 0.112 |
| Decanoyl L-Carnitine | FNTA | Enzyme | 0.112 |
| Decanoyl L-Carnitine | FNTB | Enzyme | 0.112 |
| Decanoyl L-Carnitine | CDC25C | Phosphatase | 0.112 |
| Decanoyl L-Carnitine | SRC | Kinase | 0.112 |
| Decanoyl L-Carnitine | KDR | Kinase | 0.112 |
| Decanoyl L-Carnitine | FGFR1 | Kinase | 0.112 |
| Decanoyl L-Carnitine | CDC25B | Phosphatase | 0.112 |
| Decanoyl L-Carnitine | DAGLA | Enzyme | 0.112 |
| Decanoyl L-Carnitine | CASP3 | Protease | 0.112 |
| Decanoyl L-Carnitine | ABHD6 | Enzyme | 0.112 |
| Decanoyl L-Carnitine | TRPM8 | Voltage-gated ion channel | 0.112 |
| Decanoyl L-Carnitine | FABP4 | Fatty acid binding protein family | 0.112 |
| Decanoyl L-Carnitine | ITGB5 | Membrane receptor | 0.112 |
| Decanoyl L-Carnitine | ITGAV | Membrane receptor | 0.112 |
| Decanoyl L-Carnitine | ADORA1 | Family A G protein-coupled receptor | 0.112 |
| Decanoyl L-Carnitine | CA1 | Lyase | 0.112 |
| Decanoyl L-Carnitine | ITGA4 | Membrane receptor | 0.112 |
| Decanoyl L-Carnitine | CA12 | Lyase | 0.112 |
| Decanoyl L-Carnitine | CA9 | Lyase | 0.112 |
| Decanoyl L-Carnitine | MMP9 | Protease | 0.112 |
| Decanoyl L-Carnitine | PLA2G2A | Enzyme | 0.112 |
| Decanoyl L-Carnitine | SIRT1 | Eraser | 0.112 |
| Decanoyl L-Carnitine | ADORA2A | Family A G protein-coupled receptor | 0.112 |
| Decanoyl L-Carnitine | CASP1 | Protease | 0.112 |
| Decanoyl L-Carnitine | AKR1B1 | Enzyme | 0.112 |
| Decanoyl L-Carnitine | PFKFB3 | Enzyme | 0.112 |
| Decanoyl L-Carnitine | SYK | Kinase | 0.112 |
| Decanoyl L-Carnitine | NTRK1 | Kinase | 0.112 |
| Decanoyl L-Carnitine | PTPRF | Membrane receptor | 0.112 |
| Decanoyl L-Carnitine | PTPN2 | Phosphatase | 0.112 |
| Decanoyl L-Carnitine | ITGB7 | Membrane receptor | 0.112 |
| Decanoyl L-Carnitine | ITGA4 | Membrane receptor | 0.112 |
| Decanoyl L-Carnitine | ADAM17 | Protease | 0.112 |
| Decanoyl L-Carnitine | MMP14 | Protease | 0.112 |
| Decanoyl L-Carnitine | MMP10 | Protease | 0.112 |
| Decanoyl L-Carnitine | CXCR2 | Family A G protein-coupled receptor | 0.112 |
| Decanoyl L-Carnitine | RARA | Nuclear receptor | 0.112 |
| Decanoyl L-Carnitine | RXRA | Nuclear receptor | 0.112 |
| Decanoyl L-Carnitine | CPA1 | Protease | 0.112 |
| Decanoyl L-Carnitine | EDNRA | Family A G protein-coupled receptor | 0.112 |
| Decanoyl L-Carnitine | SLC10A2 | Electrochemical transporter | 0.112 |
| Decanoyl L-Carnitine | ACE2 | Protease | 0.112 |
| Decanoyl L-Carnitine | AURKA | Kinase | 0.112 |
| Decanoyl L-Carnitine | FFAR4 | Family A G protein-coupled receptor | 0.112 |
| Decanoyl L-Carnitine | PGGT1B | Enzyme | 0.112 |
| Decanoyl L-Carnitine | FNTA | Enzyme | 0.112 |
| Decanoyl L-Carnitine | CCR1 | Family A G protein-coupled receptor | 0.112 |
| Decanoyl L-Carnitine | HIF1A | Transcription factor | 0.112 |
| Decanoyl L-Carnitine | GCG | Unclassified protein | 0.112 |
| UNII-0TM46496W4 | CYP11B1 | Cytochrome P450 | 0.112 |
| UNII-0TM46496W4 | VDR | Nuclear receptor | 0.112 |
| UNII-0TM46496W4 | CYP11B2 | Cytochrome P450 | 0.112 |
| UNII-0TM46496W4 | MAPK14 | Kinase | 0.112 |
| UNII-0TM46496W4 | PIM1 | Kinase | 0.112 |
| UNII-0TM46496W4 | PYGL | Enzyme | 0.112 |
| UNII-0TM46496W4 | PIM3 | Kinase | 0.112 |
| UNII-0TM46496W4 | F2RL3 | Family A G protein-coupled receptor | 0.112 |
| UNII-0TM46496W4 | OPRM1 | Family A G protein-coupled receptor | 0.112 |
| UNII-0TM46496W4 | MAP3K14 | Kinase | 0.112 |
| UNII-0TM46496W4 | CA2 | Lyase | 0.112 |
| UNII-0TM46496W4 | CA1 | Lyase | 0.112 |
| UNII-0TM46496W4 | JAK1 | Kinase | 0.112 |
| UNII-0TM46496W4 | JAK2 | Kinase | 0.112 |
| UNII-0TM46496W4 | CA7 | Lyase | 0.112 |
| UNII-0TM46496W4 | CA3 | Lyase | 0.112 |
| UNII-0TM46496W4 | CA6 | Lyase | 0.112 |
| UNII-0TM46496W4 | CA12 | Lyase | 0.112 |
| UNII-0TM46496W4 | CA14 | Lyase | 0.112 |
| UNII-0TM46496W4 | CA9 | Lyase | 0.112 |
| UNII-0TM46496W4 | CA4 | Lyase | 0.112 |
| UNII-0TM46496W4 | CA13 | Lyase | 0.112 |
| UNII-0TM46496W4 | CA5B | Lyase | 0.112 |
| UNII-0TM46496W4 | ICMT | Enzyme | 0.112 |
| UNII-0TM46496W4 | HMGCR | Oxidoreductase | 0.112 |
| UNII-0TM46496W4 | MTNR1A | Family A G protein-coupled receptor | 0.112 |
| UNII-0TM46496W4 | JAK3 | Kinase | 0.112 |
| UNII-0TM46496W4 | TYK2 | Kinase | 0.112 |
| UNII-0TM46496W4 | HMOX1 | Enzyme | 0.112 |
| UNII-0TM46496W4 | ABCC9 | Primary active transporter | 0.112 |
| UNII-0TM46496W4 | CCNB3 | Other cytosolic protein | 0.112 |
| UNII-0TM46496W4 | CDK1 | Other cytosolic protein | 0.112 |
| UNII-0TM46496W4 | CCNB1 | Other cytosolic protein | 0.112 |
| UNII-0TM46496W4 | CCNB2 | Other cytosolic protein | 0.112 |
| UNII-0TM46496W4 | GSK3B | Kinase | 0.112 |
| UNII-0TM46496W4 | CDK1 | Kinase | 0.112 |
| UNII-0TM46496W4 | AR | Nuclear receptor | 0.112 |
| UNII-0TM46496W4 | JAK3 | Kinase | 0.112 |
| UNII-0TM46496W4 | JAK1 | Kinase | 0.112 |
| UNII-0TM46496W4 | HSD17B2 | Enzyme | 0.112 |
| UNII-0TM46496W4 | ADA | Hydrolase | 0.112 |
| UNII-0TM46496W4 | CYP51A1 | Cytochrome P450 | 0.112 |
| UNII-0TM46496W4 | PDE10A | Phosphodiesterase | 0.112 |
| UNII-0TM46496W4 | PGR | Nuclear receptor | 0.112 |
| UNII-0TM46496W4 | TRPV1 | Voltage-gated ion channel | 0.112 |
| UNII-0TM46496W4 | LRRK2 | Kinase | 0.112 |
| UNII-0TM46496W4 | SIGMAR1 | Membrane receptor | 0.112 |
| UNII-0TM46496W4 | CYP19A1 | Cytochrome P450 | 0.112 |
| UNII-0TM46496W4 | AMPD3 | Enzyme | 0.112 |
| UNII-0TM46496W4 | MKNK1 | Kinase | 0.112 |
| UNII-0TM46496W4 | CDC7 | Kinase | 0.112 |
| UNII-0TM46496W4 | CYP17A1 | Cytochrome P450 | 0.112 |
| UNII-0TM46496W4 | ADAM17 | Protease | 0.112 |
| UNII-0TM46496W4 | NPY5R | Family A G protein-coupled receptor | 0.112 |
| UNII-0TM46496W4 | EGFR | Kinase | 0.112 |
| UNII-0TM46496W4 | TTK | Kinase | 0.112 |
| UNII-0TM46496W4 | PIM2 | Kinase | 0.112 |
| UNII-0TM46496W4 | KCNJ11 | Primary active transporter | 0.112 |
| UNII-0TM46496W4 | ABCC9 | Primary active transporter | 0.112 |
| UNII-0TM46496W4 | PDE7A | Phosphodiesterase | 0.112 |
| UNII-0TM46496W4 | HSD11B1 | Enzyme | 0.112 |
| UNII-0TM46496W4 | CCR5 | Family A G protein-coupled receptor | 0.112 |
| UNII-0TM46496W4 | BRD4 | Reader | 0.112 |
| UNII-0TM46496W4 | CHRM4 | Family A G protein-coupled receptor | 0.112 |
| UNII-0TM46496W4 | CHRM5 | Family A G protein-coupled receptor | 0.112 |
| UNII-0TM46496W4 | CHRM2 | Family A G protein-coupled receptor | 0.112 |
| UNII-0TM46496W4 | CHRM1 | Family A G protein-coupled receptor | 0.112 |
| UNII-0TM46496W4 | CYP2C9 | Cytochrome P450 | 0.112 |
| UNII-0TM46496W4 | AOC3 | Enzyme | 0.112 |
| UNII-0TM46496W4 | CYP2C19 | Cytochrome P450 | 0.112 |
| UNII-0TM46496W4 | CREBBP | Writer | 0.112 |
| UNII-0TM46496W4 | MAOA | Oxidoreductase | 0.112 |
| UNII-0TM46496W4 | TYMS | Transferase | 0.112 |
| UNII-0TM46496W4 | MAP2K1 | Kinase | 0.112 |
| UNII-0TM46496W4 | MAPK1 | Kinase | 0.112 |
| UNII-0TM46496W4 | LTA4H | Protease | 0.112 |
| UNII-0TM46496W4 | ACPP | Enzyme | 0.112 |
| UNII-0TM46496W4 | CSNK1D | Kinase | 0.112 |
| UNII-0TM46496W4 | CHEK1 | Kinase | 0.112 |
| UNII-0TM46496W4 | CSNK1E | Kinase | 0.112 |
| UNII-0TM46496W4 | CA5A | Lyase | 0.112 |
| UNII-0TM46496W4 | CDK5R1 | Kinase | 0.112 |
| UNII-0TM46496W4 | CDK5 | Kinase | 0.112 |
| UNII-0TM46496W4 | PARP1 | Enzyme | 0.112 |
| UNII-0TM46496W4 | NR3C2 | Nuclear receptor | 0.112 |
| UNII-0TM46496W4 | EDNRA | Family A G protein-coupled receptor | 0.112 |
| UNII-0TM46496W4 | KCNMA1 | Voltage-gated ion channel | 0.112 |
| UNII-0TM46496W4 | PTGES | Enzyme | 0.112 |
| UNII-0TM46496W4 | PRKCD | Kinase | 0.112 |
| UNII-0TM46496W4 | TOP2A | Isomerase | 0.112 |
| UNII-0TM46496W4 | GABRB3 | Ligand-gated ion channel | 0.112 |
| UNII-0TM46496W4 | GABRG2 | Ligand-gated ion channel | 0.112 |
| UNII-0TM46496W4 | GABRA5 | Ligand-gated ion channel | 0.112 |
| UNII-0TM46496W4 | ADORA2A | Family A G protein-coupled receptor | 0.112 |
| UNII-0TM46496W4 | AURKA | Kinase | 0.112 |
| UNII-0TM46496W4 | CNR1 | Family A G protein-coupled receptor | 0.112 |
| UNII-0TM46496W4 | GABRB3 | Ligand-gated ion channel | 0.112 |
| UNII-0TM46496W4 | GABRA3 | Ligand-gated ion channel | 0.112 |
| UNII-0TM46496W4 | GABRG2 | Ligand-gated ion channel | 0.112 |
| UNII-0TM46496W4 | GABRB3 | Ligand-gated ion channel | 0.112 |
| UNII-0TM46496W4 | GABRG2 | Ligand-gated ion channel | 0.112 |
| UNII-0TM46496W4 | GABRA1 | Ligand-gated ion channel | 0.112 |
| UNII-0TM46496W4 | GABRA2 | Ligand-gated ion channel | 0.112 |
| UNII-0TM46496W4 | GABRB3 | Ligand-gated ion channel | 0.112 |
| UNII-0TM46496W4 | GABRG2 | Ligand-gated ion channel | 0.112 |
| UNII-0TM46496W4 | CHRNA3 | Ligand-gated ion channel | 0.112 |
| UNII-0TM46496W4 | CHRNB4 | Ligand-gated ion channel | 0.112 |
| UNII-0TM46496W4 | COMT | Transferase | 0.112 |
| UNII-0TM46496W4 | PTAFR | Family A G protein-coupled receptor | 0.112 |
| UNII-0TM46496W4 | ERBB2 | Kinase | 0.112 |
| UNII-0TM46496W4 | NR3C1 | Nuclear receptor | 0.112 |
| UNII-0TM46496W4 | ERBB4 | Kinase | 0.112 |
| UNII-0TM46496W4 | KCNE1 | Voltage-gated ion channel | 0.112 |
| UNII-0TM46496W4 | KCNQ1 | Voltage-gated ion channel | 0.112 |
| UNII-0TM46496W4 | MPO | Enzyme | 0.112 |
| UNII-0TM46496W4 | ROCK2 | Kinase | 0.112 |
| Carnitine C10:0 | SLC25A20 | Electrochemical transporter | 0.996 |
| Carnitine C10:0 | NR1H4 | Nuclear receptor | 0.499 |
| Carnitine C10:0 | CPT1A | Enzyme | 0.112 |
| Carnitine C10:0 | OXER1 | Family A G protein-coupled receptor | 0.112 |
| Carnitine C10:0 | PTGER1 | Family A G protein-coupled receptor | 0.112 |
| Carnitine C10:0 | PTGER2 | Family A G protein-coupled receptor | 0.112 |
| Carnitine C10:0 | PTGDR | Family A G protein-coupled receptor | 0.112 |
| Carnitine C10:0 | PTGER4 | Family A G protein-coupled receptor | 0.112 |
| Carnitine C10:0 | CPT1B | Group translocator | 0.112 |
| Carnitine C10:0 | TBXA2R | Family A G protein-coupled receptor | 0.112 |
| Carnitine C10:0 | PTGDR2 | Family A G protein-coupled receptor | 0.112 |
| Carnitine C10:0 | NR4A1 | Nuclear receptor | 0.112 |
| Carnitine C10:0 | ESR2 | Nuclear receptor | 0.112 |
| Carnitine C10:0 | CNR1 | Family A G protein-coupled receptor | 0.112 |
| Carnitine C10:0 | PPARG | Nuclear receptor | 0.112 |
| Carnitine C10:0 | PPARA | Nuclear receptor | 0.112 |
| Carnitine C10:0 | ALOX12 | Enzyme | 0.112 |
| Carnitine C10:0 | TBXAS1 | Cytochrome P450 | 0.112 |
| Carnitine C10:0 | P2RY12 | Family A G protein-coupled receptor | 0.112 |
| Carnitine C10:0 | PPARD | Nuclear receptor | 0.112 |
| Carnitine C10:0 | BCHE | Hydrolase | 0.112 |
| Carnitine C10:0 | ITGAV | Membrane receptor | 0.112 |
| Carnitine C10:0 | ITGB3 | Membrane receptor | 0.112 |
| Carnitine C10:0 | ITGA2B | Membrane receptor | 0.112 |
| Carnitine C10:0 | ITGB3 | Membrane receptor | 0.112 |
| Carnitine C10:0 | AGTR1 | Family A G protein-coupled receptor | 0.112 |
| Carnitine C10:0 | MMEL1 | Hydrolase | 0.112 |
| Carnitine C10:0 | LTB4R | Family A G protein-coupled receptor | 0.112 |
| Carnitine C10:0 | FFAR2 | Family A G protein-coupled receptor | 0.112 |
| Carnitine C10:0 | CTSA | Protease | 0.112 |
| Carnitine C10:0 | ABHD12 | Enzyme | 0.112 |
| Carnitine C10:0 | ABHD16A | Enzyme | 0.112 |
| Carnitine C10:0 | ITGB1 | Membrane receptor | 0.112 |
| Carnitine C10:0 | ITGA4 | Membrane receptor | 0.112 |
| Carnitine C10:0 | MMP3 | Protease | 0.112 |
| Carnitine C10:0 | MMP2 | Protease | 0.112 |
| Carnitine C10:0 | ENPP2 | Enzyme | 0.112 |
| Carnitine C10:0 | ELANE | Protease | 0.112 |
| Carnitine C10:0 | PDE4A | Phosphodiesterase | 0.112 |
| Carnitine C10:0 | PDE4B | Phosphodiesterase | 0.112 |
| Carnitine C10:0 | PDE4D | Phosphodiesterase | 0.112 |
| Carnitine C10:0 | FFAR1 | Family A G protein-coupled receptor | 0.112 |
| Carnitine C10:0 | ALOX5AP | Other cytosolic protein | 0.112 |
| Carnitine C10:0 | PLA2G4A | Enzyme | 0.112 |
| Carnitine C10:0 | MMP12 | Protease | 0.112 |
| Carnitine C10:0 | MMP8 | Protease | 0.112 |
| Carnitine C10:0 | CTSH | Protease | 0.112 |
| Carnitine C10:0 | VCP | Primary active transporter | 0.112 |
| Carnitine C10:0 | ADAMTS5 | Protease | 0.112 |
| Carnitine C10:0 | ADAMTS4 | Protease | 0.112 |
| Carnitine C10:0 | MDM2 | Other nuclear protein | 0.112 |
| Carnitine C10:0 | RORC | Nuclear receptor | 0.112 |
| Carnitine C10:0 | PTGIR | Family A G protein-coupled receptor | 0.112 |
| Carnitine C10:0 | MMP13 | Protease | 0.112 |
| Carnitine C10:0 | CCKBR | Family A G protein-coupled receptor | 0.112 |
| Carnitine C10:0 | TRPA1 | Voltage-gated ion channel | 0.112 |
| Carnitine C10:0 | GCGR | Family B G protein-coupled receptor | 0.112 |
| Carnitine C10:0 | PDGFRB | Kinase | 0.112 |
| Carnitine C10:0 | FNTA | Enzyme | 0.112 |
| Carnitine C10:0 | FNTB | Enzyme | 0.112 |
| Carnitine C10:0 | CDC25C | Phosphatase | 0.112 |
| Carnitine C10:0 | SRC | Kinase | 0.112 |
| Carnitine C10:0 | KDR | Kinase | 0.112 |
| Carnitine C10:0 | FGFR1 | Kinase | 0.112 |
| Carnitine C10:0 | CDC25B | Phosphatase | 0.112 |
| Carnitine C10:0 | DAGLA | Enzyme | 0.112 |
| Carnitine C10:0 | CASP3 | Protease | 0.112 |
| Carnitine C10:0 | ABHD6 | Enzyme | 0.112 |
| Carnitine C10:0 | TRPM8 | Voltage-gated ion channel | 0.112 |
| Carnitine C10:0 | FABP4 | Fatty acid binding protein family | 0.112 |
| Carnitine C10:0 | ITGB5 | Membrane receptor | 0.112 |
| Carnitine C10:0 | ITGAV | Membrane receptor | 0.112 |
| Carnitine C10:0 | ADORA1 | Family A G protein-coupled receptor | 0.112 |
| Carnitine C10:0 | CA1 | Lyase | 0.112 |
| Carnitine C10:0 | ITGA4 | Membrane receptor | 0.112 |
| Carnitine C10:0 | CA12 | Lyase | 0.112 |
| Carnitine C10:0 | CA9 | Lyase | 0.112 |
| Carnitine C10:0 | MMP9 | Protease | 0.112 |
| Carnitine C10:0 | PLA2G2A | Enzyme | 0.112 |
| Carnitine C10:0 | SIRT1 | Eraser | 0.112 |
| Carnitine C10:0 | ADORA2A | Family A G protein-coupled receptor | 0.112 |
| Carnitine C10:0 | CASP1 | Protease | 0.112 |
| Carnitine C10:0 | AKR1B1 | Enzyme | 0.112 |
| Carnitine C10:0 | PFKFB3 | Enzyme | 0.112 |
| Carnitine C10:0 | SYK | Kinase | 0.112 |
| Carnitine C10:0 | NTRK1 | Kinase | 0.112 |
| Carnitine C10:0 | PTPRF | Membrane receptor | 0.112 |
| Carnitine C10:0 | PTPN2 | Phosphatase | 0.112 |
| Carnitine C10:0 | ITGB7 | Membrane receptor | 0.112 |
| Carnitine C10:0 | ITGA4 | Membrane receptor | 0.112 |
| Carnitine C10:0 | ADAM17 | Protease | 0.112 |
| Carnitine C10:0 | MMP14 | Protease | 0.112 |
| Carnitine C10:0 | MMP10 | Protease | 0.112 |
| Carnitine C10:0 | CXCR2 | Family A G protein-coupled receptor | 0.112 |
| Carnitine C10:0 | RARA | Nuclear receptor | 0.112 |
| Carnitine C10:0 | RXRA | Nuclear receptor | 0.112 |
| Carnitine C10:0 | CPA1 | Protease | 0.112 |
| Carnitine C10:0 | EDNRA | Family A G protein-coupled receptor | 0.112 |
| Carnitine C10:0 | SLC10A2 | Electrochemical transporter | 0.112 |
| Carnitine C10:0 | ACE2 | Protease | 0.112 |
| Carnitine C10:0 | AURKA | Kinase | 0.112 |
| Carnitine C10:0 | FFAR4 | Family A G protein-coupled receptor | 0.112 |
| Carnitine C10:0 | PGGT1B | Enzyme | 0.112 |
| Carnitine C10:0 | FNTA | Enzyme | 0.112 |
| Carnitine C10:0 | CCR1 | Family A G protein-coupled receptor | 0.112 |
| Carnitine C10:0 | HIF1A | Transcription factor | 0.112 |
| Carnitine C10:0 | GCG | Unclassified protein | 0.112 |
| Carnitine C12:0 | SLC25A20 | Electrochemical transporter | 0.662 |
| Carnitine C12:0 | NR1H4 | Nuclear receptor | 0.315 |
| Carnitine C12:0 | CPT1A | Enzyme | 0.105 |
| Carnitine C12:0 | CPT1B | Group translocator | 0.105 |
| Carnitine C12:0 | PTGDR2 | Family A G protein-coupled receptor | 0.105 |
| Carnitine C12:0 | P2RY12 | Family A G protein-coupled receptor | 0.105 |
| Carnitine C12:0 | TBXA2R | Family A G protein-coupled receptor | 0.105 |
| Carnitine C12:0 | MDM2 | Other nuclear protein | 0.105 |
| Carnitine C12:0 | PTGER2 | Family A G protein-coupled receptor | 0.105 |
| Carnitine C12:0 | ITGB1 | Membrane receptor | 0.105 |
| Carnitine C12:0 | ITGA4 | Membrane receptor | 0.105 |
| Carnitine C12:0 | TBXAS1 | Cytochrome P450 | 0.105 |
| Carnitine C12:0 | PTGER4 | Family A G protein-coupled receptor | 0.105 |
| Carnitine C12:0 | PTGDR | Family A G protein-coupled receptor | 0.105 |
| Carnitine C12:0 | PPARG | Nuclear receptor | 0.105 |
| Carnitine C12:0 | ALOX12 | Enzyme | 0.105 |
| Carnitine C12:0 | PPARA | Nuclear receptor | 0.105 |
| Carnitine C12:0 | ALOX5AP | Other cytosolic protein | 0.105 |
| Carnitine C12:0 | BCHE | Hydrolase | 0.105 |
| Carnitine C12:0 | GCGR | Family B G protein-coupled receptor | 0.105 |
| Carnitine C12:0 | ITGAV | Membrane receptor | 0.105 |
| Carnitine C12:0 | ITGB3 | Membrane receptor | 0.105 |
| Carnitine C12:0 | ITGA2B | Membrane receptor | 0.105 |
| Carnitine C12:0 | ITGB3 | Membrane receptor | 0.105 |
| Carnitine C12:0 | ITGB5 | Membrane receptor | 0.105 |
| Carnitine C12:0 | ITGAV | Membrane receptor | 0.105 |
| Carnitine C12:0 | FFAR1 | Family A G protein-coupled receptor | 0.105 |
| Carnitine C12:0 | PTGER1 | Family A G protein-coupled receptor | 0.105 |
| Carnitine C12:0 | PTGIR | Family A G protein-coupled receptor | 0.105 |
| Carnitine C12:0 | ABHD12 | Enzyme | 0.105 |
| Carnitine C12:0 | DAGLB | Enzyme | 0.105 |
| Carnitine C12:0 | ABHD16A | Enzyme | 0.105 |
| Carnitine C12:0 | MMP12 | Protease | 0.105 |
| Carnitine C12:0 | MMP13 | Protease | 0.105 |
| Carnitine C12:0 | MMP2 | Protease | 0.105 |
| Carnitine C12:0 | MMP8 | Protease | 0.105 |
| Carnitine C12:0 | SOAT1 | Enzyme | 0.105 |
| Carnitine C12:0 | SOAT2 | Enzyme | 0.105 |
| Carnitine C12:0 | GCG | Unclassified protein | 0.105 |
| Carnitine C12:0 | ITGAL | Membrane receptor | 0.105 |
| Carnitine C12:0 | ICAM1 | Membrane receptor | 0.105 |
| Carnitine C12:0 | ITGB2 | Membrane receptor | 0.105 |
| Carnitine C12:0 | ADORA2A | Family A G protein-coupled receptor | 0.105 |
| Carnitine C12:0 | DGAT1 | Enzyme | 0.105 |
| Carnitine C12:0 | PLA2G4A | Enzyme | 0.105 |
| Carnitine C12:0 | GIPR | Family B G protein-coupled receptor | 0.105 |
| Carnitine C12:0 | TRPM8 | Voltage-gated ion channel | 0.105 |
| Carnitine C12:0 | CTSH | Protease | 0.105 |
| Carnitine C12:0 | ITGAL | Adhesion | 0.105 |
| Carnitine C12:0 | PPARD | Nuclear receptor | 0.105 |
| Carnitine C12:0 | VCP | Primary active transporter | 0.105 |
| Carnitine C12:0 | CYSLTR1 | Family A G protein-coupled receptor | 0.105 |
| Carnitine C12:0 | PDE5A | Phosphodiesterase | 0.105 |
| Carnitine C12:0 | ABCC1 | Primary active transporter | 0.105 |
| Carnitine C12:0 | LTB4R | Family A G protein-coupled receptor | 0.105 |
| Carnitine C12:0 | PTPN11 | Phosphatase | 0.105 |
| Carnitine C12:0 | CDC25C | Phosphatase | 0.105 |
| Carnitine C12:0 | CDC25B | Phosphatase | 0.105 |
| Carnitine C12:0 | HDAC1 | Eraser | 0.105 |
| Carnitine C12:0 | MMP9 | Protease | 0.105 |
| Carnitine C12:0 | ITGA4 | Membrane receptor | 0.105 |
| Carnitine C12:0 | CCKBR | Family A G protein-coupled receptor | 0.105 |
| Carnitine C12:0 | PTPN6 | Phosphatase | 0.105 |
| Carnitine C12:0 | PI4KB | Enzyme | 0.105 |
| Carnitine C12:0 | PI4KA | Enzyme | 0.105 |
| Carnitine C12:0 | PTPN2 | Phosphatase | 0.105 |
| Carnitine C12:0 | NTSR1 | Family A G protein-coupled receptor | 0.105 |
| Carnitine C12:0 | CASP1 | Protease | 0.105 |
| Carnitine C12:0 | PDE4B | Phosphodiesterase | 0.105 |
| Carnitine C12:0 | PDE4D | Phosphodiesterase | 0.105 |
| Carnitine C12:0 | MMP1 | Protease | 0.105 |
| Carnitine C12:0 | CNR1 | Family A G protein-coupled receptor | 0.105 |
| Carnitine C12:0 | ADAMTS5 | Protease | 0.105 |
| Carnitine C12:0 | ADAMTS4 | Protease | 0.105 |
| Carnitine C12:0 | MAPK14 | Kinase | 0.105 |
| Carnitine C12:0 | MAPK10 | Kinase | 0.105 |
| Carnitine C12:0 | MMP3 | Protease | 0.105 |
| Carnitine C12:0 | SIRT1 | Eraser | 0.105 |
| Carnitine C12:0 | ADORA1 | Family A G protein-coupled receptor | 0.105 |
| Carnitine C12:0 | CPB1 | Protease | 0.105 |
| Carnitine C12:0 | ADORA2B | Family A G protein-coupled receptor | 0.105 |
| Carnitine C12:0 | MMP14 | Protease | 0.105 |
| Carnitine C12:0 | PLA2G2C | Enzyme | 0.105 |
| Carnitine C12:0 | CASP3 | Protease | 0.105 |
| Carnitine C12:0 | FNTA | Enzyme | 0.105 |
| Carnitine C12:0 | FNTB | Enzyme | 0.105 |
| Carnitine C12:0 | ITGAV | Membrane receptor | 0.105 |
| Carnitine C12:0 | ITGB1 | Membrane receptor | 0.105 |
| Carnitine C12:0 | PTGFR | Family A G protein-coupled receptor | 0.105 |
| Carnitine C12:0 | PDE10A | Phosphodiesterase | 0.105 |
| Carnitine C12:0 | PLA2G2A | Enzyme | 0.105 |
| Carnitine C12:0 | STAT3 | Transcription factor | 0.105 |
| Carnitine C12:0 | PLA2G10 | Enzyme | 0.105 |
| Carnitine C12:0 | SRD5A2 | Oxidoreductase | 0.105 |
| Carnitine C12:0 | PDGFRB | Kinase | 0.105 |
| Carnitine C12:0 | SRC | Kinase | 0.105 |
| Carnitine C12:0 | KDR | Kinase | 0.105 |
| Carnitine C12:0 | FGFR1 | Kinase | 0.105 |
| Carnitine C12:0 | BACE1 | Protease | 0.105 |
| Carnitine C12:0 | CHRM4 | Family A G protein-coupled receptor | 0.105 |
| Carnitine C12:0 | HTR2B | Family A G protein-coupled receptor | 0.105 |
| Carnitine C12:0 | CHRM5 | Family A G protein-coupled receptor | 0.105 |
| Carnitine C12:0 | CHRM2 | Family A G protein-coupled receptor | 0.105 |
| Carnitine C12:0 | CHRM1 | Family A G protein-coupled receptor | 0.105 |
| Carnitine C12:0 | HTR2C | Family A G protein-coupled receptor | 0.105 |
| Carnitine C12:0 | DRD3 | Family A G protein-coupled receptor | 0.105 |
| Carnitine C12:0 | CHRM3 | Family A G protein-coupled receptor | 0.105 |
| Carnitine C12:0 | SIGMAR1 | Membrane receptor | 0.105 |
| Octanoylcarnitine | SLC25A20 | Electrochemical transporter | 0.592 |
| Octanoylcarnitine | NR1H4 | Nuclear receptor | 0.420 |
| Octanoylcarnitine | CPT1A | Enzyme | 0.101 |
| Octanoylcarnitine | ITGB1 | Membrane receptor | 0.101 |
| Octanoylcarnitine | ITGA4 | Membrane receptor | 0.101 |
| Octanoylcarnitine | CPT1B | Group translocator | 0.101 |
| Octanoylcarnitine | MDM2 | Other nuclear protein | 0.101 |
| Octanoylcarnitine | CASP1 | Protease | 0.101 |
| Octanoylcarnitine | BCHE | Hydrolase | 0.101 |
| Octanoylcarnitine | CCKAR | Family A G protein-coupled receptor | 0.101 |
| Octanoylcarnitine | FFAR1 | Family A G protein-coupled receptor | 0.101 |
| Octanoylcarnitine | ALOX15 | Enzyme | 0.101 |
| Octanoylcarnitine | ALOX12 | Enzyme | 0.101 |
| Octanoylcarnitine | PTGDR2 | Family A G protein-coupled receptor | 0.101 |
| Octanoylcarnitine | ABHD12 | Enzyme | 0.101 |
| Octanoylcarnitine | ABHD16A | Enzyme | 0.101 |
| Octanoylcarnitine | PTGER2 | Family A G protein-coupled receptor | 0.101 |
| Octanoylcarnitine | HSD11B2 | Enzyme | 0.101 |
| Octanoylcarnitine | TBXA2R | Family A G protein-coupled receptor | 0.101 |
| Octanoylcarnitine | CYP26A1 | Cytochrome P450 | 0.101 |
| Octanoylcarnitine | CTSB | Protease | 0.101 |
| Octanoylcarnitine | DUSP3 | Phosphatase | 0.101 |
| Octanoylcarnitine | VCP | Primary active transporter | 0.101 |
| Octanoylcarnitine | PTGDR | Family A G protein-coupled receptor | 0.101 |
| Octanoylcarnitine | OXER1 | Family A G protein-coupled receptor | 0.101 |
| Octanoylcarnitine | PPARD | Nuclear receptor | 0.101 |
| Octanoylcarnitine | TBXAS1 | Cytochrome P450 | 0.101 |
| Octanoylcarnitine | CTSH | Protease | 0.101 |
| Octanoylcarnitine | AGTR1 | Family A G protein-coupled receptor | 0.101 |
| Octanoylcarnitine | PDE4B | Phosphodiesterase | 0.101 |
| Octanoylcarnitine | PLA2G10 | Enzyme | 0.101 |
| Octanoylcarnitine | GZMB | Protease | 0.101 |
| Octanoylcarnitine | ITGAV | Membrane receptor | 0.101 |
| Octanoylcarnitine | ITGB3 | Membrane receptor | 0.101 |
| Octanoylcarnitine | DAGLA | Enzyme | 0.101 |
| Octanoylcarnitine | MMP1 | Protease | 0.101 |
| Octanoylcarnitine | MMP2 | Protease | 0.101 |
| Octanoylcarnitine | PPARG | Nuclear receptor | 0.101 |
| Octanoylcarnitine | CCKBR | Family A G protein-coupled receptor | 0.101 |
| Octanoylcarnitine | MMEL1 | Hydrolase | 0.101 |
| Octanoylcarnitine | CTSA | Protease | 0.101 |
| Octanoylcarnitine | PTGER1 | Family A G protein-coupled receptor | 0.101 |
| Octanoylcarnitine | PDE4A | Phosphodiesterase | 0.101 |
| Octanoylcarnitine | PDE4D | Phosphodiesterase | 0.101 |
| Octanoylcarnitine | PPARA | Nuclear receptor | 0.101 |
| Octanoylcarnitine | LTB4R | Family A G protein-coupled receptor | 0.101 |
| Octanoylcarnitine | CNR1 | Family A G protein-coupled receptor | 0.101 |
| Octanoylcarnitine | ADORA1 | Family A G protein-coupled receptor | 0.101 |
| Octanoylcarnitine | EDNRA | Family A G protein-coupled receptor | 0.101 |
| Octanoylcarnitine | ELANE | Protease | 0.101 |
| Octanoylcarnitine | MMP9 | Protease | 0.101 |
| Octanoylcarnitine | CMA1 | Protease | 0.101 |
| Octanoylcarnitine | FABP4 | Fatty acid binding protein family | 0.101 |
| Octanoylcarnitine | CTSK | Protease | 0.101 |
| Octanoylcarnitine | P2RY12 | Family A G protein-coupled receptor | 0.101 |
| Octanoylcarnitine | TRPA1 | Voltage-gated ion channel | 0.101 |
| Octanoylcarnitine | ITGA2B | Membrane receptor | 0.101 |
| Octanoylcarnitine | ITGB3 | Membrane receptor | 0.101 |
| Octanoylcarnitine | FNTA | Enzyme | 0.101 |
| Octanoylcarnitine | FNTB | Enzyme | 0.101 |
| Octanoylcarnitine | PAM | Enzyme | 0.101 |
| Octanoylcarnitine | CFD | Protease | 0.101 |
| Octanoylcarnitine | GYS1 | Enzyme | 0.101 |
| Octanoylcarnitine | AKR1C3 | Enzyme | 0.101 |
| Octanoylcarnitine | FDFT1 | Enzyme | 0.101 |
| Octanoylcarnitine | CAPN1 | Protease | 0.101 |
| Octanoylcarnitine | HTR2B | Family A G protein-coupled receptor | 0.101 |
| Octanoylcarnitine | HRH2 | Family A G protein-coupled receptor | 0.101 |
| Octanoylcarnitine | HTR2A | Family A G protein-coupled receptor | 0.101 |
| Octanoylcarnitine | HTR2C | Family A G protein-coupled receptor | 0.101 |
| Octanoylcarnitine | DRD3 | Family A G protein-coupled receptor | 0.101 |
| Octanoylcarnitine | SIGMAR1 | Membrane receptor | 0.101 |
| Octanoylcarnitine | HTR6 | Family A G protein-coupled receptor | 0.101 |
| Octanoylcarnitine | MMP3 | Protease | 0.101 |
| Octanoylcarnitine | ALK | Kinase | 0.101 |
| Octanoylcarnitine | MMP12 | Protease | 0.101 |
| Octanoylcarnitine | PTGER4 | Family A G protein-coupled receptor | 0.101 |
| Octanoylcarnitine | PGGT1B | Enzyme | 0.101 |
| Octanoylcarnitine | FNTA | Enzyme | 0.101 |
| Octanoylcarnitine | FABP3 | Fatty acid binding protein family | 0.101 |
| Octanoylcarnitine | ABHD6 | Enzyme | 0.101 |
| Octanoylcarnitine | DRD4 | Family A G protein-coupled receptor | 0.101 |
| Octanoylcarnitine | SRC | Kinase | 0.101 |
| Octanoylcarnitine | KDR | Kinase | 0.101 |
| Octanoylcarnitine | IKBKE | Kinase | 0.101 |
| Octanoylcarnitine | FGFR1 | Kinase | 0.101 |
| Octanoylcarnitine | FFAR4 | Family A G protein-coupled receptor | 0.101 |
| Octanoylcarnitine | TBK1 | Kinase | 0.101 |
| Octanoylcarnitine | FFAR2 | Family A G protein-coupled receptor | 0.101 |
| Octanoylcarnitine | CSF1R | Kinase | 0.101 |
| Octanoylcarnitine | EGFR | Kinase | 0.101 |
| Octanoylcarnitine | PIN1 | Enzyme | 0.101 |
| Octanoylcarnitine | PLA2G2A | Enzyme | 0.101 |
| Octanoylcarnitine | AKR1B1 | Enzyme | 0.101 |
| Octanoylcarnitine | CCR1 | Family A G protein-coupled receptor | 0.101 |
| Octanoylcarnitine | TP53 | Transcription factor | 0.101 |
| Phosphoenolpyruvate | PEPD | Protease | 0.999 |
| Carnitine C10:1 | SLC25A20 | Electrochemical transporter | 0.388 |
| Carnitine C10:1 | NR1H4 | Nuclear receptor | 0.214 |
| Carnitine C10:1 | ADAMTS4 | Protease | 0.112 |
| Carnitine C10:1 | TBXA2R | Family A G protein-coupled receptor | 0.112 |
| Carnitine C10:1 | PTGDR2 | Family A G protein-coupled receptor | 0.112 |
| Carnitine C10:1 | P2RY12 | Family A G protein-coupled receptor | 0.112 |
| Carnitine C10:1 | FFAR2 | Family A G protein-coupled receptor | 0.112 |
| Carnitine C10:1 | PTGER1 | Family A G protein-coupled receptor | 0.112 |
| Carnitine C10:1 | PTGER4 | Family A G protein-coupled receptor | 0.112 |
| Carnitine C10:1 | PTGER2 | Family A G protein-coupled receptor | 0.112 |
| Carnitine C10:1 | EDNRA | Family A G protein-coupled receptor | 0.112 |
| Carnitine C10:1 | CTSA | Protease | 0.112 |
| Carnitine C10:1 | CASP1 | Protease | 0.112 |
| Carnitine C10:1 | CYP26A1 | Cytochrome P450 | 0.112 |
| Carnitine C10:1 | TBXAS1 | Cytochrome P450 | 0.112 |
| Carnitine C10:1 | CCR1 | Family A G protein-coupled receptor | 0.112 |
| Carnitine C10:1 | PPARG | Nuclear receptor | 0.112 |
| Carnitine C10:1 | PPARA | Nuclear receptor | 0.112 |
| Carnitine C10:1 | ITGB1 | Membrane receptor | 0.112 |
| Carnitine C10:1 | ITGA4 | Membrane receptor | 0.112 |
| Carnitine C10:1 | MMP12 | Protease | 0.112 |
| Carnitine C10:1 | MMP8 | Protease | 0.112 |
| Carnitine C10:1 | VCP | Primary active transporter | 0.112 |
| Carnitine C10:1 | HIF1A | Transcription factor | 0.112 |
| Carnitine C10:1 | PTGDR | Family A G protein-coupled receptor | 0.112 |
| Carnitine C10:1 | PDE4B | Phosphodiesterase | 0.112 |
| Carnitine C10:1 | AGTR1 | Family A G protein-coupled receptor | 0.112 |
| Carnitine C10:1 | OXER1 | Family A G protein-coupled receptor | 0.112 |
| Carnitine C10:1 | ITGAV | Membrane receptor | 0.112 |
| Carnitine C10:1 | ITGB3 | Membrane receptor | 0.112 |
| Carnitine C10:1 | PDE4A | Phosphodiesterase | 0.112 |
| Carnitine C10:1 | PDE4D | Phosphodiesterase | 0.112 |
| Carnitine C10:1 | PTGIR | Family A G protein-coupled receptor | 0.112 |
| Carnitine C10:1 | ALOX5AP | Other cytosolic protein | 0.112 |
| Carnitine C10:1 | ITGA2B | Membrane receptor | 0.112 |
| Carnitine C10:1 | ITGB3 | Membrane receptor | 0.112 |
| Carnitine C10:1 | MMP13 | Protease | 0.112 |
| Carnitine C10:1 | MMP3 | Protease | 0.112 |
| Carnitine C10:1 | MMP2 | Protease | 0.112 |
| Carnitine C10:1 | PPARD | Nuclear receptor | 0.112 |
| Carnitine C10:1 | MMP10 | Protease | 0.112 |
| Carnitine C10:1 | GZMB | Protease | 0.112 |
| Carnitine C10:1 | MMP9 | Protease | 0.112 |
| Carnitine C10:1 | CPT1A | Enzyme | 0.112 |
| Carnitine C10:1 | RORC | Nuclear receptor | 0.112 |
| Carnitine C10:1 | NTRK1 | Kinase | 0.112 |
| Carnitine C10:1 | FFAR4 | Family A G protein-coupled receptor | 0.112 |
| Carnitine C10:1 | GCGR | Family B G protein-coupled receptor | 0.112 |
| Carnitine C10:1 | PTPRF | Membrane receptor | 0.112 |
| Carnitine C10:1 | PTPN2 | Phosphatase | 0.112 |
| Carnitine C10:1 | ALOX15 | Enzyme | 0.112 |
| Carnitine C10:1 | ALOX12 | Enzyme | 0.112 |
| Carnitine C10:1 | ACE | Protease | 0.112 |
| Carnitine C10:1 | FABP4 | Fatty acid binding protein family | 0.112 |
| Carnitine C10:1 | ITGB5 | Membrane receptor | 0.112 |
| Carnitine C10:1 | ITGAV | Membrane receptor | 0.112 |
| Carnitine C10:1 | CASP3 | Protease | 0.112 |
| Carnitine C10:1 | LTB4R | Family A G protein-coupled receptor | 0.112 |
| Carnitine C10:1 | MDM2 | Other nuclear protein | 0.112 |
| Carnitine C10:1 | NR4A1 | Nuclear receptor | 0.112 |
| Carnitine C10:1 | ESR2 | Nuclear receptor | 0.112 |
| Carnitine C10:1 | ENPP2 | Enzyme | 0.112 |
| Carnitine C10:1 | CMA1 | Protease | 0.112 |
| Carnitine C10:1 | GCG | Unclassified protein | 0.112 |
| Carnitine C10:1 | CXCR2 | Family A G protein-coupled receptor | 0.112 |
| Carnitine C10:1 | CCKBR | Family A G protein-coupled receptor | 0.112 |
| Carnitine C10:1 | FABP3 | Fatty acid binding protein family | 0.112 |
| Carnitine C10:1 | MMP14 | Protease | 0.112 |
| Carnitine C10:1 | FFAR1 | Family A G protein-coupled receptor | 0.112 |
| Carnitine C10:1 | PLA2G2A | Enzyme | 0.112 |
| Carnitine C10:1 | ADAMTS5 | Protease | 0.112 |
| Carnitine C10:1 | MMP1 | Protease | 0.112 |
| Carnitine C10:1 | CNR1 | Family A G protein-coupled receptor | 0.112 |
| Carnitine C10:1 | PLEC | Unclassified protein | 0.112 |
| Carnitine C10:1 | KDM6B | Eraser | 0.112 |
| Carnitine C10:1 | PYGL | Enzyme | 0.112 |
| Carnitine C10:1 | MMEL1 | Hydrolase | 0.112 |
| Carnitine C10:1 | TRPM8 | Voltage-gated ion channel | 0.112 |
| Carnitine C10:1 | ITGAV | Membrane receptor | 0.112 |
| Carnitine C10:1 | ITGB1 | Membrane receptor | 0.112 |
| Carnitine C10:1 | PIK3CB | Enzyme | 0.112 |
| Carnitine C10:1 | FDFT1 | Enzyme | 0.112 |
| Carnitine C10:1 | PIK3CA | Enzyme | 0.112 |
| Carnitine C10:1 | AKR1B1 | Enzyme | 0.112 |
| Carnitine C10:1 | PDGFRB | Kinase | 0.112 |
| Carnitine C10:1 | ADORA1 | Family A G protein-coupled receptor | 0.112 |
| Carnitine C10:1 | PDE3A | Phosphodiesterase | 0.112 |
| Carnitine C10:1 | KDR | Kinase | 0.112 |
| Carnitine C10:1 | PDE3B | Phosphodiesterase | 0.112 |
| Carnitine C10:1 | FGFR1 | Kinase | 0.112 |
| Carnitine C10:1 | F10 | Protease | 0.112 |
| Carnitine C10:1 | ITGA4 | Membrane receptor | 0.112 |
| Carnitine C10:1 | CPT1B | Group translocator | 0.112 |
| Carnitine C10:1 | PTGES | Enzyme | 0.112 |
| Carnitine C10:1 | ADORA2A | Family A G protein-coupled receptor | 0.112 |
| Carnitine C10:1 | NEK2 | Kinase | 0.112 |
| Carnitine C10:1 | GPBAR1 | Family A G protein-coupled receptor | 0.112 |
| Carnitine C10:1 | CPA1 | Protease | 0.112 |
| Carnitine C10:1 | CASP7 | Protease | 0.112 |
| Carnitine C10:1 | ACE2 | Protease | 0.112 |
| Carnitine C10:1 | CASP8 | Protease | 0.112 |
| Carnitine C10:1 | PGGT1B | Enzyme | 0.112 |
| Carnitine C10:1 | FNTA | Enzyme | 0.112 |
| Carnitine C10:1 | ITGB7 | Membrane receptor | 0.112 |
| Carnitine C10:1 | ITGA4 | Membrane receptor | 0.112 |
| Carnitine C10:1 | KDM5C | Eraser | 0.112 |
| Carnitine C10:1 | TGM2 | Enzyme | 0.112 |
| Carnitine C8:0 | SLC25A20 | Electrochemical transporter | 0.551 |
| Carnitine C8:0 | NR1H4 | Nuclear receptor | 0.420 |
| Carnitine C8:0 | CPT1A | Enzyme | 0.101 |
| Carnitine C8:0 | ITGB1 | Membrane receptor | 0.101 |
| Carnitine C8:0 | ITGA4 | Membrane receptor | 0.101 |
| Carnitine C8:0 | MDM2 | Other nuclear protein | 0.101 |
| Carnitine C8:0 | CASP1 | Protease | 0.101 |
| Carnitine C8:0 | BCHE | Hydrolase | 0.101 |
| Carnitine C8:0 | ALOX15 | Enzyme | 0.101 |
| Carnitine C8:0 | ALOX12 | Enzyme | 0.101 |
| Carnitine C8:0 | FFAR1 | Family A G protein-coupled receptor | 0.101 |
| Carnitine C8:0 | PPARG | Nuclear receptor | 0.101 |
| Carnitine C8:0 | PPARD | Nuclear receptor | 0.101 |
| Carnitine C8:0 | PTGDR2 | Family A G protein-coupled receptor | 0.101 |
| Carnitine C8:0 | CPT1B | Group translocator | 0.101 |
| Carnitine C8:0 | ABHD12 | Enzyme | 0.101 |
| Carnitine C8:0 | ABHD16A | Enzyme | 0.101 |
| Carnitine C8:0 | PTGER2 | Family A G protein-coupled receptor | 0.101 |
| Carnitine C8:0 | HSD11B2 | Enzyme | 0.101 |
| Carnitine C8:0 | CCKAR | Family A G protein-coupled receptor | 0.101 |
| Carnitine C8:0 | DUSP3 | Phosphatase | 0.101 |
| Carnitine C8:0 | CTSB | Protease | 0.101 |
| Carnitine C8:0 | VCP | Primary active transporter | 0.101 |
| Carnitine C8:0 | PTGDR | Family A G protein-coupled receptor | 0.101 |
| Carnitine C8:0 | TBXAS1 | Cytochrome P450 | 0.101 |
| Carnitine C8:0 | TBXA2R | Family A G protein-coupled receptor | 0.101 |
| Carnitine C8:0 | CTSH | Protease | 0.101 |
| Carnitine C8:0 | GZMB | Protease | 0.101 |
| Carnitine C8:0 | PDE4B | Phosphodiesterase | 0.101 |
| Carnitine C8:0 | OXER1 | Family A G protein-coupled receptor | 0.101 |
| Carnitine C8:0 | CCKBR | Family A G protein-coupled receptor | 0.101 |
| Carnitine C8:0 | RBP4 | Secreted protein | 0.101 |
| Carnitine C8:0 | MMP2 | Protease | 0.101 |
| Carnitine C8:0 | DAGLA | Enzyme | 0.101 |
| Carnitine C8:0 | MMP1 | Protease | 0.101 |
| Carnitine C8:0 | PLA2G10 | Enzyme | 0.101 |
| Carnitine C8:0 | ITGAV | Membrane receptor | 0.101 |
| Carnitine C8:0 | ITGB3 | Membrane receptor | 0.101 |
| Carnitine C8:0 | CYP26A1 | Cytochrome P450 | 0.101 |
| Carnitine C8:0 | AGTR1 | Family A G protein-coupled receptor | 0.101 |
| Carnitine C8:0 | CTSA | Protease | 0.101 |
| Carnitine C8:0 | SLC22A12 | Electrochemical transporter | 0.101 |
| Carnitine C8:0 | EDNRA | Family A G protein-coupled receptor | 0.101 |
| Carnitine C8:0 | PDE4A | Phosphodiesterase | 0.101 |
| Carnitine C8:0 | PDE4D | Phosphodiesterase | 0.101 |
| Carnitine C8:0 | CMA1 | Protease | 0.101 |
| Carnitine C8:0 | LTB4R | Family A G protein-coupled receptor | 0.101 |
| Carnitine C8:0 | ADORA1 | Family A G protein-coupled receptor | 0.101 |
| Carnitine C8:0 | CTSK | Protease | 0.101 |
| Carnitine C8:0 | PTGER1 | Family A G protein-coupled receptor | 0.101 |
| Carnitine C8:0 | PPARA | Nuclear receptor | 0.101 |
| Carnitine C8:0 | TRPA1 | Voltage-gated ion channel | 0.101 |
| Carnitine C8:0 | CNR1 | Family A G protein-coupled receptor | 0.101 |
| Carnitine C8:0 | ITGA2B | Membrane receptor | 0.101 |
| Carnitine C8:0 | ITGB3 | Membrane receptor | 0.101 |
| Carnitine C8:0 | CFD | Protease | 0.101 |
| Carnitine C8:0 | TOP1 | Isomerase | 0.101 |
| Carnitine C8:0 | ELANE | Protease | 0.101 |
| Carnitine C8:0 | CAPN1 | Protease | 0.101 |
| Carnitine C8:0 | GYS1 | Enzyme | 0.101 |
| Carnitine C8:0 | ALK | Kinase | 0.101 |
| Carnitine C8:0 | PAM | Enzyme | 0.101 |
| Carnitine C8:0 | PTGER4 | Family A G protein-coupled receptor | 0.101 |
| Carnitine C8:0 | FNTA | Enzyme | 0.101 |
| Carnitine C8:0 | FNTB | Enzyme | 0.101 |
| Carnitine C8:0 | PGGT1B | Enzyme | 0.101 |
| Carnitine C8:0 | FNTA | Enzyme | 0.101 |
| Carnitine C8:0 | MMP3 | Protease | 0.101 |
| Carnitine C8:0 | MMP9 | Protease | 0.101 |
| Carnitine C8:0 | MMP12 | Protease | 0.101 |
| Carnitine C8:0 | CHRM4 | Family A G protein-coupled receptor | 0.101 |
| Carnitine C8:0 | HTR2B | Family A G protein-coupled receptor | 0.101 |
| Carnitine C8:0 | HRH2 | Family A G protein-coupled receptor | 0.101 |
| Carnitine C8:0 | CHRM5 | Family A G protein-coupled receptor | 0.101 |
| Carnitine C8:0 | CHRM2 | Family A G protein-coupled receptor | 0.101 |
| Carnitine C8:0 | HTR2A | Family A G protein-coupled receptor | 0.101 |
| Carnitine C8:0 | HTR2C | Family A G protein-coupled receptor | 0.101 |
| Carnitine C8:0 | DRD3 | Family A G protein-coupled receptor | 0.101 |
| Carnitine C8:0 | CHRM3 | Family A G protein-coupled receptor | 0.101 |
| Carnitine C8:0 | SIGMAR1 | Membrane receptor | 0.101 |
| Carnitine C8:0 | HTR6 | Family A G protein-coupled receptor | 0.101 |
| Carnitine C8:0 | FABP4 | Fatty acid binding protein family | 0.101 |
| Carnitine C8:0 | DRD4 | Family A G protein-coupled receptor | 0.101 |
| Carnitine C8:0 | FABP3 | Fatty acid binding protein family | 0.101 |
| Carnitine C8:0 | CSF1R | Kinase | 0.101 |
| Carnitine C8:0 | MMEL1 | Hydrolase | 0.101 |
| Carnitine C8:0 | FFAR4 | Family A G protein-coupled receptor | 0.101 |
| Carnitine C8:0 | FFAR2 | Family A G protein-coupled receptor | 0.101 |
| Carnitine C8:0 | ABHD6 | Enzyme | 0.101 |
| Carnitine C8:0 | EGFR | Kinase | 0.101 |
| Carnitine C8:0 | SRC | Kinase | 0.101 |
| Carnitine C8:0 | NEK2 | Kinase | 0.101 |
| Carnitine C8:0 | P2RY12 | Family A G protein-coupled receptor | 0.101 |
| Carnitine C8:0 | ITGB7 | Membrane receptor | 0.101 |
| Carnitine C8:0 | ITGA4 | Membrane receptor | 0.101 |
| Carnitine C8:0 | PIK3CB | Enzyme | 0.101 |
| Carnitine C8:0 | FDFT1 | Enzyme | 0.101 |
| Carnitine C8:0 | CTSL | Protease | 0.101 |
| Carnitine C8:0 | CCR1 | Family A G protein-coupled receptor | 0.101 |
| Carnitine C8:0 | PDE4C | Phosphodiesterase | 0.101 |
| Carnitine C8:0 | PIK3CA | Enzyme | 0.101 |
| Carnitine C8:0 | TP53 | Transcription factor | 0.101 |
| Carnitine C12:1 | SLC25A20 | Electrochemical transporter | 0.177 |
| Carnitine C12:1 | NR1H4 | Nuclear receptor | 0.137 |
| Carnitine C12:1 | TBXAS1 | Cytochrome P450 | 0.105 |
| Carnitine C12:1 | PTGES | Enzyme | 0.105 |
| Carnitine C12:1 | PTGDR2 | Family A G protein-coupled receptor | 0.105 |
| Carnitine C12:1 | FFAR2 | Family A G protein-coupled receptor | 0.105 |
| Carnitine C12:1 | OXER1 | Family A G protein-coupled receptor | 0.105 |
| Carnitine C12:1 | TRPM8 | Voltage-gated ion channel | 0.105 |
| Carnitine C12:1 | MDM2 | Other nuclear protein | 0.105 |
| Carnitine C12:1 | RXRG | Nuclear receptor | 0.105 |
| Carnitine C12:1 | S1PR1 | Family A G protein-coupled receptor | 0.105 |
| Carnitine C12:1 | GCGR | Family B G protein-coupled receptor | 0.105 |
| Carnitine C12:1 | PTGDR | Family A G protein-coupled receptor | 0.105 |
| Carnitine C12:1 | GCG | Unclassified protein | 0.105 |
| Carnitine C12:1 | PTGER4 | Family A G protein-coupled receptor | 0.105 |
| Carnitine C12:1 | PTGER1 | Family A G protein-coupled receptor | 0.105 |
| Carnitine C12:1 | ITGAV | Membrane receptor | 0.105 |
| Carnitine C12:1 | ITGB3 | Membrane receptor | 0.105 |
| Carnitine C12:1 | LTB4R | Family A G protein-coupled receptor | 0.105 |
| Carnitine C12:1 | MC4R | Family A G protein-coupled receptor | 0.105 |
| Carnitine C12:1 | MC1R | Family A G protein-coupled receptor | 0.105 |
| Carnitine C12:1 | MC5R | Family A G protein-coupled receptor | 0.105 |
| Carnitine C12:1 | RXRA | Nuclear receptor | 0.105 |
| Carnitine C12:1 | PLA2G4A | Enzyme | 0.105 |
| Carnitine C12:1 | P2RY12 | Family A G protein-coupled receptor | 0.105 |
| Carnitine C12:1 | ELANE | Protease | 0.105 |
| Carnitine C12:1 | PTGER3 | Family A G protein-coupled receptor | 0.105 |
| Carnitine C12:1 | PTPN1 | Phosphatase | 0.105 |
| Carnitine C12:1 | ACE | Protease | 0.105 |
| Carnitine C12:1 | KDM2A | Eraser | 0.105 |
| Carnitine C12:1 | KEAP1 | Unclassified protein | 0.105 |
| Carnitine C12:1 | ITGA2B | Membrane receptor | 0.105 |
| Carnitine C12:1 | ITGB3 | Membrane receptor | 0.105 |
| Carnitine C12:1 | KDM5C | Eraser | 0.105 |
| Carnitine C12:1 | CCKBR | Family A G protein-coupled receptor | 0.105 |
| Carnitine C12:1 | FABP4 | Fatty acid binding protein family | 0.105 |
| Carnitine C12:1 | CXCR2 | Family A G protein-coupled receptor | 0.105 |
| Carnitine C12:1 | CNR1 | Family A G protein-coupled receptor | 0.105 |
| Carnitine C12:1 | CNR2 | Family A G protein-coupled receptor | 0.105 |
| Carnitine C12:1 | VCP | Primary active transporter | 0.105 |
| Carnitine C12:1 | ITGB1 | Membrane receptor | 0.105 |
| Carnitine C12:1 | ITGA4 | Membrane receptor | 0.105 |
| Carnitine C12:1 | MMP3 | Protease | 0.105 |
| Carnitine C12:1 | MMP2 | Protease | 0.105 |
| Carnitine C12:1 | ALOX12 | Enzyme | 0.105 |
| Carnitine C12:1 | ALOX5AP | Other cytosolic protein | 0.105 |
| Carnitine C12:1 | ALOX5 | Oxidoreductase | 0.105 |
| Carnitine C12:1 | ITGAL | Adhesion | 0.105 |
| Carnitine C12:1 | RARA | Nuclear receptor | 0.105 |
| Carnitine C12:1 | ITGAL | Membrane receptor | 0.105 |
| Carnitine C12:1 | ICAM1 | Membrane receptor | 0.105 |
| Carnitine C12:1 | ITGB2 | Membrane receptor | 0.105 |
| Carnitine C12:1 | ADORA1 | Family A G protein-coupled receptor | 0.105 |
| Carnitine C12:1 | ADORA2A | Family A G protein-coupled receptor | 0.105 |
| Carnitine C12:1 | ITGB5 | Membrane receptor | 0.105 |
| Carnitine C12:1 | ITGAV | Membrane receptor | 0.105 |
| Carnitine C12:1 | PDE4A | Phosphodiesterase | 0.105 |
| Carnitine C12:1 | PDE4B | Phosphodiesterase | 0.105 |
| Carnitine C12:1 | ALOX15 | Enzyme | 0.105 |
| Carnitine C12:1 | FDFT1 | Enzyme | 0.105 |
| Carnitine C12:1 | AGTR1 | Family A G protein-coupled receptor | 0.105 |
| Carnitine C12:1 | SLC10A2 | Electrochemical transporter | 0.105 |
| Carnitine C12:1 | FABP5 | Fatty acid binding protein family | 0.105 |
| Carnitine C12:1 | CES2 | Enzyme | 0.105 |
| Carnitine C12:1 | RORC | Nuclear receptor | 0.105 |
| Carnitine C12:1 | RARG | Nuclear receptor | 0.105 |
| Carnitine C12:1 | RARB | Nuclear receptor | 0.105 |
| Carnitine C12:1 | ADAM17 | Protease | 0.105 |
| Carnitine C12:1 | PLA2G10 | Enzyme | 0.105 |
| Carnitine C12:1 | MMP13 | Protease | 0.105 |
| Carnitine C12:1 | MMP1 | Protease | 0.105 |
| Carnitine C12:1 | MMP7 | Protease | 0.105 |
| Carnitine C12:1 | FFAR1 | Family A G protein-coupled receptor | 0.105 |
| Carnitine C12:1 | HIF1A | Transcription factor | 0.105 |
| Carnitine C12:1 | PLEC | Unclassified protein | 0.105 |
| Carnitine C12:1 | PRKAG1 | Kinase | 0.105 |
| Carnitine C12:1 | PRKAB1 | Kinase | 0.105 |
| Carnitine C12:1 | PRKAA2 | Kinase | 0.105 |
| Carnitine C12:1 | GYS1 | Enzyme | 0.105 |
| Carnitine C12:1 | CYP26A1 | Cytochrome P450 | 0.105 |
| Carnitine C12:1 | ESR1 | Nuclear receptor | 0.105 |
| Carnitine C12:1 | ESR2 | Nuclear receptor | 0.105 |
| Carnitine C12:1 | PI4KB | Enzyme | 0.105 |
| Carnitine C12:1 | PI4KA | Enzyme | 0.105 |
| Carnitine C12:1 | BRD4 | Reader | 0.105 |
| Carnitine C12:1 | BRD2 | Reader | 0.105 |
| Carnitine C12:1 | BRD3 | Reader | 0.105 |
| Carnitine C12:1 | CCR1 | Family A G protein-coupled receptor | 0.105 |
| Carnitine C12:1 | MMP9 | Protease | 0.105 |
| Carnitine C12:1 | BACE2 | Protease | 0.105 |
| Carnitine C12:1 | CCKAR | Family A G protein-coupled receptor | 0.105 |
| Carnitine C12:1 | PTGFR | Family A G protein-coupled receptor | 0.105 |
| Carnitine C12:1 | NR1H2 | Nuclear receptor | 0.105 |
| Carnitine C12:1 | CASP1 | Protease | 0.105 |
| Carnitine C12:1 | TSPO | Membrane receptor | 0.105 |
| Carnitine C12:1 | ABCC8 | Primary active transporter | 0.105 |
| Carnitine C12:1 | ADORA2B | Family A G protein-coupled receptor | 0.105 |
| Carnitine C12:1 | PIK3CA | Enzyme | 0.105 |
| Carnitine C12:1 | DGAT1 | Enzyme | 0.105 |
| Carnitine C12:1 | CMA1 | Protease | 0.105 |
| Carnitine C12:1 | HPGDS | Transferase | 0.105 |
| Carnitine C12:1 | CTSA | Protease | 0.105 |
| Carnitine C12:1 | ITGA4 | Membrane receptor | 0.105 |
| Carnitine C12:1 | MAPK14 | Kinase | 0.105 |
| Carnitine C12:1 | TGFBR1 | Kinase | 0.105 |
| Carnitine C12:1 | EPHX1 | Protease | 0.105 |
| Carnitine C12:1 | PYGL | Enzyme | 0.105 |
| Carnitine C12:1 | GPBAR1 | Family A G protein-coupled receptor | 0.105 |
| 3-Dehydrosphinganine | CACNA2D1 | Calcium channel auxiliary subunit alpha2delta family | 0.139 |
| 3-Dehydrosphinganine | GRM5 | Family C G protein-coupled receptor | 0.114 |
| 3-Dehydrosphinganine | SHBG | Secreted protein | 0.106 |
| 3-Dehydrosphinganine | LTA4H | Protease | 0.106 |
| 3'-Aenylic Acid | SRC | Kinase | 0.651 |
| 3'-Aenylic Acid | FBP1 | Enzyme | 0.651 |
| 3'-Aenylic Acid | HPRT1 | Enzyme | 0.127 |
| 3'-Aenylic Acid | FHIT | Enzyme | 0.110 |
| 3'-Aenylic Acid | QARS | Enzyme | 0.110 |
| 3'-Aenylic Acid | DNPH1 | Hydrolase | 0.110 |
| 3'-Aenylic Acid | RARS | Enzyme | 0.110 |
| 3'-Aenylic Acid | MAPKAPK2 | Kinase | 0.110 |
| 3'-Aenylic Acid | HSPA1A | Other cytosolic protein | 0.110 |
| 3'-Aenylic Acid | P2RY1 | Family A G protein-coupled receptor | 0.110 |
| 3'-Aenylic Acid | P2RY11 | Family A G protein-coupled receptor | 0.110 |
| 3'-Aenylic Acid | P2RX1 | Ligand-gated ion channel | 0.102 |
| 3'-Aenylic Acid | AKT1 | Kinase | 0.102 |
| 3'-Aenylic Acid | ADORA2A | Family A G protein-coupled receptor | 0.102 |
| 3'-Aenylic Acid | ADORA3 | Family A G protein-coupled receptor | 0.102 |
| 3'-Aenylic Acid | DPP4 | Protease | 0.102 |
| 3'-Aenylic Acid | ADK | Enzyme | 0.102 |
| 3'-Aenylic Acid | ADCY10 | Enzyme | 0.102 |
| 3'-Aenylic Acid | HSPA8 | Other cytosolic protein | 0.102 |
| 3'-Aenylic Acid | HSPA5 | Unclassified protein | 0.102 |
| 3'-Aenylic Acid | RNASE2 | Enzyme | 0.102 |
| 3'-Aenylic Acid | RNASE1 | Enzyme | 0.102 |
| 3'-Aenylic Acid | RNASEL | Hydrolase | 0.102 |
| 3'-Aenylic Acid | PARG | Enzyme | 0.102 |
| 3'-Aenylic Acid | P2RY12 | Family A G protein-coupled receptor | 0.102 |
| 3'-Aenylic Acid | IMPDH2 | Oxidoreductase | 0.102 |
| 3'-Aenylic Acid | AHCY | Enzyme | 0.102 |
| 3'-Aenylic Acid | EHMT1 | Writer | 0.102 |
| 3'-Aenylic Acid | EHMT2 | Writer | 0.102 |
| 3'-Aenylic Acid | ENPP1 | Enzyme | 0.102 |
| 3'-Aenylic Acid | GAPDH | Oxidoreductase | 0.102 |
| 3'-Aenylic Acid | ADA | Hydrolase | 0.102 |
| 3'-Aenylic Acid | EIF4E | Other nuclear protein | 0.102 |
| 3'-Aenylic Acid | AMD1 | Enzyme | 0.102 |
| 3'-Aenylic Acid | SETD7 | Writer | 0.102 |
| 3'-Aenylic Acid | TYMS | Transferase | 0.102 |
| 3'-Aenylic Acid | ASNS | Enzyme | 0.102 |
| 3'-Aenylic Acid | DTYMK | Transferase | 0.102 |
| 3'-Aenylic Acid | CHAT | Enzyme | 0.102 |
| 3'-Aenylic Acid | SRM | Enzyme | 0.102 |
| 3'-Aenylic Acid | ADORA2B | Family A G protein-coupled receptor | 0.102 |
| 3'-Aenylic Acid | HLCS | Enzyme | 0.102 |
| 3'-Aenylic Acid | CD38 | Enzyme | 0.102 |
| 3'-Aenylic Acid | SLC29A1 | Electrochemical transporter | 0.102 |
| 3'-Aenylic Acid | ADORA1 | Family A G protein-coupled receptor | 0.102 |
| 3'-Aenylic Acid | MCL1 | Other cytosolic protein | 0.102 |
| 3'-Aenylic Acid | MARS | Enzyme | 0.102 |
| 3'-Aenylic Acid | PRMT1 | Writer | 0.102 |
| 3'-Aenylic Acid | EGFR | Kinase | 0.102 |
| 3'-Aenylic Acid | FDPS | Transferase | 0.102 |
| 3'-Aenylic Acid | PDCD4 | Unclassified protein | 0.102 |
| 3'-Aenylic Acid | IMPDH1 | Oxidoreductase | 0.102 |
| 3'-Aenylic Acid | SETD2 | Writer | 0.102 |
| 3'-Aenylic Acid | CARM1 | Writer | 0.102 |
| 3'-Aenylic Acid | CA12 | Lyase | 0.102 |
| 3'-Aenylic Acid | CA14 | Lyase | 0.102 |
| 3'-Aenylic Acid | CA9 | Lyase | 0.102 |
| 3'-Aenylic Acid | MTAP | Enzyme | 0.102 |
| 3'-Aenylic Acid | TRPM2 | Voltage-gated ion channel | 0.102 |
| 3'-Aenylic Acid | EP300 | Writer | 0.102 |
| Carnitine C11:1 | SLC25A20 | Electrochemical transporter | 0.768 |
| Carnitine C11:1 | NR1H4 | Nuclear receptor | 0.368 |
| Carnitine C11:1 | CPT1A | Enzyme | 0.102 |
| Carnitine C11:1 | CPT1B | Group translocator | 0.102 |
| Carnitine C11:1 | P2RY12 | Family A G protein-coupled receptor | 0.102 |
| Carnitine C11:1 | TBXA2R | Family A G protein-coupled receptor | 0.102 |
| Carnitine C11:1 | PTGDR2 | Family A G protein-coupled receptor | 0.102 |
| Carnitine C11:1 | PPARG | Nuclear receptor | 0.102 |
| Carnitine C11:1 | NR4A1 | Nuclear receptor | 0.102 |
| Carnitine C11:1 | PPARA | Nuclear receptor | 0.102 |
| Carnitine C11:1 | ESR2 | Nuclear receptor | 0.102 |
| Carnitine C11:1 | MMP1 | Protease | 0.102 |
| Carnitine C11:1 | MMP2 | Protease | 0.102 |
| Carnitine C11:1 | PTGER4 | Family A G protein-coupled receptor | 0.102 |
| Carnitine C11:1 | PTGER2 | Family A G protein-coupled receptor | 0.102 |
| Carnitine C11:1 | PTGDR | Family A G protein-coupled receptor | 0.102 |
| Carnitine C11:1 | ALOX12 | Enzyme | 0.102 |
| Carnitine C11:1 | PTGER1 | Family A G protein-coupled receptor | 0.102 |
| Carnitine C11:1 | PLA2G2A | Enzyme | 0.102 |
| Carnitine C11:1 | PLA2G10 | Enzyme | 0.102 |
| Carnitine C11:1 | BCHE | Hydrolase | 0.102 |
| Carnitine C11:1 | ALOX5AP | Other cytosolic protein | 0.102 |
| Carnitine C11:1 | CYP26A1 | Cytochrome P450 | 0.102 |
| Carnitine C11:1 | ITGAV | Membrane receptor | 0.102 |
| Carnitine C11:1 | ITGB3 | Membrane receptor | 0.102 |
| Carnitine C11:1 | ITGA2B | Membrane receptor | 0.102 |
| Carnitine C11:1 | ITGB3 | Membrane receptor | 0.102 |
| Carnitine C11:1 | ITGB5 | Membrane receptor | 0.102 |
| Carnitine C11:1 | ITGAV | Membrane receptor | 0.102 |
| Carnitine C11:1 | TBXAS1 | Cytochrome P450 | 0.102 |
| Carnitine C11:1 | FFAR1 | Family A G protein-coupled receptor | 0.102 |
| Carnitine C11:1 | PPARD | Nuclear receptor | 0.102 |
| Carnitine C11:1 | APLNR | Family A G protein-coupled receptor | 0.102 |
| Carnitine C11:1 | ABHD12 | Enzyme | 0.102 |
| Carnitine C11:1 | ABHD16A | Enzyme | 0.102 |
| Carnitine C11:1 | VCP | Primary active transporter | 0.102 |
| Carnitine C11:1 | GCGR | Family B G protein-coupled receptor | 0.102 |
| Carnitine C11:1 | GIPR | Family B G protein-coupled receptor | 0.102 |
| Carnitine C11:1 | DAGLA | Enzyme | 0.102 |
| Carnitine C11:1 | ADAMTS5 | Protease | 0.102 |
| Carnitine C11:1 | ADAMTS4 | Protease | 0.102 |
| Carnitine C11:1 | CASP3 | Protease | 0.102 |
| Carnitine C11:1 | CXCR2 | Family A G protein-coupled receptor | 0.102 |
| Carnitine C11:1 | MMP13 | Protease | 0.102 |
| Carnitine C11:1 | MMP3 | Protease | 0.102 |
| Carnitine C11:1 | CASP7 | Protease | 0.102 |
| Carnitine C11:1 | CASP8 | Protease | 0.102 |
| Carnitine C11:1 | MMP12 | Protease | 0.102 |
| Carnitine C11:1 | MMP8 | Protease | 0.102 |
| Carnitine C11:1 | CASP1 | Protease | 0.102 |
| Carnitine C11:1 | ITGA4 | Membrane receptor | 0.102 |
| Carnitine C11:1 | PLA2G4B | Enzyme | 0.102 |
| Carnitine C11:1 | PDE10A | Phosphodiesterase | 0.102 |
| Carnitine C11:1 | CTSA | Protease | 0.102 |
| Carnitine C11:1 | CTSH | Protease | 0.102 |
| Carnitine C11:1 | ITGB1 | Membrane receptor | 0.102 |
| Carnitine C11:1 | ITGA4 | Membrane receptor | 0.102 |
| Carnitine C11:1 | PDGFRB | Kinase | 0.102 |
| Carnitine C11:1 | FNTA | Enzyme | 0.102 |
| Carnitine C11:1 | FNTB | Enzyme | 0.102 |
| Carnitine C11:1 | SRC | Kinase | 0.102 |
| Carnitine C11:1 | PDE4B | Phosphodiesterase | 0.102 |
| Carnitine C11:1 | KDR | Kinase | 0.102 |
| Carnitine C11:1 | PDE4D | Phosphodiesterase | 0.102 |
| Carnitine C11:1 | FGFR1 | Kinase | 0.102 |
| Carnitine C11:1 | PLA2G4A | Enzyme | 0.102 |
| Carnitine C11:1 | ITGB7 | Membrane receptor | 0.102 |
| Carnitine C11:1 | ITGA4 | Membrane receptor | 0.102 |
| Carnitine C11:1 | TGM2 | Enzyme | 0.102 |
| Carnitine C11:1 | CCKBR | Family A G protein-coupled receptor | 0.102 |
| Carnitine C11:1 | MMP9 | Protease | 0.102 |
| Carnitine C11:1 | PYGL | Enzyme | 0.102 |
| Carnitine C11:1 | CNR1 | Family A G protein-coupled receptor | 0.102 |
| Carnitine C11:1 | TRPM8 | Voltage-gated ion channel | 0.102 |
| Carnitine C11:1 | GCG | Unclassified protein | 0.102 |
| Carnitine C11:1 | ITGAL | Adhesion | 0.102 |
| Carnitine C11:1 | MMP7 | Protease | 0.102 |
| Carnitine C11:1 | MMP10 | Protease | 0.102 |
| Carnitine C11:1 | PGGT1B | Enzyme | 0.102 |
| Carnitine C11:1 | FNTA | Enzyme | 0.102 |
| Carnitine C11:1 | PIN1 | Enzyme | 0.102 |
| Carnitine C11:1 | PDE4A | Phosphodiesterase | 0.102 |
| Carnitine C11:1 | MDM2 | Other nuclear protein | 0.102 |
| Carnitine C11:1 | PTGIR | Family A G protein-coupled receptor | 0.102 |
| Carnitine C11:1 | CDC25C | Phosphatase | 0.102 |
| Carnitine C11:1 | LTB4R | Family A G protein-coupled receptor | 0.102 |
| Carnitine C11:1 | CDC25B | Phosphatase | 0.102 |
| Carnitine C11:1 | WEE1 | Kinase | 0.102 |
| Carnitine C11:1 | MMEL1 | Hydrolase | 0.102 |
| Carnitine C11:1 | CCKAR | Family A G protein-coupled receptor | 0.102 |
| Carnitine C11:1 | MMP14 | Protease | 0.102 |
| Carnitine C11:1 | DGAT1 | Enzyme | 0.102 |
| Carnitine C11:1 | CTSB | Protease | 0.102 |
| Carnitine C11:1 | S1PR4 | Family A G protein-coupled receptor | 0.102 |
| Carnitine C11:1 | STAT3 | Transcription factor | 0.102 |
| Carnitine C11:1 | SYK | Kinase | 0.102 |
| Carnitine C11:1 | ABCC1 | Primary active transporter | 0.102 |
| Carnitine C11:1 | AVPR2 | Family A G protein-coupled receptor | 0.102 |
| Carnitine C11:1 | OXTR | Family A G protein-coupled receptor | 0.102 |
| Carnitine C11:1 | SIRT1 | Eraser | 0.102 |
| Carnitine C11:1 | ACP1 | Phosphatase | 0.102 |
| Carnitine C11:1 | CYSLTR1 | Family A G protein-coupled receptor | 0.102 |
| Carnitine C11:1 | AURKB | Kinase | 0.102 |
| Carnitine C11:1 | PTPN11 | Phosphatase | 0.102 |
| Carnitine C11:1 | AURKA | Kinase | 0.102 |
| Carnitine C11:1 | F10 | Protease | 0.102 |
| Carnitine C11:1 | SLC10A2 | Electrochemical transporter | 0.102 |
| Adenosine-5-Monophosphate | SRC | Kinase | 0.986 |
| Adenosine-5-Monophosphate | FBP1 | Enzyme | 0.986 |
| Adenosine-5-Monophosphate | HPRT1 | Enzyme | 0.177 |
| Adenosine-5-Monophosphate | FHIT | Enzyme | 0.135 |
| Adenosine-5-Monophosphate | QARS | Enzyme | 0.135 |
| Adenosine-5-Monophosphate | P2RY1 | Family A G protein-coupled receptor | 0.127 |
| Adenosine-5-Monophosphate | P2RY11 | Family A G protein-coupled receptor | 0.127 |
| Adenosine-5-Monophosphate | MAPKAPK2 | Kinase | 0.127 |
| Adenosine-5-Monophosphate | HSPA1A | Other cytosolic protein | 0.127 |
| Adenosine-5-Monophosphate | RARS | Enzyme | 0.118 |
| Adenosine-5-Monophosphate | DNPH1 | Hydrolase | 0.118 |
| Adenosine-5-Monophosphate | HSPA8 | Other cytosolic protein | 0.118 |
| Adenosine-5-Monophosphate | HSPA5 | Unclassified protein | 0.118 |
| Adenosine-5-Monophosphate | AKT1 | Kinase | 0.110 |
| Adenosine-5-Monophosphate | PARG | Enzyme | 0.110 |
| Adenosine-5-Monophosphate | RNASEL | Hydrolase | 0.102 |
| Adenosine-5-Monophosphate | ADORA2A | Family A G protein-coupled receptor | 0.102 |
| Adenosine-5-Monophosphate | ADORA3 | Family A G protein-coupled receptor | 0.102 |
| Adenosine-5-Monophosphate | DPP4 | Protease | 0.102 |
| Adenosine-5-Monophosphate | ADK | Enzyme | 0.102 |
| Adenosine-5-Monophosphate | EIF4E | Other nuclear protein | 0.102 |
| Adenosine-5-Monophosphate | P2RX1 | Ligand-gated ion channel | 0.102 |
| Adenosine-5-Monophosphate | IMPDH2 | Oxidoreductase | 0.102 |
| Adenosine-5-Monophosphate | RNASE2 | Enzyme | 0.102 |
| Adenosine-5-Monophosphate | RNASE1 | Enzyme | 0.102 |
| Adenosine-5-Monophosphate | P2RY12 | Family A G protein-coupled receptor | 0.102 |
| Adenosine-5-Monophosphate | TYMS | Transferase | 0.102 |
| Adenosine-5-Monophosphate | ENPP1 | Enzyme | 0.102 |
| Adenosine-5-Monophosphate | ADCY10 | Enzyme | 0.102 |
| Adenosine-5-Monophosphate | ASNS | Enzyme | 0.102 |
| Adenosine-5-Monophosphate | AHCY | Enzyme | 0.102 |
| Adenosine-5-Monophosphate | EHMT1 | Writer | 0.102 |
| Adenosine-5-Monophosphate | EHMT2 | Writer | 0.102 |
| Adenosine-5-Monophosphate | DTYMK | Transferase | 0.102 |
| Adenosine-5-Monophosphate | GAPDH | Oxidoreductase | 0.102 |
| Adenosine-5-Monophosphate | ADA | Hydrolase | 0.102 |
| Adenosine-5-Monophosphate | AMD1 | Enzyme | 0.102 |
| Adenosine-5-Monophosphate | HLCS | Enzyme | 0.102 |
| Adenosine-5-Monophosphate | CD38 | Enzyme | 0.102 |
| Adenosine-5-Monophosphate | SETD7 | Writer | 0.102 |
| Adenosine-5-Monophosphate | CHAT | Enzyme | 0.102 |
| Adenosine-5-Monophosphate | CDC42 | Unclassified protein | 0.102 |
| Adenosine-5-Monophosphate | RAC1 | Unclassified protein | 0.102 |
| Adenosine-5-Monophosphate | SRM | Enzyme | 0.102 |
| Adenosine-5-Monophosphate | ADORA2B | Family A G protein-coupled receptor | 0.102 |
| Adenosine-5-Monophosphate | ATIC | Enzyme | 0.102 |
| Adenosine-5-Monophosphate | IMPDH1 | Oxidoreductase | 0.102 |
| Adenosine-5-Monophosphate | SLC29A1 | Electrochemical transporter | 0.102 |
| Adenosine-5-Monophosphate | TRPM2 | Voltage-gated ion channel | 0.102 |
| Adenosine-5-Monophosphate | MARS | Enzyme | 0.102 |
| Adenosine-5-Monophosphate | ADORA1 | Family A G protein-coupled receptor | 0.102 |
| Adenosine-5-Monophosphate | ST6GAL1 | Transferase | 0.102 |
| Adenosine-5-Monophosphate | SETD2 | Writer | 0.102 |
| Adenosine-5-Monophosphate | CARM1 | Writer | 0.102 |
| Adenosine-5-Monophosphate | PRMT1 | Writer | 0.102 |
| Adenosine-5-Monophosphate | BCHE | Hydrolase | 0.102 |
| Adenosine-5-Monophosphate | MCL1 | Other cytosolic protein | 0.102 |
| Adenosine-5-Monophosphate | EGFR | Kinase | 0.102 |
| Adenosine-5-Monophosphate | FDPS | Transferase | 0.102 |
| Adenosine-5-Monophosphate | ADH1A | Oxidoreductase | 0.102 |
| Adenosine-5-Monophosphate | PDCD4 | Unclassified protein | 0.102 |
| Adenosine-5-Monophosphate | MTAP | Enzyme | 0.102 |
| Adenosine-5-Monophosphate | EP300 | Writer | 0.102 |
| Adenosine-5-Monophosphate | LDHB | Enzyme | 0.102 |
| 9,10,13-TriHOME | PTGER1 | Family A G protein-coupled receptor | 0.210 |
| 9,10,13-TriHOME | PTGER4 | Family A G protein-coupled receptor | 0.210 |
| 9,10,13-TriHOME | PTGER2 | Family A G protein-coupled receptor | 0.210 |
| 9,10,13-TriHOME | PTGIR | Family A G protein-coupled receptor | 0.202 |
| 9,10,13-TriHOME | PPARG | Nuclear receptor | 0.160 |
| 9,10,13-TriHOME | PTGER3 | Family A G protein-coupled receptor | 0.160 |
| 9,10,13-TriHOME | PTGFR | Family A G protein-coupled receptor | 0.160 |
| 9,10,13-TriHOME | SLC22A6 | Electrochemical transporter | 0.152 |
| 9,10,13-TriHOME | PPARA | Nuclear receptor | 0.152 |
| 9,10,13-TriHOME | PPARD | Nuclear receptor | 0.152 |
| 9,10,13-TriHOME | PTGDR | Family A G protein-coupled receptor | 0.135 |
| 9,10,13-TriHOME | SLC6A4 | Electrochemical transporter | 0.110 |
| 9,10,13-TriHOME | LTB4R | Family A G protein-coupled receptor | 0.102 |
| 9,10,13-TriHOME | PTPN1 | Phosphatase | 0.102 |
| 9,10,13-TriHOME | FNTA | Enzyme | 0.102 |
| 9,10,13-TriHOME | FNTB | Enzyme | 0.102 |
| 9,10,13-TriHOME | TERT | Enzyme | 0.102 |
| 9,10,13-TriHOME | FABP5 | Fatty acid binding protein family | 0.102 |
| 9,10,13-TriHOME | FABP1 | Fatty acid binding protein family | 0.102 |
| 9,10,13-TriHOME | SERPINA6 | Secreted protein | 0.102 |
| 9,10,13-TriHOME | SHBG | Secreted protein | 0.102 |
| 9,10,13-TriHOME | G6PD | Enzyme | 0.102 |
| 9,10,13-TriHOME | PTPN6 | Phosphatase | 0.102 |
| 9,10,13-TriHOME | CYP19A1 | Cytochrome P450 | 0.102 |
| 9,10,13-TriHOME | FAAH | Enzyme | 0.102 |
| 9,10,13-TriHOME | RORA | Nuclear receptor | 0.102 |
| 9,10,13-TriHOME | HMGCR | Oxidoreductase | 0.102 |
| 9,10,13-TriHOME | GSK3A | Kinase | 0.102 |
| 9,10,13-TriHOME | AURKA | Kinase | 0.102 |
| 9,10,13-TriHOME | MAPK14 | Kinase | 0.102 |
| 9,10,13-TriHOME | MAPK10 | Kinase | 0.102 |
| 9,10,13-TriHOME | PDE5A | Phosphodiesterase | 0.102 |
| 9,10,13-TriHOME | TBXA2R | Family A G protein-coupled receptor | 0.102 |
| 9,10,13-TriHOME | GRM2 | Family C G protein-coupled receptor | 0.102 |
| 9,10,13-TriHOME | ESR2 | Nuclear receptor | 0.102 |
| 9,10,13-TriHOME | PTGS1 | Oxidoreductase | 0.102 |
| 9,10,13-TriHOME | CES2 | Enzyme | 0.102 |
| 9,10,13-TriHOME | LCK | Kinase | 0.102 |
| 9,10,13-TriHOME | THRA | Nuclear receptor | 0.102 |
| 9,10,13-TriHOME | THRB | Nuclear receptor | 0.102 |
| 9,10,13-TriHOME | MMP3 | Protease | 0.102 |
| 9,10,13-TriHOME | MMP8 | Protease | 0.102 |
| 9,10,13-TriHOME | TNF | Secreted protein | 0.102 |
| 9,10,13-TriHOME | PTPN2 | Phosphatase | 0.102 |
| 9,10,13-TriHOME | ITGA2B | Membrane receptor | 0.102 |
| 9,10,13-TriHOME | ITGB3 | Membrane receptor | 0.102 |
| 9,10,13-TriHOME | NR3C1 | Nuclear receptor | 0.102 |
| 9,10,13-TriHOME | PTPN22 | Phosphatase | 0.102 |
| 9,10,13-TriHOME | CYP17A1 | Cytochrome P450 | 0.102 |
| 9,10,13-TriHOME | DPP4 | Protease | 0.102 |
| 9,10,13-TriHOME | NR1H3 | Nuclear receptor | 0.102 |
| 9,10,13-TriHOME | AMPD1 | Enzyme | 0.102 |
| 9,10,13-TriHOME | TYRO3 | Kinase | 0.102 |
| 9,10,13-TriHOME | PIM2 | Kinase | 0.102 |
| 9,10,13-TriHOME | PIK3CA | Enzyme | 0.102 |
| 9,10,13-TriHOME | IMPDH1 | Oxidoreductase | 0.102 |
| 9,10,13-TriHOME | IMPDH2 | Oxidoreductase | 0.102 |
| 9,10,13-TriHOME | CASP3 | Protease | 0.102 |
| 9,10,13-TriHOME | FFAR1 | Family A G protein-coupled receptor | 0.102 |
| 9,10,13-TriHOME | ITGAV | Membrane receptor | 0.102 |
| 9,10,13-TriHOME | ITGB3 | Membrane receptor | 0.102 |
| 9,10,13-TriHOME | ITGB5 | Membrane receptor | 0.102 |
| 9,10,13-TriHOME | ITGAV | Membrane receptor | 0.102 |
| 9,10,13-TriHOME | ITGAV | Membrane receptor | 0.102 |
| 9,10,13-TriHOME | ITGB6 | Membrane receptor | 0.102 |
| 9,10,13-TriHOME | SRD5A2 | Oxidoreductase | 0.102 |
| 9,10,13-TriHOME | GCGR | Family B G protein-coupled receptor | 0.102 |
| 9,10,13-TriHOME | GIPR | Family B G protein-coupled receptor | 0.102 |
| 9,10,13-TriHOME | ITGAV | Membrane receptor | 0.102 |
| 9,10,13-TriHOME | ITGB1 | Membrane receptor | 0.102 |
| 9,10,13-TriHOME | TYMS | Transferase | 0.102 |
| 9,10,13-TriHOME | PTPN7 | Phosphatase | 0.102 |
| 9,10,13-TriHOME | PTPRC | Enzyme | 0.102 |
| 9,10,13-TriHOME | PTGDR2 | Family A G protein-coupled receptor | 0.102 |
| 9,10,13-TriHOME | PRKCA | Kinase | 0.102 |
| 9,10,13-TriHOME | ITGAV | Membrane receptor | 0.102 |
| 9,10,13-TriHOME | TGFBR1 | Kinase | 0.102 |
| 9,10,13-TriHOME | PDE6D | Phosphodiesterase | 0.102 |
| 9,10,13-TriHOME | MMP1 | Protease | 0.102 |
| 9,10,13-TriHOME | EDNRB | Family A G protein-coupled receptor | 0.102 |
| 9,10,13-TriHOME | EDNRA | Family A G protein-coupled receptor | 0.102 |
| 9,10,13-TriHOME | PLK1 | Kinase | 0.102 |
| 9,10,13-TriHOME | GRB2 | Other cytosolic protein | 0.102 |
| 9,10,13-TriHOME | CDK6 | Kinase | 0.102 |
| 9,10,13-TriHOME | CDK4 | Kinase | 0.102 |
| 9,10,13-TriHOME | ESR1 | Nuclear receptor | 0.102 |
| 9,10,13-TriHOME | PDE4D | Phosphodiesterase | 0.102 |
| 9,10,13-TriHOME | JAK1 | Kinase | 0.102 |
| 9,10,13-TriHOME | JAK2 | Kinase | 0.102 |
| 9,10,13-TriHOME | MMP9 | Protease | 0.102 |
| 9,10,13-TriHOME | CSNK2A1 | Kinase | 0.102 |
| 9,10,13-TriHOME | DPP7 | Protease | 0.102 |
| 9,10,13-TriHOME | SCD | Enzyme | 0.102 |
| 9,10,13-TriHOME | CNR1 | Family A G protein-coupled receptor | 0.102 |
| 9,10,13-TriHOME | HSD11B1 | Enzyme | 0.102 |
| 9,10,13-TriHOME | CASR | Family C G protein-coupled receptor | 0.102 |

**Table S9. Top50 related genes with arterial stiffness retreived from NCBI database**

| **GeneID** | **Symbol** | **Description** |
| --- | --- | --- |
| 19 | ABCA1 | ATP binding cassette subfamily A member 1 |
| 81 | ACTN4 | actinin alpha 4 |
| 133 | ADM | adrenomedullin |
| 148 | ADRA1A | adrenoceptor alpha 1A |
| 151 | ADRA2B | adrenoceptor alpha 2B |
| 177 | AGER | advanced glycosylation end-product specific receptor |
| 183 | AGT | angiotensinogen |
| 185 | AGTR1 | angiotensin II receptor type 1 |
| 197 | AHSG | alpha 2-HS glycoprotein |
| 207 | AKT1 | AKT serine/threonine kinase 1 |
| 213 | ALB | albumin |
| 249 | ALPL | alkaline phosphatase, biomineralization associated |
| 276 | AMY1A | amylase alpha 1A |
| 285 | ANGPT2 | angiopoietin 2 |
| 335 | APOA1 | apolipoprotein A1 |
| 338 | APOB | apolipoprotein B |
| 351 | APP | amyloid beta precursor protein |
| 551 | AVP | arginine vasopressin |
| 633 | BGN | biglycan |
| 650 | BMP2 | bone morphogenetic protein 2 |
| 652 | BMP4 | bone morphogenetic protein 4 |
| 673 | BRAF | B-Raf proto-oncogene, serine/threonine kinase |
| 814 | CAMK4 | calcium/calmodulin dependent protein kinase IV |
| 857 | CAV1 | caveolin 1 |
| 860 | RUNX2 | RUNX family transcription factor 2 |
| 929 | CD14 | CD14 molecule |
| 960 | CD44 | CD44 molecule (IN blood group) |
| 1003 | CDH5 | cadherin 5 |
| 1012 | CDH13 | cadherin 13 |
| 1113 | CHGA | chromogranin A |
| 1116 | CHI3L1 | chitinase 3 like 1 |
| 1282 | COL4A1 | collagen type IV alpha 1 chain |
| 1289 | COL5A1 | collagen type V alpha 1 chain |
| 1401 | CRP | C-reactive protein |
| 1471 | CST3 | cystatin C |
| 1489 | CTF1 | cardiotrophin 1 |
| 1499 | CTNNB1 | catenin beta 1 |
| 1535 | CYBA | cytochrome b-245 alpha chain |
| 1585 | CYP11B2 | cytochrome P450 family 11 subfamily B member 2 |
| 1586 | CYP17A1 | cytochrome P450 family 17 subfamily A member 1 |
| 1588 | CYP19A1 | cytochrome P450 family 19 subfamily A member 1 |
| 1636 | ACE | angiotensin I converting enzyme |
| 1906 | EDN1 | endothelin 1 |
| 1956 | EGFR | epidermal growth factor receptor |
| 2006 | ELN | elastin |
| 2099 | ESR1 | estrogen receptor 1 |
| 2100 | ESR2 | estrogen receptor 2 |
| 2147 | F2 | coagulation factor II, thrombin |
| 2162 | F13A1 | coagulation factor XIII A chain |
| 2167 | FABP4 | fatty acid binding protein 4 |

**Table S10. Top50 related genes with PWV retreived from GeneCards database**

| **Gene Symbol** | **Description** | **Category** | **Uniprot ID** | **Relevance score** |
| --- | --- | --- | --- | --- |
| ADIPOQ | Adiponectin, C1Q And Collagen Domain Containing | Protein Coding | Q15848 | 7.346888065 |
| AGTR1 | Angiotensin II Receptor Type 1 | Protein Coding | P30556 | 6.761303425 |
| CRP | C-Reactive Protein | Protein Coding | P02741 | 6.519898415 |
| ACE | Angiotensin I Converting Enzyme | Protein Coding | P12821 | 5.68510437 |
| IL6 | Interleukin 6 | Protein Coding | P05231 | 5.613385201 |
| NOS3 | Nitric Oxide Synthase 3 | Protein Coding | P29474 | 5.323474884 |
| MMP9 | Matrix Metallopeptidase 9 | Protein Coding | P14780 | 4.584153175 |
| PPARG | Peroxisome Proliferator Activated Receptor Gamma | Protein Coding | P37231 | 4.560593128 |
| TNFRSF11B | TNF Receptor Superfamily Member 11b | Protein Coding | O00300 | 4.51330328 |
| SOST | Sclerostin | Protein Coding | Q9BQB4 | 4.10150528 |
| TNF | Tumor Necrosis Factor | Protein Coding | P01375 | 4.044583797 |
| AGT | Angiotensinogen | Protein Coding | P01019 | 4.015054703 |
| ESR1 | Estrogen Receptor 1 | Protein Coding | P03372 | 3.984737158 |
| GGT1 | Gamma-Glutamyltransferase 1 | Protein Coding | P19440 | 3.953565598 |
| ADM | Adrenomedullin | Protein Coding | P35318 | 3.874518394 |
| GPR25 | G Protein-Coupled Receptor 25 | Protein Coding | O00155 | 3.870854378 |
| LEP | Leptin | Protein Coding | P41159 | 3.825338602 |
| GPC6 | Glypican 6 | Protein Coding | Q9Y625 | 3.765616894 |
| APOB | Apolipoprotein B | Protein Coding | P04114 | 3.733947992 |
| AGER | Advanced Glycosylation End-Product Specific Receptor | Protein Coding | Q15109 | 3.730064154 |
| ALB | Albumin | Protein Coding | P02768 | 3.705199242 |
| CCL2 | C-C Motif Chemokine Ligand 2 | Protein Coding | P13500 | 3.702776432 |
| GNB3 | G Protein Subunit Beta 3 | Protein Coding | P16520 | 3.603304386 |
| FABP4 | Fatty Acid Binding Protein 4 | Protein Coding | P15090 | 3.516394138 |
| INS | Insulin | Protein Coding | P01308 | 3.456547499 |
| MGP | Matrix Gla Protein | Protein Coding | P08493 | 3.456547499 |
| COL4A1 | Collagen Type IV Alpha 1 Chain | Protein Coding | P02462 | 3.383816481 |
| FBN1 | Fibrillin 1 | Protein Coding | P35555 | 3.325903893 |
| NPPA | Natriuretic Peptide A | Protein Coding | P01160 | 3.325903893 |
| TIMP1 | TIMP Metallopeptidase Inhibitor 1 | Protein Coding | P01033 | 3.266339779 |
| AHSG | Alpha 2-HS Glycoprotein | Protein Coding | P02765 | 3.174816132 |
| MBL2 | Mannose Binding Lectin 2 | Protein Coding | P11226 | 3.172001123 |
| EDNRA | Endothelin Receptor Type A | Protein Coding | P25101 | 3.141683817 |
| MEF2C | Myocyte Enhancer Factor 2C | Protein Coding | Q06413 | 3.141683817 |
| FGF23 | Fibroblast Growth Factor 23 | Protein Coding | Q9GZV9 | 3.141683817 |
| LDLR | Low Density Lipoprotein Receptor | Protein Coding | P01130 | 3.078409433 |
| EDNRB | Endothelin Receptor Type B | Protein Coding | P24530 | 3.078409433 |
| RETN | Resistin | Protein Coding | Q9HD89 | 3.078409433 |
| FGG | Fibrinogen Gamma Chain | Protein Coding | P02679 | 3.045286655 |
| DKK1 | Dickkopf WNT Signaling Pathway Inhibitor 1 | Protein Coding | O94907 | 3.040119171 |
| RND3 | Rho Family GTPase 3 | Protein Coding | P61587 | 3.014294863 |
| BNC2 | Basonuclin Zinc Finger Protein 2 | Protein Coding | Q6ZN30 | 3.014294863 |
| CPNE8 | Copine 8 | Protein Coding | Q86YQ8 | 3.014294863 |
| C4orf33 | Chromosome 4 Open Reading Frame 33 | Protein Coding | Q8N1A6 | 3.014294863 |
| PTX3 | Pentraxin 3 | Protein Coding | P26022 | 2.975547075 |
| FGA | Fibrinogen Alpha Chain | Protein Coding | P02671 | 2.938660622 |
| ECE1 | Endothelin Converting Enzyme 1 | Protein Coding | P42892 | 2.938660622 |
| NPR3 | Natriuretic Peptide Receptor 3 | Protein Coding | P17342 | 2.938660622 |
| ECE2 | Endothelin Converting Enzyme 2 | Protein Coding | P0DPD6 | 2.938660622 |
| REN | Renin | Protein Coding | P00797 | 2.900202274 |

**Table S11 Molecular docking results**

| **Metabolites** | **PubChem CID** | **Target** | **PDB_ID** | **Energy (kcal/mol)** |
| --- | --- | --- | --- | --- |
| UNII-0TM46496W4 | 12778780 | ROCK2 | 6P5P | -8.0 |
| UNII-0TM46496W4 | 12778780 | CYP11B2 | 6XZ8 | -10.1 |
| Sucrose | 5988 | ADRA1A | 8HN1 | -7.2 |
| Sucrose | 5988 | ADRA2B | 6K42 | -8.1 |
| Octanoylcarnitine | 123701 | MMP2 | 1CK7 | -6.5 |
| Octanoylcarnitine | 123701 | AGTR1 | 6DO1 | -6.6 |
| Decanoyl L−Carnitine | 11953821 | MMP2 | 1CK7 | -5.7 |
| Decanoyl L−Carnitine | 11953821 | AGTR1 | 6DO1 | -5.3 |
| Carnitine C8:0 | 11953814 | MMP2 | 1CK7 | -6.5 |
| Carnitine C8:0 | 11953814 | AGTR1 | 6DO1 | -5.9 |
| Carnitine C12:1 | 165415714 | MMP2 | 1CK7 | -7.6 |
| Carnitine C12:1 | 165415714 | AGTR1 | 6DO1 | -6.2 |
| Carnitine C12:1 | 165415714 | ACE | 6ZPT | -6.2 |
| Carnitine C12:0 | 10427569 | MMP2 | 1CK7 | -6.4 |
| Carnitine C11:1 | 3072516 | MMP2 | 1CK7 | -6.5 |
| Carnitine C10:1 | 53481651 | MMP2 | 1CK7 | -6.3 |
| Carnitine C10:1 | 53481651 | AGTR1 | 6DO1 | -5.8 |
| Carnitine C10:1 | 53481651 | ACE | 6ZPT | -5.4 |
| Carnitine C10:0 | 10245190 | MMP2 | 1CK7 | -6.2 |
| Carnitine C10:0 | 10245190 | AGTR1 | 6DO1 | -5.8 |
| 9,10,13-TriHOME | 14968868 | TNF | 4Y6O | -6.7 |
| 12-hydroxyeicosatetraenoic acid | 13786989 | MMP2 | 1CK7 | -7.7 |
| 12-hydroxyeicosatetraenoic acid | 13786989 | AGTR1 | 6DO1 | -6.6 |
| 12-hydroxyeicosatetraenoic acid | 13786989 | ACE | 6ZPT | -6.8 |
