# Supplementary material for: Apiotrichum‐Centered Fungal Interaction Network Disturbance Correlates With Increased Arterial Stiffness
Source: Aging Cell. 2026 Jul 25;25(8):e70650. doi: 10.1111/acel.70650 (PMC13401687; doi:10.1111/acel.70650)
Supplement: Supplementary file 2 — Data S1: acel70650‐sup‐0002‐Supinfo1.docx. [file ACEL-25-e70650-s001.docx]

**STROBE-MR checklist of recommended items to address in reports of Mendelian randomization studies**^1^ ^2^

| **Item No.** | **Section** | **Checklist item** | **Page No.** | **Relevant text from manuscript** |
| --- | --- | --- | --- | --- |
| 1 | **TITLE and ABSTRACT** | Indicate Mendelian randomization (MR) as the study’s design in the title and/or the abstract if that is a main purpose of the study | 3 | **KEYWORDS**  Gut mycobiome; Arterial stiffness; Apiotrichum; multi-omics integration; Mendelian randomization |
|  | **INTRODUCTION** |  |  |  |
| 2 | **Background** | Explain the scientific background and rationale for the reported study. What is the exposure? Is a potential causal relationship between exposure and outcome plausible? Justify why MR is a helpful method to address the study question | 4-5 | Recently, the gut microbiome…has been identified as an emerging modulator in vascular health…few projects have examined the role of intestinal fungi…in metabolic health. To address this gap, we performed an integrative analysis of the gut mycobiome, host genetics, and plasma metabolites… (Exposure: gut mycobiome; outcome: arterial stiffness measured by baPWV). |
| 3 | **Objectives** | State specific objectives clearly, including pre-specified causal hypotheses (if any). State that MR is a method that, under specific assumptions, intends to estimate causal effects | 5 | To address this gap, we performed an integrative analysis of the gut mycobiome, host genetics, and plasma metabolites in a cohort of 763 community residents…and uncovered the potential molecular mechanisms whereby altered gut mycobiome modulated arterial stiffness. |
|  | **METHODS** |  |  |  |
| 4 | **Study design and data sources** | Present key elements of the study design early in the article. Consider including a table listing sources of data for all phases of the study. For each data source contributing to the analysis, describe the following: |  | Local residents who had lived in Guangdong province, China, for over five years were invited to participate in health screenings conducted at the Community Healthcare Centre of Chashan Town (Dongguan City, Guangdong, China) and Guangzhou (Guangdong, China) through flyers and posters. |
|  | a) | Setting: Describe the study design and the underlying population, if possible. Describe the setting, locations, and relevant dates, including periods of recruitment, exposure, follow-up, and data collection, when available. | 5 | Local residents who had lived in Guangdong province, China, for over five years were invited to participate in health screenings conducted at the Community Healthcare Centre of Chashan Town (Dongguan City, Guangdong, China) and Guangzhou (Guangdong, China) through flyers and posters. |
|  | b) | Participants: Give the eligibility criteria, and the sources and methods of selection of participants. Report the sample size, and whether any power or sample size calculations were carried out prior to the main analysis | 5 | Finally, a total of 763 participants with internal transcribed spacer 1 (ITS1) sequencing data were included in the analysis, of whom 682 had paired metabolomics data and 752 had host genomics data. |
|  | c) | Describe measurement, quality control and selection of genetic variants | 8, Suppl. 7 | All genotyping utilized the Infinium Chinese Genotyping Array‑24 v1.0 BeadChip (Illumina platform). Quality control was conducted with PLINK (v.1.9), and single‑nucleotide polymorphisms (SNPs) were excluded based on the following criteria: (1) Minor Allele Frequency < 5%; (2) Hardy‑Weinberg equilibrium violation with P < 0.00001, and (3) genotype calling rate < 5%. linkage disequilibrium (LD) was calculated for each pair of SNPs over a window of 50 SNPs, with one SNP of any pair removed if the LD exceeded 0.5. |
|  | d) | For each exposure, outcome, and other relevant variables, describe methods of assessment and diagnostic criteria for diseases | 6 | Exposure (gut mycobiome): ITS1 sequencing (described). Outcome (arterial stiffness): baPWV measured with automatic waveform analyzer; “Subjects were categorized into groups with elevated arterial stiffness (defined as baPWV ≥ 1400 cm/s) and normal arterial stiffness (baPWV < 1400 cm/s).” Covariates: anthropometrics, blood pressure, questionnaire, dietary diversity score, China‑PAR equation. |
|  | e) | Provide details of ethics committee approval and participant informed consent, if relevant | 24 | This study was approved by the Ethics Committee of the School of Public Health at Sun Yat-sen University (2017-001), and was in accordance with the principles of the Declaration of Helsinki. Written informed consent was obtained from each participant. |
| 5 | **Assumptions** | Explicitly state the three core IV assumptions for the main analysis (relevance, independence and exclusion restriction) as well assumptions for any additional or sensitivity analysis | Suppl. 7 | SNPs with an F statistic (beta2/SE2)>10 were considered as strong genetic instrumental variables (IVs) and were utilized for subsequent MR analysis. … To ensure the validity of the results, potential causal estimates were selected based on the following criteria: (1) P < 0.05 calculated by the IVW method; (2) consistent direction in effect sizes across the three methods; and (3) P>0.05 for the intercept term calculated by the MR-Egger method, indicating no pleiotropic effects. … Moreover, to identify potential heterogeneous SNPs, a ‘leave-one-out’ analysis was performed by omitting each instrumental SNP in turn. |
| 6 | **Statistical methods: main analysis** | Describe statistical methods and statistics used |  |  |
|  | a) | Describe how quantitative variables were handled in the analyses (i.e., scale, units, model) | 6 | baPWV analysed as binary (≥1400 cm/s) and continuous (cm/s). Fungal genera relative abundances normalised by cumulative sum scaling (MetagenomeSeq). Metabolites normalised by total sum scaling. |
|  | b) | Describe how genetic variants were handled in the analyses and, if applicable, how their weights were selected | Suppl. 7 | The threshold of P < 5×10⁻⁵ was set for identifying mycobiome‑associated SNPs…SNPs with an F statistic (beta²/SE²) > 10 were considered as strong genetic instrumental variables (IVs). |
|  | c) | Describe the MR estimator (e.g. two-stage least squares, Wald ratio) and related statistics. Detail the included covariates and, in case of two-sample MR, whether the same covariate set was used for adjustment in the two samples | Suppl. 7 | MR estimates were calculated using IVW methods with a random effects model. In addition, we reported MR estimates using the weighted median and MR‑Egger regression methods. |
|  | d) | Explain how missing data were addressed |  | Not stated. |
|  | e) | If applicable, indicate how multiple testing was addressed | 9 | All multiple comparisons incorporated Benjamini‑Hochberg false discovery rate correction (Padj), except where specified. |
| 7 | **Assessment of assumptions** | Describe any methods or prior knowledge used to assess the assumptions or justify their validity | 13 | MR‑Egger regression detected no significant horizontal pleiotropy (intercept P > 0.05; Supplementary Table 5). Leave‑one‑out analysis confirmed that no single nucleotide polymorphism drove the observed association (Supplementary Figure 3). |
| 8 | **Sensitivity analyses and additional analyses** | Describe any sensitivity analyses or additional analyses performed (e.g. comparison of effect estimates from different approaches, independent replication, bias analytic techniques, validation of instruments, simulations) | Suppl. 7 | Bidirectional MR; multiple MR methods (IVW, weighted median, MR‑Egger); leave‑one‑out analysis; propensity score matching; mediation analyses. |
| 9 | **Software and pre-registration** |  |  |  |
|  | a) | Name statistical software and package(s), including version and settings used | 9, Suppl. 7 | R (version 4.4.1), PLINK (v.1.9), MendelianRandomization (version 0.9.0), vegan (version 2.6‑4), igraph (version 1.4.3), MaAsLin2, Gephi 0.10, AutodockVina 1.2.2, antiSMASH 8.0.4. |
|  | b) | State whether the study protocol and details were pre-registered (as well as when and where) |  | Not stated |
|  | **RESULTS** |  |  |  |
| 10 | **Descriptive data** |  |  |  |
|  | a) | Report the numbers of individuals at each stage of included studies and reasons for exclusion. Consider use of a flow diagram | 5 | a total of 763 participants with ITS1 sequencing data were included…682 had paired metabolomics data and 752 had host genomics data. |
|  | b) | Report summary statistics for phenotypic exposure(s), outcome(s), and other relevant variables (e.g. means, SDs, proportions) | 38 | Table 1 summarises all relevant clinical characteristics (median, IQR, proportions) for normal and elevated arterial stiffness groups. |
|  | c) | If the data sources include meta-analyses of previous studies, provide the assessments of heterogeneity across these studies |  | Not applicable (single cohort). |
|  | d) | For two-sample MR:  i.  Provide justification of the similarity of the genetic variant-exposure associations between the exposure and outcome samples  ii.  Provide information on the number of individuals who overlap between the exposure and outcome studies |  | Not applicable (one‑sample MR). |
| 11 | **Main results** |  |  |  |
|  | a) | Report the associations between genetic variant and exposure, and between genetic variant and outcome, preferably on an interpretable scale | Suppl. 26 - 33 | SNP selection and F‑statistics are reported in Supplementary Tables 4‑5. SNP‑outcome associations are not separately tabulated but are integrated in the MR estimates. |
|  | b) | Report MR estimates of the relationship between exposure and outcome, and the measures of uncertainty from the MR analysis, on an interpretable scale, such as odds ratio or relative risk per SD difference | 12 | *Apiotrichum* significantly increased baPWV (BetaIVW = 0.007, 95% CI = 0.003 to 0.010, P < 0.05). *Mycothermus* (BetaIVW = –0.012, 95% CI = –0.016 to –0.008, P < 0.05) reduced arterial stiffness。 |
|  | c) | If relevant, consider translating estimates of relative risk into absolute risk for a meaningful time period |  | Not applicable (cross‑sectional, no time‑to‑event). |
|  | d) | Consider plots to visualize results (e.g. forest plot, scatterplot of associations between genetic variants and outcome versus between genetic variants and exposure) | 35, Suppl. 14-15 | Forest plot in Figure 2D; leave‑one‑out and scatter plots in Supplementary Figures 3‑4. |
| 12 | **Assessment of assumptions** |  |  |  |
|  | a) | Report the assessment of the validity of the assumptions | 13 | all instrumental variables exceeded the critical threshold (F‑statistics > 10), eliminating weak instrument bias.” “MR‑Egger regression detected no significant horizontal pleiotropy (intercept P > 0.05; Supplementary Table 5). |
|  | b) | Report any additional statistics (e.g., assessments of heterogeneity across genetic variants, such as *I^2^*, Q statistic or E-value) |  | Not stated |
| 13 | **Sensitivity analyses and additional analyses** |  |  |  |
|  | a) | Report any sensitivity analyses to assess the robustness of the main results to violations of the assumptions | 13 | MR‑Egger, weighted median, leave‑one‑out analyses are reported. |
|  | b) | Report results from other sensitivity analyses or additional analyses | Suppl.  6 | Propensity score‑matched analyses and mediation analyses are reported. |
|  | c) | Report any assessment of direction of causal relationship (e.g., bidirectional MR) | 13 | Reverse MR analysis indicated no potential causal effects of arterial stiffness on Flavocillium or Microscypha. Although significant associations appeared for Apiotrichum, Mycothermus, and Hyaloscypha in IVW analysis, significant MR‑Egger intercept terms (Supplementary Figure 4 and Supplementary Table 6) invalidated these reverse causal interpretations. |
|  | d) | When relevant, report and compare with estimates from non-MR analyses | Suppl.  6 | Associations from multivariable regression (MaAsLin2) are reported alongside MR estimates. |
|  | e) | Consider additional plots to visualize results (e.g., leave-one-out analyses) | Suppl.  14 | Leave‑one‑out plots are provided in Supplementary Figure 3. |
|  | **DISCUSSION** |  |  |  |
| 14 | **Key results** | Summarize key results with reference to study objectives | 17 | Key fungal genera, including *Apiotrichum*, *Mycothermus*, *Flavocillium*, *Hyaloscypha*, and *Microscypha*, were potentially causally linked to increased baPWV levels. Moreover, through an integration of multi‑omics and in silico analysis, increased UNII‑0TM46496W4, 12‑hydroxyeicosatetraenoic acid, and sucrose were identified as molecular transducers linking *Apiotrichum* to elevated arterial stiffness. |
| 15 | **Limitations** | Discuss limitations of the study, taking into account the validity of the IV assumptions, other sources of potential bias, and imprecision. Discuss both direction and magnitude of any potential bias and any efforts to address them | 23 | Our participants were exclusively Chinese recruited from a single center, and thus further validation in larger and more diverse ethnic groups are warranted. This study was cross‑sectional, limiting its ability to establish causal inferences. Although integrative analysis and in silico analysis improved the biological understanding…further validation in animal models was warranted |
| 16 | **Interpretation** |  |  |  |
|  | a) | Meaning: Give a cautious overall interpretation of results in the context of their limitations and in comparison with other studies | 17 | Cautious interpretation given throughout the Discussion, with comparisons to previous reports including *Apiotrichum* in hypertension, *Mycothermus* producing xylooligosaccharides. |
|  | b) | Mechanism: Discuss underlying biological mechanisms that could drive a potential causal relationship between the investigated exposure and the outcome, and whether the gene-environment equivalence assumption is reasonable. Use causal language carefully, clarifying that IV estimates may provide causal effects only under certain assumptions | 17 | Detailed mechanistic discussion of fungal metabolites (UNII‑0TM46496W4, 12‑HETE, carnitines, sucrose) and downstream targets (CYP11B2, ROCK2, MMP2, ADRA1A, ADRA2B) is provided. Causal language is used with appropriate caveats. The MR assumptions are not explicitly revisited in the Discussion. |
|  | c) | Clinical relevance: Discuss whether the results have clinical or public policy relevance, and to what extent they inform effect sizes of possible interventions | 23 | Our study uncovers a strong association between disturbance in gut fungi and the deterioration of arterial stiffness…highlights the potential of targeting the gut mycobiome for improving vascular health. |
| 17 | **Generalizability** | Discuss the generalizability of the study results (a) to other populations, (b) across other exposure periods/timings, and (c) across other levels of exposure | 23 | Our participants were exclusively Chinese recruited from a single center, and thus further validation in larger and more diverse ethnic groups are warranted. |
|  | **OTHER INFORMATION** |  |  |  |
| 18 | **Funding** | Describe sources of funding and the role of funders in the present study and, if applicable, sources of funding for the databases and original study or studies on which the present study is based | 25 | This work was supported by National Key Research and Development Program of China (2023YFC3606300), Joint Funds of the National Natural Science Foundation of China (No. U24A20769), Young Scientists Fund of the National Natural Science Foundation of China (No. 82404256), the National Natural Science Foundation of China (No. 82273611), and Fundamental Research Funds for the Central University, Sun Yat-sen University (2025QNPY01). |
| 19 | **Data and data sharing** | Provide the data used to perform all analyses or report where and how the data can be accessed, and reference these sources in the article. Provide the statistical code needed to reproduce the results in the article, or report whether the code is publicly accessible and if so, where | 25 | TS sequencing data from this study has been deposited at the NCBI Sequence Read Archive under BioProject: PRJNA1273442. The metabolomics raw data have been deposited in the MetaboLights database under accession number REQ20260522219902. This study doesn’t generate any new codes. Any additional information required to reanalyze the data reported in this paper is available from the lead contact upon reasonable request. |
| 20 | **Conflicts of Interest** | All authors should declare all potential conflicts of interest | 24 | No conflict of interest |

This checklist is copyrighted by the Equator Network under the Creative Commons Attribution 3.0 Unported (CC BY 3.0) license.

1. Skrivankova VW, Richmond RC, Woolf BAR, Yarmolinsky J, Davies NM, Swanson SA, et al. Strengthening the Reporting of Observational Studies in Epidemiology using Mendelian Randomization (STROBE-MR) Statement. JAMA. 2021;under review.

2. Skrivankova VW, Richmond RC, Woolf BAR, Davies NM, Swanson SA, VanderWeele TJ, et al. Strengthening the Reporting of Observational Studies in Epidemiology using Mendelian Randomisation (STROBE-MR): Explanation and Elaboration. BMJ. 2021;375:n2233.
